# Restores Aβ Clearance by Overcoming PCSK9–LRP1 Dysregulation and TRIB3‐Mediated Autophagy Blockade in Alzheimer's Disease

**DOI:** 10.1002/advs.77929

**Published:** 2026-09-29

**Authors:** Jie Miao, Jing Wang, Yankun Li, Haipei Zhang, Yanli Zhang, Qingnian Li, Bo Xiao, Wenhu Zhou, Junhong Guo

**Affiliations:** ^1^ Department of Neurology First Hospital of Shanxi Medical University Taiyuan Shanxi China; ^2^ The First Clinical Medical School of Shanxi Medical University Taiyuan Shanxi China; ^3^ Department of Neurology Xiangya Hospital Central South University Changsha Hunan China; ^4^ National Clinical Research Center for Geriatric Diseases, Xiangya Hospital Central South University Changsha Hunan China; ^5^ Xiangya School of Pharmaceutical Sciences Central South University Changsha Hunan China; ^6^ Department of Neurology Sixth Hospital of Shanxi Medical University (General Hospital of Tisco) Taiyuan Shanxi China; ^7^ Hunan Key Laboratory of The Research and Development of Novel Pharmaceutical Preparations, School of Pharmaceutical Science Changsha Medical University Changsha Hunan China

**Keywords:** alzheimer's disease, amyloid‐β clearance, blood–brain barrier, PCSK9, siRNA delivery, tea polyphenol nanoparticles (TPNs), TRIB3

## Abstract

Alzheimer's disease (AD) is characterized by amyloid‐β (Aβ) accumulation, neuroinflammation, and vascular dysfunction, yet effective therapies remain limited. Impaired Aβ clearance across the blood–brain barrier (BBB) is a key contributor to AD pathogenesis. Two sequential barriers to Aβ clearance are identified: proprotein convertase subtilisin/kexin type 9 (PCSK9) upregulation in cerebrovascular endothelial cells compromises low‐density lipoprotein receptor‐related protein 1 (LRP1)‐mediated Aβ efflux, whereas increased intracellular Aβ handling after PCSK9 silencing induces tribbles pseudokinase 3 (TRIB3) upregulation and exposes an autophagy blockade restricting intracellular Aβ degradation. PCSK9 silencing restores LRP1 expression and enhances Aβ uptake and efflux, whereas TRIB3 knockdown restores autophagic flux and facilitates Aβ degradation. SITR (siPCSK9/siTRIB3@TPN‐RAP), a RAP‐modified siRNA nanodelivery system based on tea polyphenol nanoparticles (TPNs), enables brain‐enriched co‐delivery of siPCSK9 and siTRIB3. SITR enhances BBB penetration and preferentially accumulates in cerebrovascular endothelial cells and microglia. In APP/PS1 mice, SITR improves cognitive performance, reduces Aβ and cerebral amyloid angiopathy burden, preserves vascular and neuronal homeostasis, and suppresses neuroinflammation, while showing no overt short‐term systemic toxicity under a 6‐week regimen. These findings establish PCSK9 and TRIB3 as complementary therapeutic targets and support SITR as an effective nanoplatform integrating enhanced Aβ efflux with restored autophagic degradation for AD intervention.

## Graphical abstract

PCSK9 upregulation in cerebrovascular endothelial cells and microglia destabilizes the Aβ transporter LRP1, while TRIB3‐associated autophagy blockade further limits intracellular Aβ degradation. A RAP‐modified tea‐polyphenol nanoparticle co‐delivers siPCSK9 and siTRIB3 to restore LRP1‐mediated Aβ efflux and autophagy‐dependent degradation, reducing Aβ deposition, cerebral amyloid angiopathy, BBB disruption, and neuroinflammation in APP/PS1 mice.

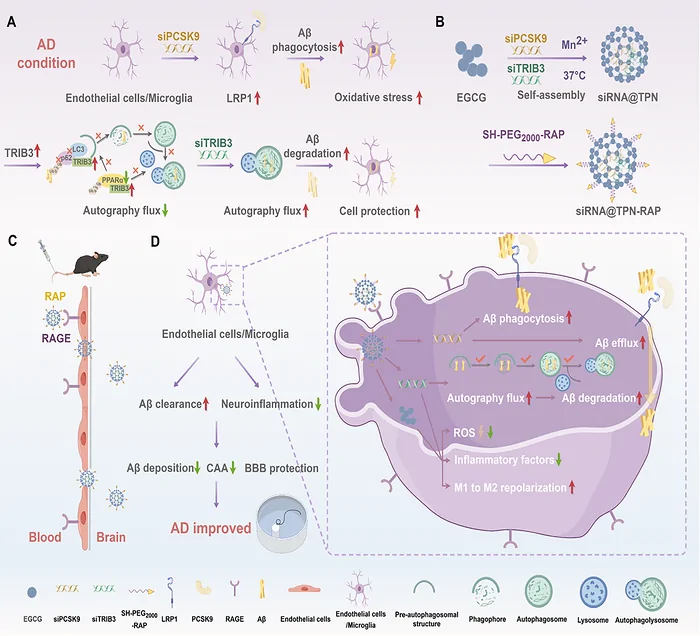

## 1 Introduction

Alzheimer's disease (AD) is a progressive neurodegenerative disorder that predominantly affects the elderly and is the leading cause of dementia worldwide [1, 2]. With the rapid aging of the global population, the prevalence of AD is rising sharply, imposing a heavy burden on patients, caregivers, and healthcare systems [3, 4]. Despite intense research efforts, available drugs provide only modest symptomatic relief and fail to halt or reverse disease progression [1]. The pathological mechanisms of AD are multifactorial, among which aberrant accumulation of amyloid‐β (Aβ) peptides is a defining hallmark [5, 6, 7]. Aβ_1–42_ preferentially aggregates within the brain parenchyma to form neurotoxic plaques, while Aβ_1–40_ predominantly deposits along cerebral blood vessel walls, contributing to cerebral amyloid angiopathy (CAA) [5, 6, 7]. Clinical and pathological studies demonstrate that Aβ deposition begins years before the onset of cognitive decline, and the burden of Aβ correlates positively with disease severity [5]. Thus, enhancing Aβ clearance has emerged as a central therapeutic strategy [1, 5].

Current Aβ‐directed disease‐modifying therapies (DMTs) fall into three major categories: reducing Aβ production, inhibiting its aggregation, or promoting clearance, particularly through immunotherapy [1, 8]. However, their clinical translation has been hampered by limited efficacy and adverse effects. Numerous trials targeting Aβ biosynthesis or aggregation have failed [8]. Although monoclonal antibodies such as Aducanumab and Lecanemab reduce plaque burden and modestly slow cognitive decline, they are associated with a high incidence of amyloid‐related imaging abnormalities (ARIA), including vasogenic edema (ARIA‐E) and microhemorrhages (ARIA‐H) [7, 9, 10, 11]. These adverse events are linked to increased vascular permeability, blood–brain barrier (BBB) disruption, and local inflammatory responses, often exacerbated by pre‐existing CAA [7, 10, 12]. In addition, antibody therapies suffer from low BBB penetration and potential off‐target effects [12]. Given the complex interplay among Aβ deposition, neuroinflammation, and vascular injury, single‐pathway interventions provide limited benefit, underscoring the need for safe, multi‐target therapeutic strategies [13, 14, 15].

Under physiological conditions, brain Aβ homeostasis depends on a balance between production and clearance. Approximately 85% of Aβ is eliminated via BBB‐mediated efflux [16], primarily through low‐density lipoprotein receptor‐related protein 1 (LRP1), expressed on cerebrovascular endothelial cells [15, 16, 17]. Microglia also utilize LRP1 to recognize and engulf Aβ_1–42_ [18, 19]. In AD, clearance capacity is impaired: the rate of Aβ removal is reduced by ∼30% compared to healthy controls, without significant changes in production [6]. Decreased endothelial LRP1 expression directly weakens efflux efficiency [15, 20]. Restoring LRP1‐mediated Aβ transport is therefore an attractive therapeutic strategy. However, excessive uptake without sufficient lysosomal degradation capacity risks intracellular Aβ accumulation, lysosomal damage, and cytotoxicity. This contributes to CAA, BBB breakdown, and microglial overactivation [15, 21, 22, 23]. Hence, optimal interventions should enhance Aβ efflux while simultaneously promoting intracellular degradation. Autophagy, a lysosome‐dependent clearance pathway, degrades protein aggregates such as Aβ and regulates neuroinflammation [24, 25, 26, 27]. In AD, autophagic flux is impaired, exacerbating Aβ deposition [28, 29]. Thus, combined enhancement of LRP1‐mediated efflux and autophagy may synergistically promote Aβ clearance while mitigating downstream pathologies.

Proprotein convertase subtilisin/kexin type 9 (PCSK9) has emerged as an important regulator of LRP1 stability and Aβ clearance [30]. By promoting lysosomal degradation of LRP1, PCSK9 may reduce the endothelial and microglial capacity for Aβ uptake and transport [30]. Therefore, PCSK9 silencing represents a rational strategy to restore LRP1‐dependent Aβ efflux. However, enhancing Aβ uptake/efflux without simultaneously improving intracellular degradative capacity may impose an additional proteostatic burden on cells [28, 31]. Tribbles pseudokinase 3 (TRIB3) is a stress‐responsive pseudokinase that suppresses autophagy by destabilizing PPARα and interfering with p62‐mediated autophagic flux [32, 33, 34]. We therefore hypothesized that PCSK9 and TRIB3 may represent two functionally complementary targets acting at sequential steps of Aβ clearance: PCSK9 controls LRP1‐dependent Aβ uptake/efflux, whereas TRIB3 limits intracellular autophagic degradation. Thus, dual silencing of PCSK9 and TRIB3 using siRNAs (siPCSK9 + siTRIB3) could provide a synergistic strategy in which siPCSK9 restores LRP1‐mediated Aβ efflux and siTRIB3 reactivates autophagic degradation of Aβ, thereby reducing Aβ burden, neuroinflammation, and BBB injury (Scheme 1A).

**SCHEME 1:**
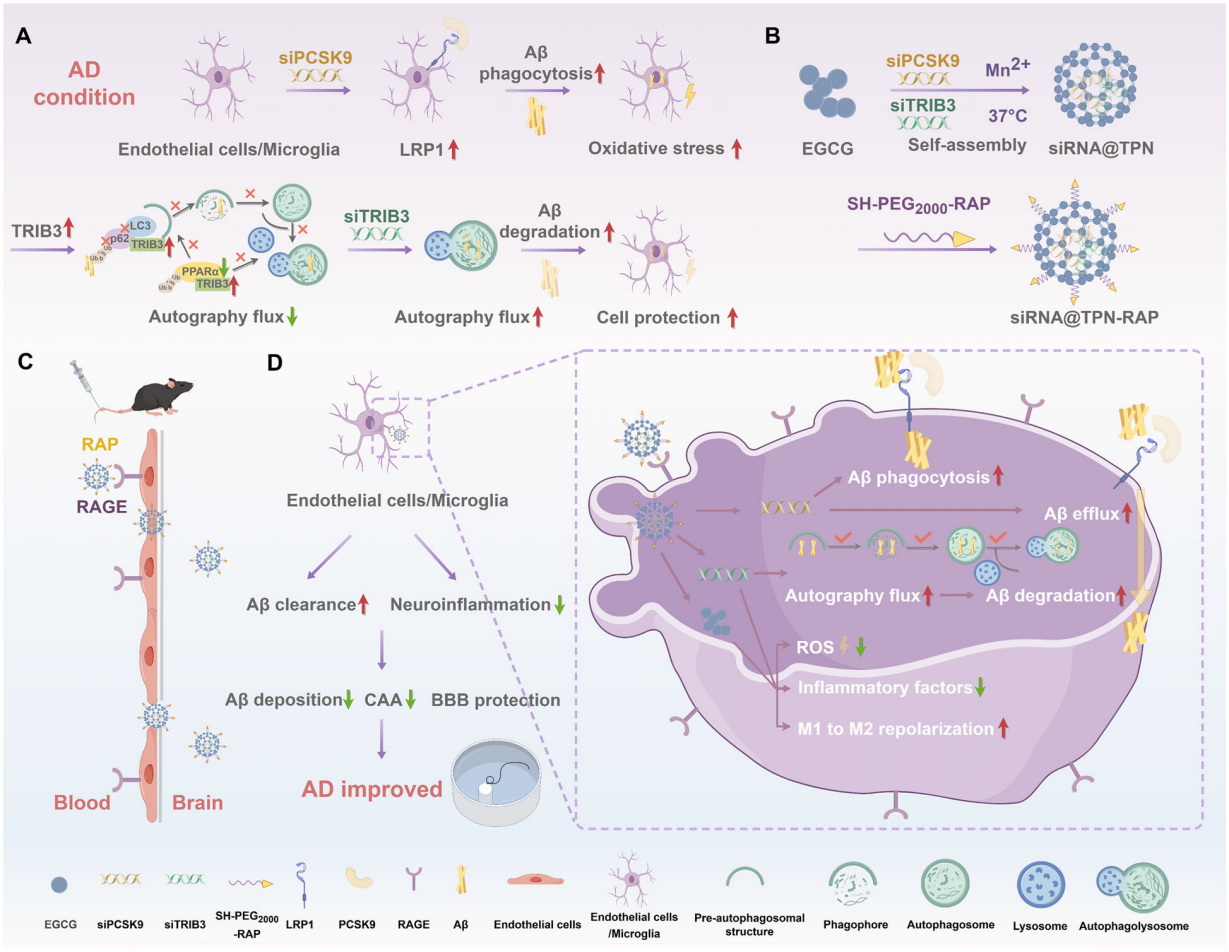
Design and mechanism of the dual‐target siRNA nanodelivery system (SITR) for Alzheimer's disease therapy. (A) Schematic illustration of synergistic silencing of PCSK9 and TRIB3 to enhance Aβ clearance and protect neurovascular function. siPCSK9 prevents PCSK9‐mediated degradation of LRP1, thereby promoting trans‐BBB Aβ efflux, while siTRIB3 restores autophagic degradation, accompanied by increased PPARα expression and reduced p62 accumulation, thereby facilitating intracellular Aβ degradation, attenuating pro‐inflammatory signaling, and preserving BBB integrity. (B) Construction of SITR: during self‐assembly of tea polyphenol nanoparticles (TPNs), siPCSK9 and siTRIB3 are co‐encapsulated to form SIT; subsequent surface modification with SH‐PEG_2000_‐RAP yields SITR. (C) Targeted delivery mechanism of SITR: RAP peptide modification enables specific recognition of RAGE, which is upregulated on cerebrovascular endothelial cells and microglia in AD, thereby enhancing BBB penetration, brain accumulation, and cell targeting. (D) Mechanistic overview of SITR action in AD therapy, highlighting synergistic enhancement of Aβ efflux, autophagy‐dependent Aβ degradation, alleviation of neuroinflammation, and protection of vascular and neuronal homeostasis. The figure was originally drawn using FigDraw.

Nevertheless, siRNA therapeutics face formidable delivery challenges, including instability in circulation, poor BBB penetration, and inefficient uptake by target cells [35]. To address these limitations, we designed a multifunctional nanodelivery system (siPCSK9/siTRIB3@TPN‐RAP, SITR) based on tea polyphenol nanoparticles (TPNs) (Scheme 1B). siPCSK9 and siTRIB3 were co‐encapsulated during TPNs self‐assembly to form siPCSK9/siTRIB3@TPN (SIT), followed by surface conjugation with RAP, a peptide antagonist of the receptor for advanced glycation end products (RAGE), via SH‐PEG_2000_ linkers to generate SITR. Given that RAGE is upregulated in cerebrovascular endothelial cells and microglia in AD, RAP modification enables SITR to efficiently cross the BBB and accumulate in these target cells via targeting RAGE (Scheme 1C). In this way, SITR combines “brain‐enriched delivery” with “dual‐target therapy.” Specifically, siPCSK9 prevents PCSK9‐mediated degradation of LRP1, enhancing trans‐BBB Aβ efflux, while siTRIB3 relieves autophagy inhibition, restores intracellular Aβ degradation, and reduces neuroinflammatory responses (Scheme 1D).

In this study, we systematically investigated the therapeutic efficacy, mechanisms of action, in vivo biodistribution, metabolic consequences, and short‐term systemic safety of SITR in APP/PS1 transgenic mice. This design allowed us to determine whether integrating enhanced Aβ efflux with restored autophagic degradation could overcome key limitations of single‐pathway Aβ clearance strategies.

## 2 Results and Discussion

### 2.1 Upregulation of PCSK9 in Cerebrovascular Endothelial Cells and Microglia of AD Patients and its Regulatory Role in Aβ Clearance

To investigate the pathological mechanisms of AD, we analyzed a single‐nucleus RNA sequencing (snRNA‐seq) dataset from middle temporal gyrus tissue (GSE188545) [36], which included samples from six AD patients (Braak V/VI) and six healthy controls (HC, Braak I/II). Cell subtype‐specific analysis demonstrated that PCSK9 expression was significantly upregulated in both cerebrovascular endothelial cells and microglia in AD patients (Figure 1A–E). As a key regulator of lipid transport and LRP1 degradation, the elevated expression of PCSK9 suggests its potential role in exacerbating Aβ metabolic dysregulation through impaired LRP1‐mediated clearance pathways.

**FIGURE 1:**
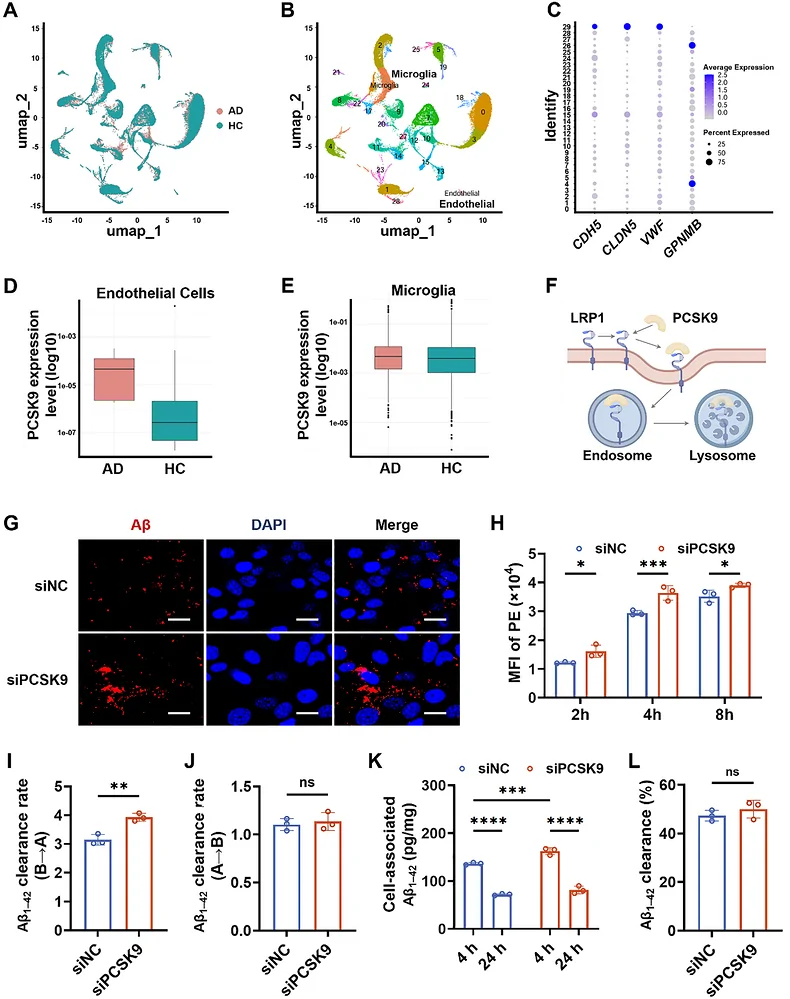
Expression characteristics of PCSK9 in AD pathology and regulatory effects of siPCSK9 on Aβ clearance. (A) Integrated UMAP visualization of all cells from middle temporal gyrus samples of AD patients and HC. (B) UMAP plot of integrated data depicts all annotated cell types, highlighting microglia and cerebrovascular endothelial cells as distinct populations. (C) Identification of key subpopulations by marker gene expression: cerebrovascular endothelial cells (*CDH5/CLDN5/VWF*) and microglia (*GPNMB*). (D,E) Boxplots showing PCSK9 expression levels in cerebrovascular endothelial cells (D) and microglia (E) from middle temporal gyrus samples of AD patients (*n* = 6 donors) and HC (*n* = 6 donors). Endothelial cells: AD *n* = 9,234 cells, HC *n* = 8,371 cells. Microglia: AD *n* = 3,751 cells, HC *n* = 4,291 cells. (F) Schematic illustration of PCSK9‐mediated lysosomal degradation of LRP1. Created using FigDraw. Adapted from Mazura and Pietrzik [37] under the terms of the Creative Commons Attribution 4.0 International License (CC BY 4.0; https:// creativecommons.org/licenses/by/ 4.0/). 2023 by the authors; licensee MDPI, Basel, Switzerland. (G) Confocal fluorescence images of 5‐TAMRA‐Aβ_1–42_ uptake in bEnd.3 cells with or without siPCSK9 treatment (red: Aβ; blue: nuclei). Scale bar = 20 µm. (H) Flow cytometry quantification of 5‐TAMRA‐Aβ_1–42_ uptake over time at 37°C in bEnd.3 cells treated with siPCSK9. (I,J) Transwell BBB transport assays evaluating Aβ_1–42_ clearance transport from the brain side to blood side (B → A, I) and influx from blood to brain side (A → B, J) following siPCSK9 intervention. (K) ELISA quantification of intracellular Aβ_1–42_ levels in cerebrovascular endothelial cells at different time points after siPCSK9 treatment. (L) Statistical analysis of Aβ_1–42_ degradation rate within 24 h post‐siPCSK9 treatment. Boxplots in (D,E) show the median (center line), interquartile range (IQR, box limits: 25th‐75th percentiles), and whiskers extending to 1.5 x IQR. Outliers are plotted as individual points. Quantitative data in (H–L) are presented as mean ± SD (*n* = 3 independent experiments). Wilcoxon Rank Sum Test for (D,E). Two‐way ANOVA followed by Tukey's multiple‐comparison test for (H,K); unpaired two‐tailed Student's *t*‐test for (I,J,L). ns, not significant; ^*^ *p* < 0.05, ^**^ *p* < 0.01, ^***^ *p* < 0.001, ^****^ *p* < 0.0001.

To investigate whether Aβ regulates PCSK9 expression, we performed RNA sequencing of human cerebral microvascular endothelial cells (hCMEC/D3) and human microglia (HMC3) following Aβ_1–42_ treatment. Both cell types exhibited a significant upregulation of PCSK9 expression (Figure S1A,B). Consistent results were obtained in mouse brain microvascular endothelial cells (bEnd.3) and mouse microglia (BV2), where recombinant human Aβ_1–42_ induced *Pcsk9* mRNA expression while suppressing *Lrp1* mRNA expression (Figure S2A–D). These findings suggest that Aβ may inhibit LRP1‐mediated clearance by inducing PCSK9 expression.

We next employed RNA interference to silence PCSK9. Among the tested siRNAs, siPCSK9‐3 achieved the highest knockdown efficiency (Figure S3) and was used in subsequent experiments. Previous studies have shown that PCSK9 promotes lysosomal degradation of LRP1, thereby reducing its surface abundance, as schematically illustrated in Figure 1F [30, 37].

Western blot (WB) analysis confirmed that siPCSK9 significantly decreased PCSK9 protein levels while increasing LRP1 expression in both bEnd.3 (Figure S4A–C) and BV2 cells (Figure S5A–C), indicating that PCSK9 silencing stabilizes LRP1 by preventing its degradation.

To determine whether siPCSK9 enhances Aβ uptake and transport, we performed fluorescence‐based uptake assays using 5‐TAMRA–labeled Aβ_1–42_. Confocal microscopy demonstrated markedly stronger intracellular red fluorescence in the siPCSK9‐treated group compared with controls, suggesting enhanced Aβ internalization (Figure 1G and Figure S6A). Flow cytometry confirmed that Aβ uptake increased in a time‐dependent manner following siPCSK9 treatment (Figure 1H and Figure S6B). Control experiments excluded non‐specific binding (Figure S6C,D), confirming the specificity of this effect.

We then evaluated Aβ transport across the BBB using a Transwell model established with bEnd.3 cells. Transendothelial electrical resistance (TEER) values stabilized at ∼29.8 Ω·cm^2^ on day 6, forming a confluent monolayer suitable for comparative transport assays (Figure S7). siPCSK9 treatment significantly enhanced Aβ transendothelial transport from the brain parenchymal side to the blood side (B → A), but had no significant effect on transport in the opposite direction (A → B) (Figure 1I,J). This indicates that PCSK9 silencing specifically promotes clearance transport of Aβ. Importantly, ELISA‐based degradation assays revealed that while siPCSK9‐treated cells exhibited increased uptake and clearance of Aβ, the degradation rate itself remained unchanged (Figure 1K,L), suggesting that PCSK9 silencing primarily facilitates Aβ clearance by stabilizing LRP1‐mediated uptake and transport rather than accelerating degradation.

Collectively, these results demonstrate that PCSK9 upregulation in cerebrovascular endothelial cells and microglia in AD exacerbates Aβ clearance deficits through LRP1 degradation. Conversely, siPCSK9 intervention stabilizes LRP1 expression, enhances Aβ uptake and BBB transport in cerebrovascular endothelial cells, and improves microglial Aβ uptake, providing experimental support for targeting PCSK9 as a strategy to restore Aβ clearance in AD. Importantly, siPCSK9 enhanced Aβ uptake and B→A transport but did not significantly increase intracellular Aβ degradation, indicating that restoration of efflux alone may be insufficient when intracellular Aβ processing capacity is limited. This observation led us to examine whether enhanced Aβ handling creates a secondary intracellular degradation bottleneck.

### 2.2 Dual PCSK9/TRIB3 Silencing Improves Aβ Clearance by Combining Enhanced Aβ Efflux with Restored Intracellular Degradation

Having established that siPCSK9 enhances Aβ uptake and transport, we next investigated the consequences of increased intracellular Aβ burden on cellular physiology. In cerebrovascular endothelial cells, internalized Aβ is primarily degraded through the autophagy–lysosome pathway [21]. Monodansylcadaverine (MDC) staining revealed that with increasing Aβ concentrations, the number and fluorescence intensity of autophagic vesicles in bEnd.3 cells progressively increased (Figure S8A,B), indicating autophagy initiation. To assess autophagic flux, bEnd.3 cells were transfected with a pCMV‐mCherry‐GFP‐LC3B plasmid, which allows dynamic tracking of autophagosome–lysosome fusion (with GFP quenched in acidic lysosomes and mCherry stably expressed). In the Aβ‐treated group, accumulation of yellow/orange puncta (GFP^+^/mCherry^+^) indicated impaired autophagosome–lysosome fusion (Figure 2A). Notably, siPCSK9 treatment further increased yellow/orange puncta under Aβ exposure, indicating that PCSK9 silencing may aggravate autophagic flux blockade when intracellular Aβ handling burden is increased.

**FIGURE 2:**
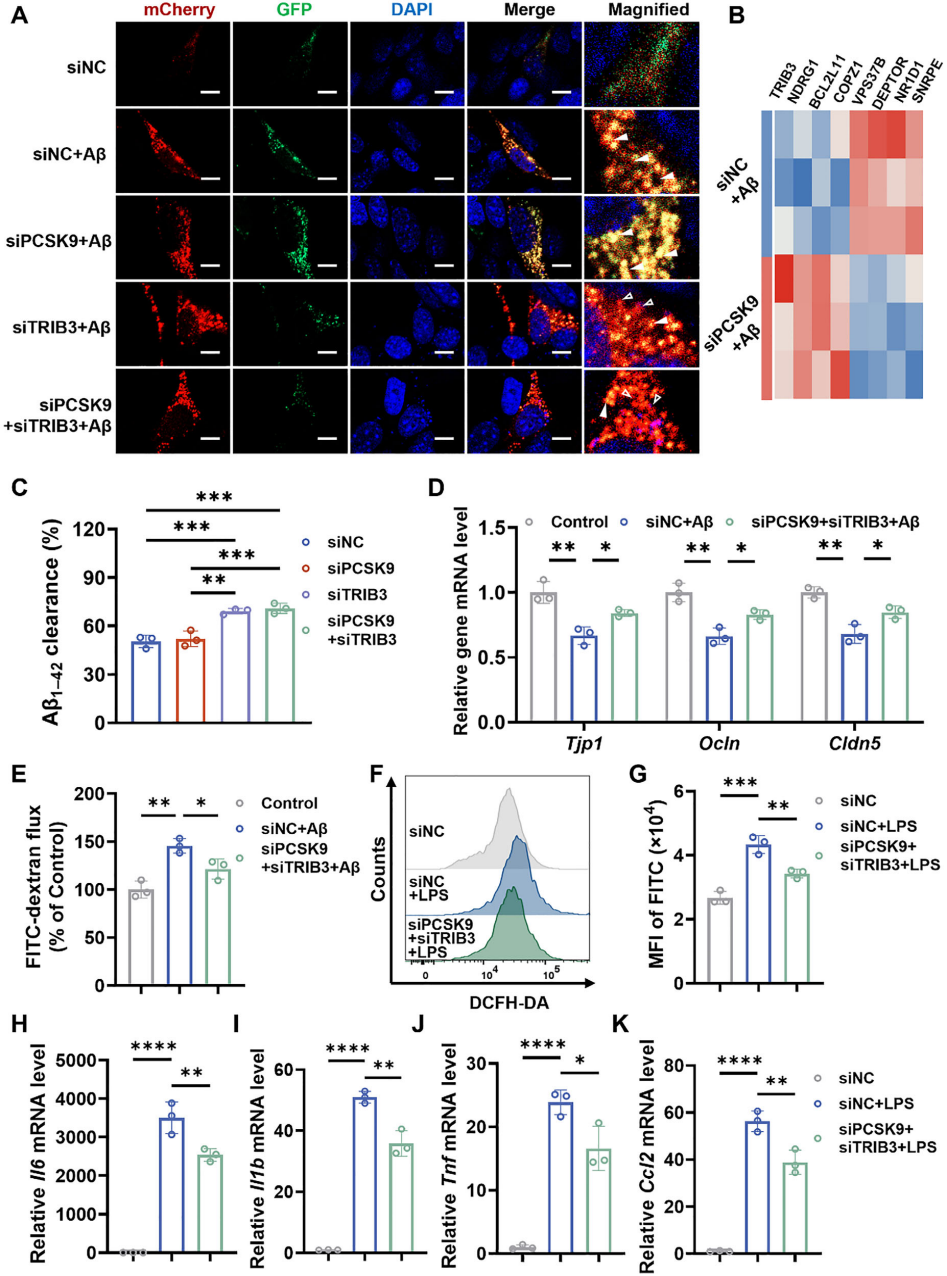
siPCSK9 enhances Aβ uptake but impairs autophagic flux; combined siTRIB3 restores autophagy, promotes Aβ degradation, and reduces inflammation. (A) Autophagic flux tracking in bEnd.3 cells transfected with pCMV‐mCherry‐GFP‐LC3B and treated with control siRNA (siNC), siPCSK9, siTRIB3, or siPCSK9+siTRIB3, followed by Aβ_1–42_ exposure. GFP (acid‐sensitive) is quenched in lysosomes, whereas mCherry is stable. Yellow/orange puncta (GFP^+^/mCherry^+^; solid triangles) correspond to autophagosomes with blocked lysosomal fusion, while red puncta (GFP^−^/mCherry^+^; hollow triangles) correspond to autolysosomes formed after successful fusion. Scale bar = 20 µm. (B) Heatmap of autophagy‐related genes, including TRIB3, across treatment groups (red = high expression; blue = low expression). (C) Intracellular Aβ_1_–_4__2_ degradation rate after treatment with siNC, siPCSK9, siTRIB3, or siPCSK9+siTRIB3. (D) Relative mRNA expression of tight junction genes (*Tjp1*, *Ocln*, *Cldn5*) following siPCSK9+siTRIB3 treatment under Aβ exposure in bEnd.3 cells. Values were normalized to the mean value of the control group for each gene, which was set to 1. (E) Transwell permeability assay of FITC‐dextran (4 kDa) across bEnd.3 monolayers treated with siPCSK9+siTRIB3 and Aβ. Values were normalized to the mean value of the control group, which was set to 100%. (F,G) ROS levels in lipopolysaccharide (LPS)‐stimulated BV2 cells after combined siPCSK9+siTRIB3 intervention, shown by representative flow cytometry histograms (F) and mean fluorescence intensity (MFI) quantification (G). (H–K) Relative mRNA expression of *Il6* (H), *Il1b* (I), *Tnf* (J), and *Ccl2* (K) in BV2 cells under LPS stimulation with combined intervention. Data are presented as mean ± SD (*n* = 3 independent experiments). One‐way ANOVA followed by Tukey's multiple‐comparison test for (C, E, and G–K); two‐way ANOVA followed by Tukey's multiple‐comparison test for (D). ^*^ *p* < 0.05, ^**^ *p* < 0.01, ^***^ *p* < 0.001, ^****^ *p* < 0.0001.

Given that intracellular Aβ overload can induce oxidative stress and cell death [38, 39, 40], we assessed reactive oxygen species (ROS) production and cytotoxicity. DCFH‐DA staining showed that siPCSK9‐transfected bEnd.3 cells exposed to Aβ displayed significantly elevated ROS levels (Figure S8C), and Calcein/PI double staining confirmed increased cytotoxicity (Figure S8D). These results indicate that although siPCSK9 enhances Aβ uptake and transport, it may simultaneously cause autophagic dysfunction and oxidative stress injury due to insufficient Aβ degradation.

To identify the molecular mediator associated with this autophagic blockade, we analyzed autophagy‐related transcriptomic changes in cerebrovascular endothelial cells. RNA sequencing revealed significant upregulation of TRIB3 following siPCSK9 + Aβ treatment (Figure 2B and Figure S9A,B), which was further validated by RT‐qPCR in bEnd.3 cells (Figure S9C). To determine whether PCSK9 silencing directly induces TRIB3 under basal conditions, we performed TRIB3 immunofluorescence staining in bEnd.3 cells treated with siNC or siPCSK9 in the absence of exogenous Aβ. siPCSK9 alone did not significantly alter TRIB3 fluorescence intensity compared with siNC (Figure S9D,E). Together with the TRIB3 upregulation observed under siPCSK9 + Aβ conditions, these results suggest that TRIB3 induction is unlikely to be caused by PCSK9 silencing alone. Rather, TRIB3 appears to be induced as a secondary stress response under increased Aβ‐loading conditions after restoration of LRP1‐dependent Aβ uptake/transport by PCSK9 silencing.

To determine whether TRIB3 is pathologically altered in cerebrovascular endothelial cells under AD‐like conditions, we performed co‐immunofluorescence staining of TRIB3 with the endothelial marker CD31 in brain sections from 6‐month‐old APP/PS1 mice and age‐matched wild‐type (WT) littermate controls. Compared with WT mice, APP/PS1 mice showed markedly increased TRIB3 fluorescence in CD31^+^ cerebrovascular regions (Figure S9F,G), indicating elevated endothelial‐associated TRIB3 signal in the AD mouse brain vasculature in vivo.

Building on previous studies demonstrating that Aβ‐induced TRIB3 overexpression blocks neuronal autophagy [41], we hypothesized that TRIB3 contributes to the impaired autophagic flux observed after siPCSK9 intervention under increased Aβ‐handling burden. To test this, a TRIB3‐specific siRNA (siTRIB3‐3) was designed and validated (Figure S10A). Under Aβ exposure, siTRIB3 reduced yellow/orange puncta (GFP^+^/mCherry^+^) and increased red‐only puncta (GFP^−^/mCherry^+^) compared with siPCSK9 treatment, indicating restoration of autophagosome–lysosome fusion. Combined siPCSK9+siTRIB3 intervention similarly improved autophagic flux (Figure 2A).

To define the functional step regulated by TRIB3, we compared Aβ_1–42_ transendothelial transport and intracellular Aβ_1_–_4__2_ degradation after siNC, siPCSK9, siTRIB3, or siPCSK9+siTRIB3 treatment. In the Transwell BBB model, siPCSK9 enhanced B→A Aβ transport, consistent with restored LRP1‐dependent efflux, whereas siTRIB3 alone did not significantly affect either B→A or A→B Aβ transport. The combined siPCSK9+siTRIB3 group showed a B→A transport effect mainly comparable to siPCSK9 alone (Figure S10B,C). In contrast, siTRIB3 significantly promoted intracellular Aβ_1_–_4__2_ degradation, whereas siPCSK9 alone had only a limited effect. Combined siPCSK9+siTRIB3 treatment also improved Aβ degradation, with an effect mainly attributable to TRIB3 silencing (Figure 2C). This degradation‐enhancing effect was suppressed by the late‐stage autophagy inhibitor hydroxychloroquine (HCQ), but not by the early‐stage inhibitor 3‐methyladenine (3‐MA), indicating dependence on restored late autophagic flux (Figure S10D). These results indicate that TRIB3 primarily regulates intracellular Aβ degradation rather than directional transendothelial Aβ efflux.

We next investigated the autophagy‐related molecular basis underlying this TRIB3‐dependent degradation effect. Immunofluorescence analysis demonstrated that siTRIB3 reduced p62 accumulation (Figure S11A–C) and increased PPARα expression (Figure S11D–F). Pearson correlation coefficients confirmed co‐localization of TRIB3 with p62 (r = 0.74) and PPARα (r = 0.88), consistent with its regulatory role in late‐stage autophagy [32, 33, 34]. The effect of siTRIB3 on Aβ degradation may be related, at least in part, to restoration of PPARα‐associated lysosomal biogenesis, lysosomal homeostasis and lysosome‐dependent autophagy, as supported by the observed effects of late‐stage autophagy inhibitor HCQ [42, 43].

We next evaluated endothelial barrier integrity. Aβ exposure significantly downregulated the mRNA expression of tight junction–associated markers, including zonula occludens‐1 (ZO‐1), occludin, and claudin‐5, whereas combined siPCSK9 and siTRIB3 treatment restored their expression (Figure 2D). Transwell permeability assays further demonstrated that combined intervention reduced FITC‐dextran (4 kDa) leakage (Figure 2E) and mitigated Aβ‐induced cytotoxicity (Figure S12). These findings suggest that combined PCSK9/TRIB3 silencing alleviates Aβ‐induced endothelial barrier disruption, potentially associated with autophagy‐related preservation of tight junction integrity [44].

Given the role of autophagy in modulating microglial inflammatory activation [27, 45], we next assessed inflammatory responses using a LPS‐stimulated BV2 microglial model. Combined siPCSK9 and siTRIB3 treatment significantly decreased ROS levels (Figure 2F,G) and nitric oxide (NO) secretion (Figure S13A), and reduced mRNA expression of *Il6*, *Il1b*, *Tnf*, and *Ccl2* (Figure 2H–K). Flow cytometry revealed a decrease in M1‐like CD80^+^/CD86^+^ microglia, alongside an increase in M2‐like CD206^+^ cells (Figure S13B–G), consistent with a phenotypic shift toward an anti‐inflammatory‐like state.

Taken together, these findings indicate that PCSK9 and TRIB3 regulate distinct but complementary aspects of Aβ clearance. PCSK9 silencing mainly restores LRP1‐dependent Aβ uptake and B→A transendothelial efflux, whereas TRIB3 silencing mainly promotes intracellular Aβ degradation by relieving autophagy–lysosomal blockade. Therefore, combined PCSK9/TRIB3 silencing improves Aβ clearance by coupling enhanced efflux with restored intracellular degradation, while also preserving endothelial barrier integrity and suppressing inflammatory activation. Considering the challenges of siRNA instability and delivery barriers in vivo, we next developed a brain‐enriched nanodelivery system for simultaneous delivery of siPCSK9 and siTRIB3.

### 2.3 Construction and Characterization of Brain‐Enriched SITR for Co‐Delivery of siPCSK9 and siTRIB3

To enable simultaneous brain‐enriched delivery of siPCSK9 and siTRIB3, we developed a RAGE‐targeted nanocarrier system, termed SITR, designed to integrate “dual‐gene synergy” with “brain‐enriched delivery” (Scheme 1B). We first optimized siRNA loading efficiency in TPNs. FAM‐labeled siRNA at concentrations of 2, 4, and 8 µm was encapsulated, and the resulting formulations were assessed. As siRNA concentration increased, the zeta potential of SIT gradually decreased (−18.6 to −21.6 mV), while the polydispersity index (PDI) remained below 0.2, indicating good colloidal stability (Figure 3A, Table S1 and Figure S14A). Although siRNA payload increased with higher loading concentrations, encapsulation efficiency decreased accordingly (Figure 3B). Considering both loading efficiency and nanoparticle stability, a total siRNA concentration of 4 µm (siPCSK9:siTRIB3 = 1:1) was selected for subsequent studies.

**FIGURE 3:**
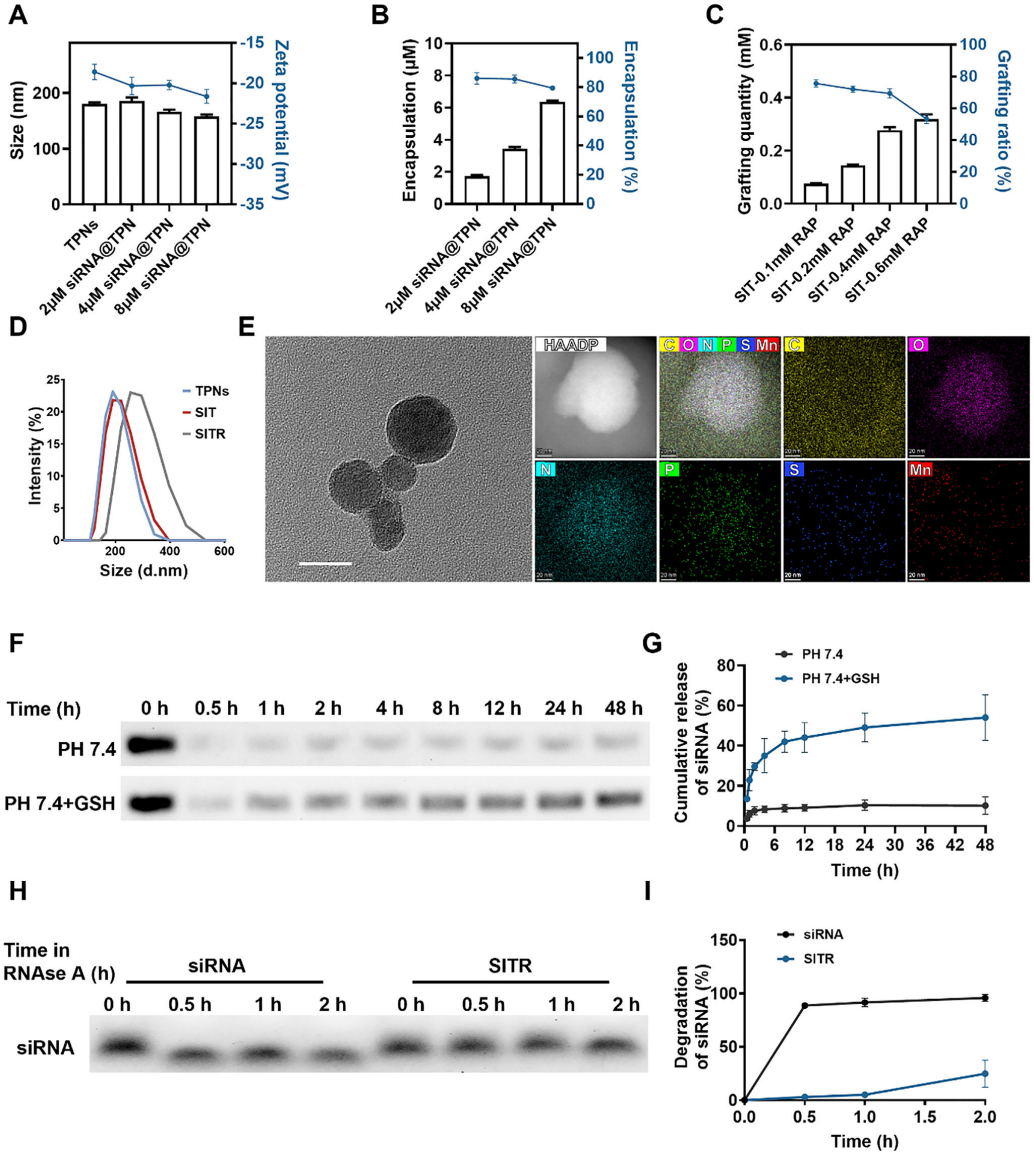
Construction and physicochemical characterization of SITR nanocarriers. (A) Hydrodynamic size distribution (left) and zeta potential (right) of SIT prepared with different siRNA concentrations (2, 4, 8 µm). (B) Quantitative analysis of siRNA payload (left) and encapsulation efficiency (right) at varying loading concentrations. (C) RAP grafting amount (left) and GR (right) at different RAP feed concentrations (0.1–0.6 mm). (D) Hydrodynamic size distribution of TPNs, SIT, and SITR. (E) TEM image (scale bar = 100 nm, left) and energy‐dispersive elemental mapping (scale bar = 20 nm, right) of SITR, showing C, O, N, P, S, and Mn signals. (F,G) In vitro siRNA release profiles from SITR in PBS (pH 7.4) or PBS containing 10 mm GSH for 48 h, shown by agarose gel electrophoresis (F) and cumulative release analysis (G). (H,I) RNase A protection assay of SITR, shown by gel electrophoresis (H) and quantitative degradation analysis (I). Data are presented as mean ± SD (*n* = 3 independently prepared nanoparticle batches).

To enhance brain enrichment, we exploited the elevated expression of RAGE in AD brain vasculature and activated microglia [15, 46, 47]. RAP, an S100P‐derived RAGE‐antagonistic peptide capable of binding RAGE, was grafted onto SIT through SH‐PEG_2__000_‐RAP conjugation to confer RAGE‐guided brain‐enrichment capability [48, 49]. By varying RAP feed concentrations (0.1–0.6 mm), we observed a dose‐dependent increase in particle size and grafting rate (GR). Notably, no significant improvement in RAP grafting was achieved beyond 0.4 mm, which was therefore selected as the optimal modification condition (Figure 3C and Figure S14B).

The physicochemical properties of optimized SIT and SITR are summarized in Figures 3D,E and S14C–E. SITR exhibited a hydrated particle size of 256.6 ± 6.7 nm, PDI of 0.13 ± 0.01, and a zeta potential of −23.6 ± 0.7 mV. The siRNA encapsulation efficiency was 78.22% ± 1.07%, while RAP GR reached 69.41% ± 2.04% (0.28 ± 0.01 mm). Transmission electron microscopy (TEM) revealed irregular spherical morphology with diameters of 100–200 nm, and energy dispersive spectroscopy confirmed the presence of C and O (from TPNs), P (siRNA), S (RAP), N (siRNA and RAP), and Mn (catalyst residue), verifying successful siRNA encapsulation and RAP conjugation (Figure 3E).

SIT and SITR displayed good colloidal stability, with no significant particle size changes after 48 h incubation in HEPES (pH 7.4), PBS, saline, or serum‐containing Dulbecco's Modified Eagle's Medium (DMEM) (Figure S14F,G). In vitro release studies demonstrated glutathione (GSH)‐responsive behavior: under extracellular mimic conditions (PBS, pH 7.4), cumulative siRNA release was limited to 10.19 ± 3.63% over 48 h, whereas under intracellular reducing conditions (PBS + 10 mm GSH), SITR exhibited rapid initial release within 2 h followed by sustained release, achieving 54.04% ± 9.32% cumulative release at 48 h (Figure 3F,G).

Stability assays further confirmed that SITR effectively protected siRNA against nuclease degradation. Free siRNA was almost completely degraded within 2 h in RNase A solution (96% degradation), whereas SITR encapsulation limited degradation to ∼25% under the same conditions (Figure 3H,I). Similarly, free siRNA degraded rapidly in 10% fetal bovine serum (FBS) within 24 h, while SITR‐encapsulated siRNA remained intact (Figure S14H).

Together, these results confirm that SITR possesses high encapsulation efficiency, favorable stability, GSH‐responsive intracellular release, and effective nuclease protection, providing a robust platform for systemic siRNA delivery in vivo.

### 2.4 RAGE‐Mediated Targeted Uptake, Endosomal/Lysosomal Escape, and BBB Penetration of SITR

To assess the targeting and internalization capacity of SITR in activated cerebrovascular endothelial cells and microglia, FAM‐labeled siRNA was employed for intracellular visualization, with DAPI counterstaining for nuclear localization. Fluorescence microscopy demonstrated markedly stronger green signals in Aβ‐activated cells treated with SITR compared with free siRNA or non‐targeted SIT (Figure 4A,B), indicating enhanced uptake. Pre‐incubation with excess free RAP peptide substantially reduced intracellular fluorescence, confirming that SITR entry was primarily mediated by RAP–RAGE interactions. Flow cytometry quantification corroborated the imaging results: SITR showed significantly higher uptake in activated cells than other groups, with a clear time‐dependent increase (2–8 h), which was attenuated by RAGE blocking (Figure 4C,D).

**FIGURE 4:**
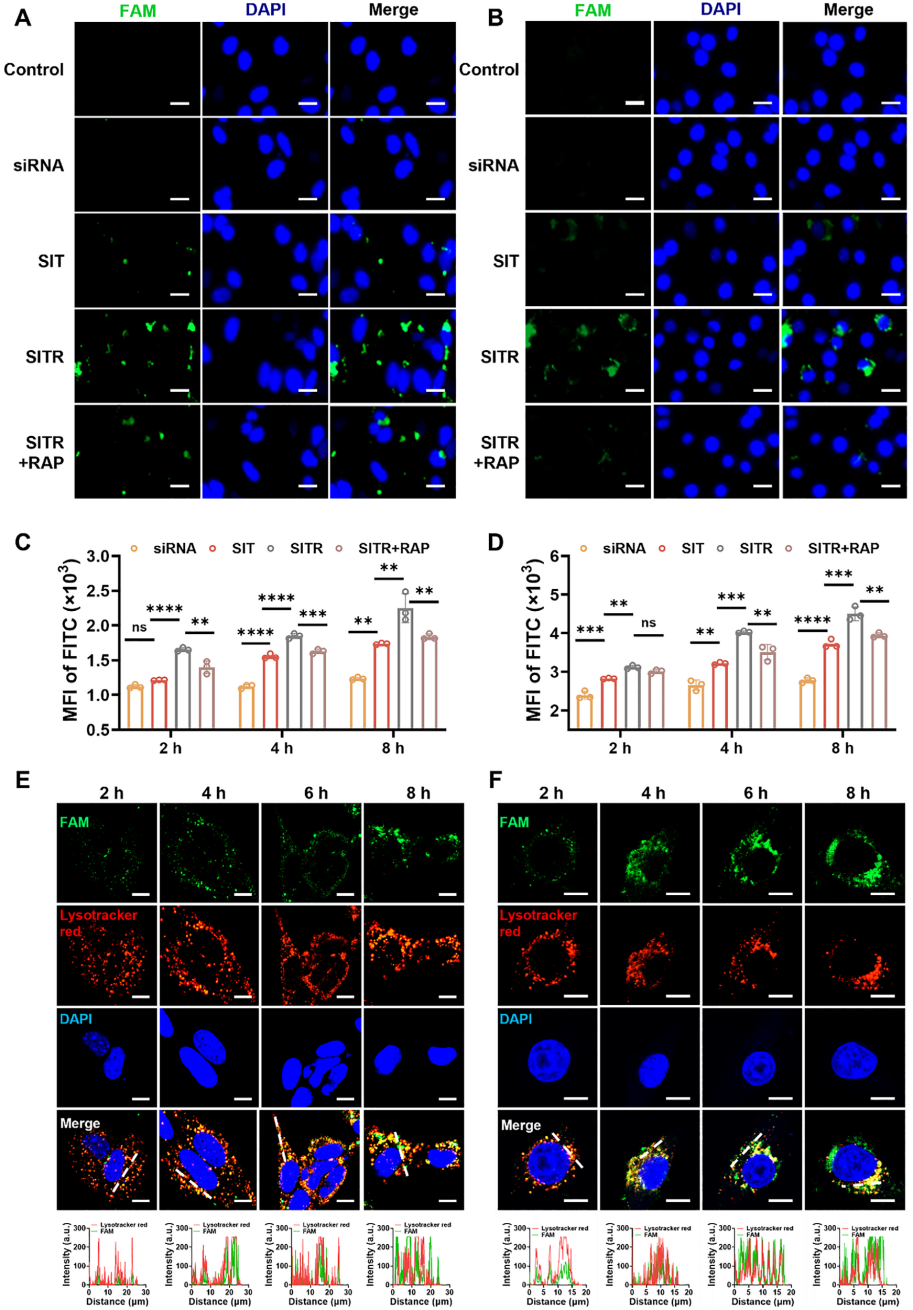
Targeted uptake, endosomal/lysosomal escape, and BBB transport capacity of SITR. (A and B) Confocal fluorescence images of FAM‐siRNA (green) in Aβ‐pretreated bEnd.3 cells (A) and BV2 cells (B) after treatment with free siRNA, SIT, SITR, or SITR plus excess RAP peptide. Nuclei were stained with DAPI (blue). Scale bar = 20 µm. (C,D) Flow cytometry quantification of cellular uptake in Aβ‐pretreated bEnd.3 cells (C) and BV2 cells (D) at 2, 4, and 8 h after treatment. (E,F) Lysosomal colocalization assays in bEnd.3 cells (E) and BV2 cells (F). Confocal images show FAM‐siRNA (green), LysoTracker Red (red), and nuclei (blue, DAPI) at 2, 4, 6, and 8 h after incubation. Fluorescence intensity distribution plots along dashed lines show time‐dependent endosomal/lysosomal escape. Scale bar = 10 µm. Data are presented as mean ± SD (*n* = 3 independent experiments). One‐way ANOVA followed by Tukey's multiple‐comparison test at each time point for (C,D). ns, not significant; ^*^ *p* < 0.05, ^**^ *p* < 0.01, ^***^ *p* < 0.001, ^****^ *p* < 0.0001.

Following cellular internalization, endosomal/lysosomal escape is essential for siRNA bioavailability. Confocal colocalization assays revealed strong overlap of SITR (green, FAM‐siRNA) with LysoTracker Red in bEnd.3 (Figure 4E) and BV2 cells (Figure 4F) within the first 2–4 h, yielding an orange merged signal. At 6–8 h, the separation of green and red signals indicated progressive endosomal/lysosomal escape of SITR. This phenomenon can be attributed to the “proton sponge” effect of the nanocarrier under acidic lysosomal conditions, facilitating siRNA release into the cytoplasm to enable gene silencing.

To determine whether SITR facilitates BBB penetration, an in vitro BBB model based on bEnd.3 monolayers was established. Different formulations (free siRNA, SIT, SITR) were added to the upper chamber, and fluorescence intensity in the lower chamber was quantified to calculate transcytosis efficiency. SITR exhibited markedly enhanced transendothelial transport, reaching 4.78% ± 0.19% at 12 h, compared with SIT (2.71% ± 0.20%) and free siRNA (0.93% ± 0.10%) (Figure S15).

These findings demonstrate that RAP modification not only promotes SITR recognition and internalization by RAGE‐overexpressing cerebrovascular endothelial cells, but also significantly improves BBB‐crossing capability, thereby enabling enhanced delivery of therapeutic siRNA to brain lesions.

### 2.5 SITR Enhances Aβ Clearance, Restores Autophagic Function, and Synergistically Protects Endothelial Barrier Integrity in Cerebrovascular Endothelial Cells

Having established the targeted uptake capacity of SITR, we next investigated its intracellular biological effects in cerebrovascular endothelial cells. Specifically, we examined its ability to regulate Aβ metabolism, restore autophagic flux, and protect endothelial barrier function.

Cytotoxicity was first assessed using CCK‐8 assays. Across bEnd.3, BV2, and HT22 cells, treatment with TPNs, SIT, or SITR at siRNA‐equivalent concentrations ≤ 200 nm maintained >80% viability, while viability at 50 nm exceeded 90% (Figure S16A–C). Based on these results, 50 nm was selected as the working concentration for subsequent in vitro experiments.

Flow cytometry demonstrated that SITR significantly increased Aβ uptake by bEnd.3 cells compared with other groups (Figure 5A). Consistently, in a Transwell model, SITR markedly enhanced transendothelial transport of Aβ from the basolateral (brain) to the apical (blood) side (B→A), but did not alter Aβ influx from the apical to basolateral side (A→B) (Figure 5B,C). Western blotting further revealed that SITR upregulated LRP1 expression in bEnd.3 cells (Figure 5D,E), suggesting that SITR promotes Aβ clearance across the BBB by stabilizing LRP1.

**FIGURE 5:**
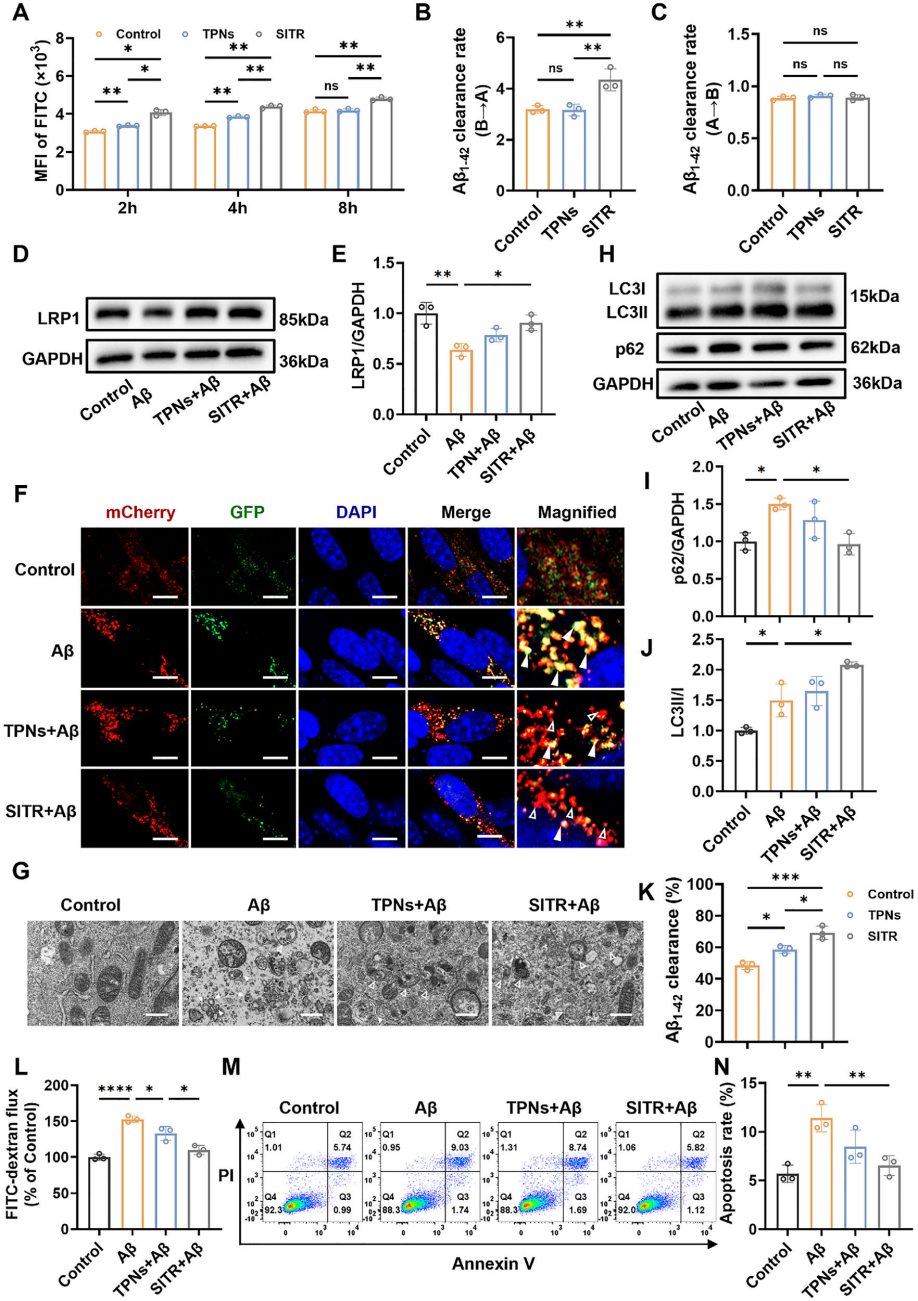
SITR enhances Aβ clearance, restores autophagy, and protects endothelial barrier integrity in bEnd.3 cells. (A) Flow cytometry quantification of 5‐TAMRA‐Aβ_1__−__4__2_ uptake at 37°C in bEnd.3 cells treated with different formulations. (B,C) Transwell transport assays showing Aβ_1__−__4__2_ clearance transport from basolateral (B, brain) to apical (A, blood) side (B→A) (B), and Aβ_1__−__4__2_ influx from A→B direction (C). (D,E) WB analysis of LRP1 expression: representative blots (D) and densitometric quantification (E) Values were normalized to the mean value of the control group, which was set to 1. (F) Confocal images of bEnd.3 cells transfected with mCherry‐GFP‐LC3B and treated with Aβ plus different formulations. Yellow/orange puncta (solid triangles) indicate autophagosomes; red puncta (hollow triangles) indicate autolysosomes. Scale bar = 10 µm. (G) TEM images showing autophagic structures. Solid triangles denote autophagosomes; hollow triangles denote autolysosomes. Scale bar = 500 nm. (H–J) WB analysis of LC3II, LC3I, and p62: representative blots (H) and quantitative analysis (I,J). Values were normalized to the mean value of the control group, which was set to 1. (K) Quantitative analysis of intracellular Aβ_1__−__4__2_ degradation rate in bEnd.3 cells. (L) FITC‐dextran permeability assay evaluating endothelial barrier integrity. Values were normalized to the mean value of the control group, which was set to 100%. (M,N) Annexin V/PI apoptosis analysis: representative scatter plots (M) and quantitative statistics (N). Data are presented as mean ± SD (*n* = 3 independent experiments). Two‐way ANOVA followed by Tukey's multiple‐comparison test for (A); one‐way ANOVA followed by Tukey's multiple‐comparison test for (B, C, E, I–L, and N). ns, not significant; ^*^ *p* < 0.05, ^**^ *p* < 0.01, ^***^ *p* < 0.001, ^****^ *p* < 0.0001.

Autophagy function was then evaluated. The mCherry‐GFP‐LC3B dual‐fluorescence assay indicated that Aβ exposure caused accumulation of yellow/orange puncta (autophagosomes), reflecting autophagic flux blockade. SITR treatment decreased autophagosome accumulation and increased red‐only puncta (autolysosomes), indicating restoration of autophagic degradation (Figure 5F). TEM imaging corroborated these findings, showing greater numbers of autolysosomes in the SITR group (Figure 5G). Western blotting confirmed increased LC3II/I ratio and reduced p62 expression after SITR treatment (Figure 5H–J). In line with these changes, ELISA showed that SITR significantly enhanced intracellular degradation of Aβ (Figure 5K).

Oxidative stress analysis revealed that Aβ markedly elevated intracellular ROS levels, while SITR significantly suppressed ROS accumulation beyond the modest antioxidant effect of TPNs (Figure S17A,B). Endothelial barrier assays demonstrated that SITR substantially reduced Aβ‐induced FITC‐dextran leakage across bEnd.3 monolayers, outperforming TPNs (Figure 5L).

Finally, SITR alleviated Aβ‐induced cytotoxicity. Calcein‐AM/PI staining showed a reduced proportion of dead cells following SITR treatment (Figure S17C). Annexin V/PI assays confirmed that SITR significantly decreased Aβ‐induced apoptosis (Figure 5M,N). CCK‐8 assays also demonstrated restoration of cell viability (Figure S17D).

Collectively, these results demonstrate that SITR exerts a multifaceted protective effect in cerebrovascular endothelial cells: it promotes Aβ clearance via LRP1 stabilization, restores autophagic flux to enhance Aβ degradation, attenuates oxidative stress and apoptosis, and preserves endothelial barrier integrity. Together, these synergistic effects provide a mechanistic basis for SITR‐mediated protection of the neurovascular unit.

### 2.6 SITR Enhances Aβ Clearance in Microglia and Regulates Inflammation and Phenotypic Polarization

In the pathological context of AD, Aβ activates microglial phagocytosis, but excessive activation often triggers oxidative stress and inflammatory cascades, thereby aggravating neuronal injury. Considering that TRIB3 downregulation enhances autophagy‐mediated degradation and that TPNs carriers possess intrinsic anti‐inflammatory properties [50, 51, 52, 53], we further investigated the regulatory effects of SITR on microglial function.

Flow cytometry revealed that SITR markedly enhanced the phagocytic uptake of Aβ by BV2 microglia (Figure 6A), consistent with findings in cerebrovascular endothelial cells. Autophagic flux tracing using the mCherry‐GFP‐LC3B reporter showed that SITR effectively alleviated Aβ‐induced autophagy blockade and promoted autophagosome–lysosome fusion (Figure 6B). Correspondingly, ELISA demonstrated that SITR significantly increased intracellular Aβ degradation within 24 h compared with other groups (Figure 6C). Calcein‐AM/PI staining further indicated that SITR reduced Aβ‐induced microglial cell death more effectively than TPNs (Figure 6D).

**FIGURE 6:**
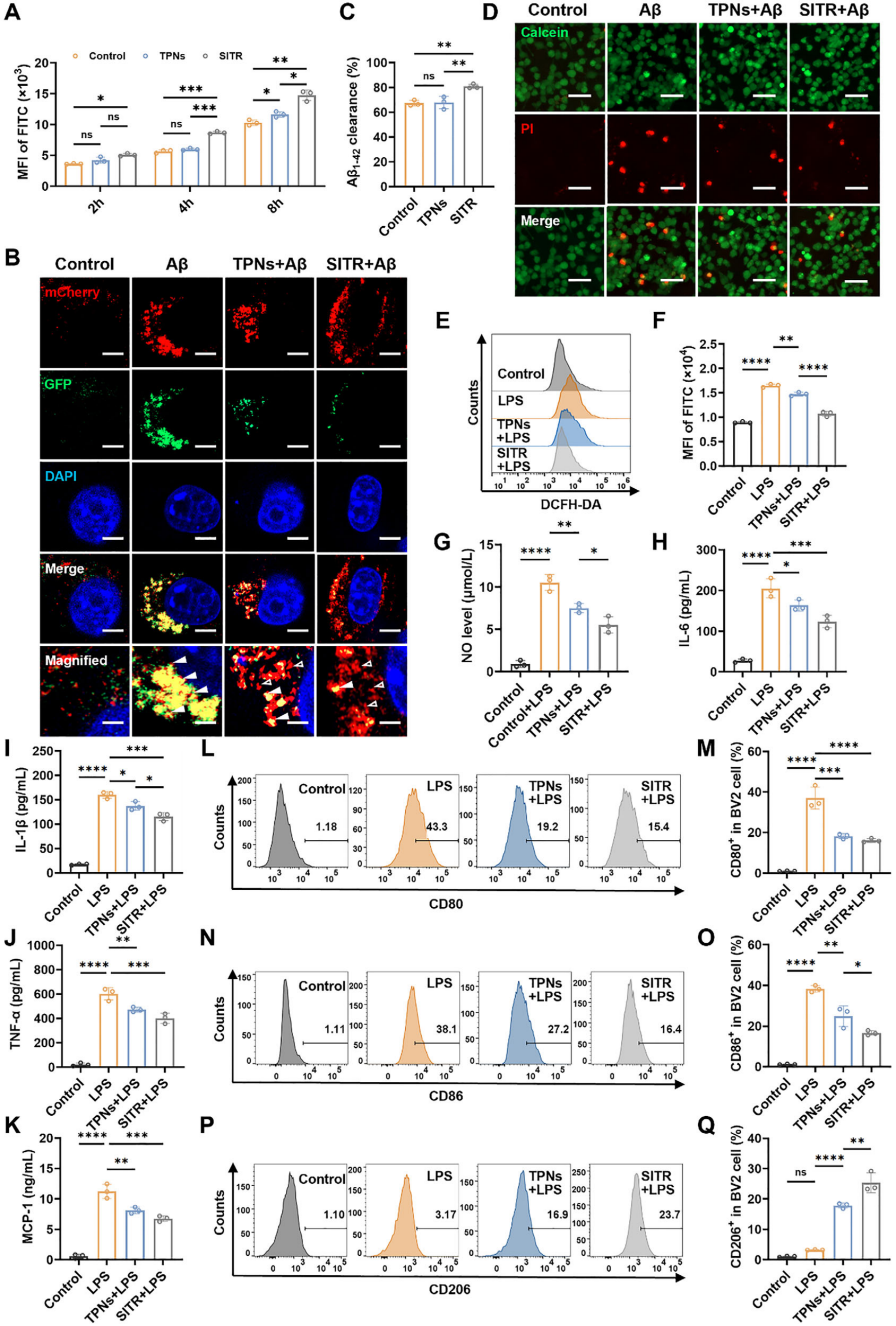
SITR promotes Aβ clearance, alleviates inflammation, and regulates microglial polarization. (A) Flow cytometry quantification of 5‐TAMRA‐Aβ_1__−__4__2_ uptake at 37°C in BV2 cells treated with different formulations. (B) Confocal images of BV2 cells transfected with mCherry‐GFP‐LC3B and treated with Aβ plus different formulations. Yellow/orange puncta (solid triangles) represent autophagosomes; red puncta (hollow triangles) represent autolysosomes. Scale bar = 10 µm. (C) Quantitative analysis of intracellular Aβ degradation rate after different treatments. (D) Calcein‐AM/PI staining images of BV2 cells after Aβ and different treatments (green: live cells; red: dead cells). Scale bar = 50 µm. (E,F) Intracellular ROS analysis in LPS‐stimulated BV2 cells: representative flow cytometry plots (E) and MFI quantification (F). (G) Quantification of NO levels in cell supernatants after LPS stimulation and treatment. (H–K) ELISA quantification of pro‐inflammatory cytokines IL‐6 (H), IL‐1β (I), TNF‐α (J), and MCP‐1 (K) after LPS stimulation and treatment. (L–Q) Flow cytometry analysis of microglial polarization: representative plots and quantification of CD80^+^ (L,M), CD86^+^ (N,O), and CD206^+^ (P,Q) populations under different conditions. Data are presented as mean ± SD (*n* = 3 independent experiments). Two‐way ANOVA followed by Tukey's multiple‐comparison test for (A); one‐way ANOVA followed by Tukey's multiple‐comparison test for (C, F–Q). ns, not significant; ^*^ *p* < 0.05, ^**^ *p* < 0.01, ^***^ *p* < 0.001, ^****^ *p* < 0.0001.

To assess anti‐inflammatory and antioxidant functions, an LPS‐stimulated BV2 inflammation model was employed. SITR significantly decreased intracellular ROS accumulation, as quantified by DCFH‐DA fluorescence, surpassing the effect of TPNs (Figure 6E,F). Measurement of supernatant NO confirmed reduced levels following SITR treatment (Figure 6G). ELISA analysis revealed that SITR significantly suppressed LPS‐induced secretion of pro‐inflammatory cytokines, including IL‐6, IL‐1β, TNF‐α, and MCP‐1, with stronger inhibition than TPNs (Figure 6H–K).

Given the central role of microglial polarization in AD neuroinflammation, we further examined phenotypic transformation. Flow cytometry analysis showed that LPS stimulation increased expression of M1‐like markers (CD80, CD86) and reduced the M2‐like marker CD206. Both TPNs and SITR reversed these changes, while SITR exhibited the most pronounced effect, suppressing CD80^+^ and CD86^+^ populations and enhancing CD206^+^ cells (Figure 6L–Q).

Taken together, SITR not only augments Aβ uptake and degradation in microglia but also attenuates oxidative stress, inhibits pro‐inflammatory mediator release, and promotes repolarization from the detrimental M1‐like phenotype toward the protective M2‐like phenotype. These multifaceted effects highlight the potential of SITR in reshaping the neuroinflammatory microenvironment in AD.

### 2.7 RAGE‐Targeted Brain‐Enriched Delivery and Systemic Biodistribution of SITR in AD Mice

Following confirmation of SITR's biological activity in vitro, in vivo experiments were conducted to evaluate its brain‐enrichment efficiency. Hemolysis assays demonstrated that within the concentration range of 0–800 µg/mL, the hemolysis rates of TPNs, SIT, and SITR remained below 5% (Figure S18A–C), indicating that the formulations were biocompatible and suitable for intravenous administration.

To assess in vivo biodistribution and brain‐enriched delivery, Cy5.5‐labeled siRNA was used as a fluorescent tracer and monitored by longitudinal in vivo fluorescence imaging. In APP/PS1 transgenic mice, SITR administration produced markedly stronger Cy5.5‐associated fluorescence in the cranial region than free siRNA and non‐RAP‐modified SIT (Figure 7A). Quantification of Cy5.5‐associated average radiant efficiency revealed that the cranial signal in the SITR group peaked at 8 h post‐injection and remained elevated up to 24 h (Figure 7B), supporting enhanced brain‐enriched delivery and apparent retention of siRNA‐associated fluorescence.

**FIGURE 7:**
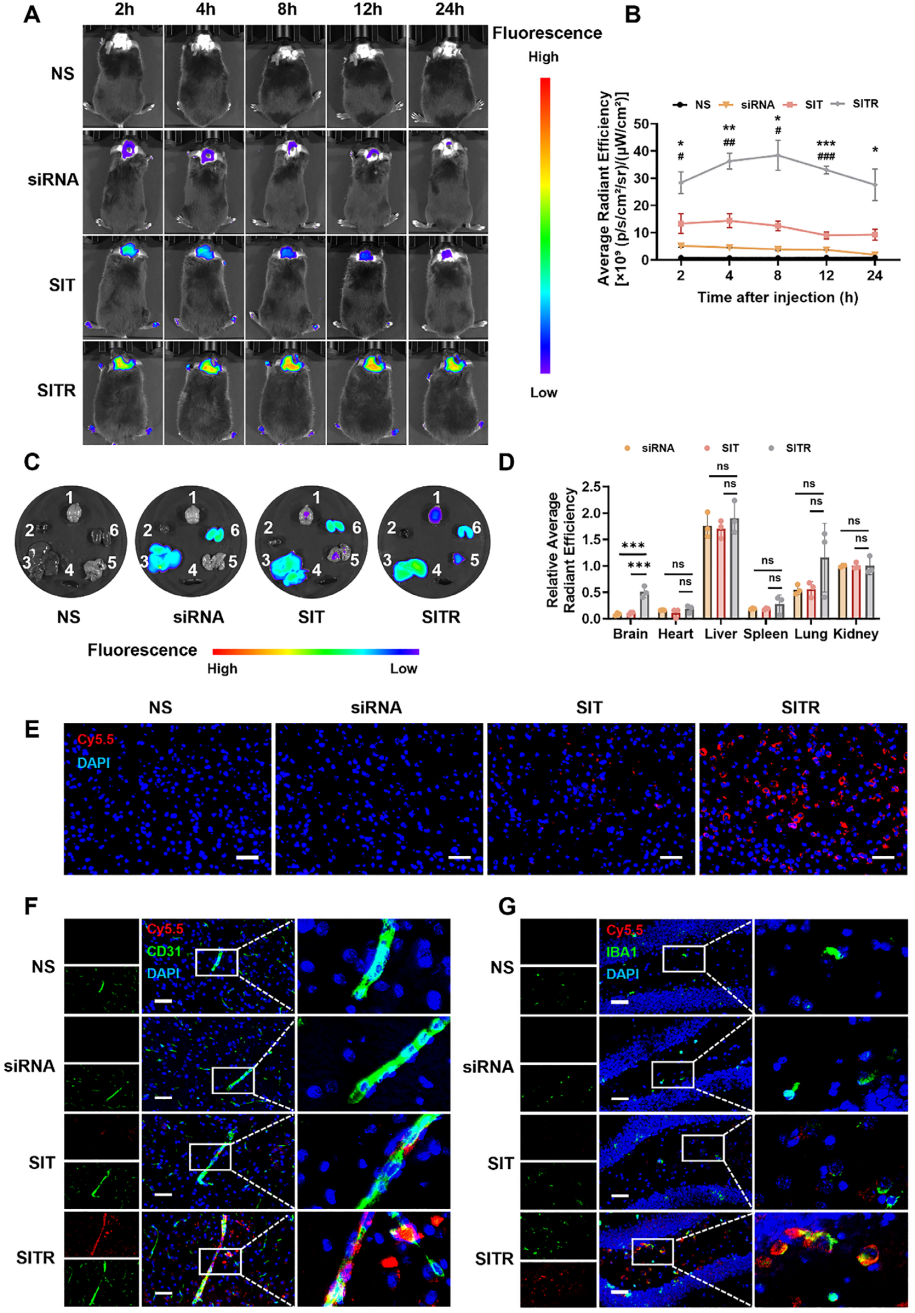
RAGE‐targeted brain‐enriched delivery and systemic biodistribution of SITR in APP/PS1 mice. (A) Representative longitudinal in vivo fluorescence imaging of APP/PS1 mice after intravenous injection of Cy5.5‐siRNA, Cy5.5‐SIT, or Cy5.5‐SITR at different time points. (B) Quantification of average radiant efficiency in the cranial region over time. (C) Ex vivo fluorescence imaging of major organs and brain tissues at 24 h post‐injection (1: brain, 2: heart, 3: liver, 4: spleen, 5: lungs, 6: kidneys). (D) Relative quantification of average radiant efficiency in the brain and major organs collected at 24 h post‐injection. Values were normalized to the mean value of the kidneys within the corresponding treatment group, which was set to 1. (E) Representative fluorescence images of brain tissue sections showing the distribution of Cy5.5‐labeled formulations at 24 h after administration. Scale bar = 50 µm. (F) Immunofluorescence images showing co‐localization of Cy5.5‐labeled siRNA‐associated signals (red) with CD31^+^ cerebrovascular endothelial cells (green) in cortical tissue (DAPI, blue nuclei). Scale bar = 100 µm. (G) Representative immunofluorescence images showing co‐localization of Cy5.5‐labeled siRNA‐associated signals (red) with IBA1^+^ microglia (green) in hippocampal tissue (DAPI, blue nuclei). Scale bar = 50 µm. Data are presented as mean ± SD (*n* = 3 mice per group). Two‐way ANOVA followed by Tukey's multiple‐comparisons test for (B), and one‐way ANOVA followed by Tukey's multiple‐comparison test among treatment groups for each organ in (D). For (B), ^*^ *p* < 0.05, ^**^ *p* < 0.01 and ^***^ *p* < 0.001 indicate SITR versus free siRNA at the same time point; and #*p* < 0.05, ##*p* < 0.01, ###*p* < 0.001 indicate SITR versus SIT at the same time point. For (D), ns, not significant; ^*^ *p* < 0.05, ^**^ *p* < 0.01, ^***^ *p* < 0.001, ^****^ *p* < 0.0001.

Ex vivo fluorescence imaging of Cy5.5‐labeled siRNA‐associated major organs and brain at 24 h post‐injection further confirmed the brain‐enriched distribution of SITR (Figure 7C). Relative quantification of average radiant efficiency showed that SITR produced a significantly higher brain fluorescence signal than free siRNA or SIT; the kidney‐normalized brain signal in the SITR group was approximately 5.85‐fold higher than that in the free siRNA group (Figure 7C,D). These results are consistent with RAP/RAGE‐associated brain enrichment. Notably, prominent fluorescence signals were also detected in the liver and kidneys, indicating peripheral distribution and clearance rather than brain‐specific accumulation (Figure 7C,D). Therefore, SITR is described as a brain‐enriched, rather than brain‐specific, delivery system. This peripheral exposure was further considered in the subsequent metabolic and systemic safety analyses.

Fluorescence analysis of frozen brain sections corroborated these findings, showing substantially higher Cy5.5 signal in the SITR group compared with free siRNA and SIT (Figure 7E). Immunofluorescence co‐staining further demonstrated preferential deposition in CD31^+^ cerebrovascular endothelial cells (Figure 7F) and IBA1^+^ microglia (Figure 7G), indicating that RAP modification improves targeting toward AD‐relevant neurovascular and immune cell populations in the brain.

Collectively, these results indicate that RAP modification enables SITR to efficiently cross the BBB via RAGE‐mediated transport, preferentially accumulating in cerebrovascular endothelial cells and microglia. This targeted delivery lays the foundation for subsequent Aβ clearance and anti‐neuroinflammatory activity in AD models.

### 2.8 Improvement of Cognitive Function and Attenuation of Pathological Damage in AD Mice by SITR

After confirming the in vivo brain‐targeting properties of SITR, we next evaluated its therapeutic efficacy in APP/PS1 transgenic mice. Six‐month‐old AD model mice were randomly divided into groups receiving NS, TPNs, SIT, or SITR, while age‐matched WT littermates served as normal controls (NS only). Each group received weekly intravenous injections for six consecutive weeks, followed by behavioral and pathological assessments (Figure 8A).

**FIGURE 8:**
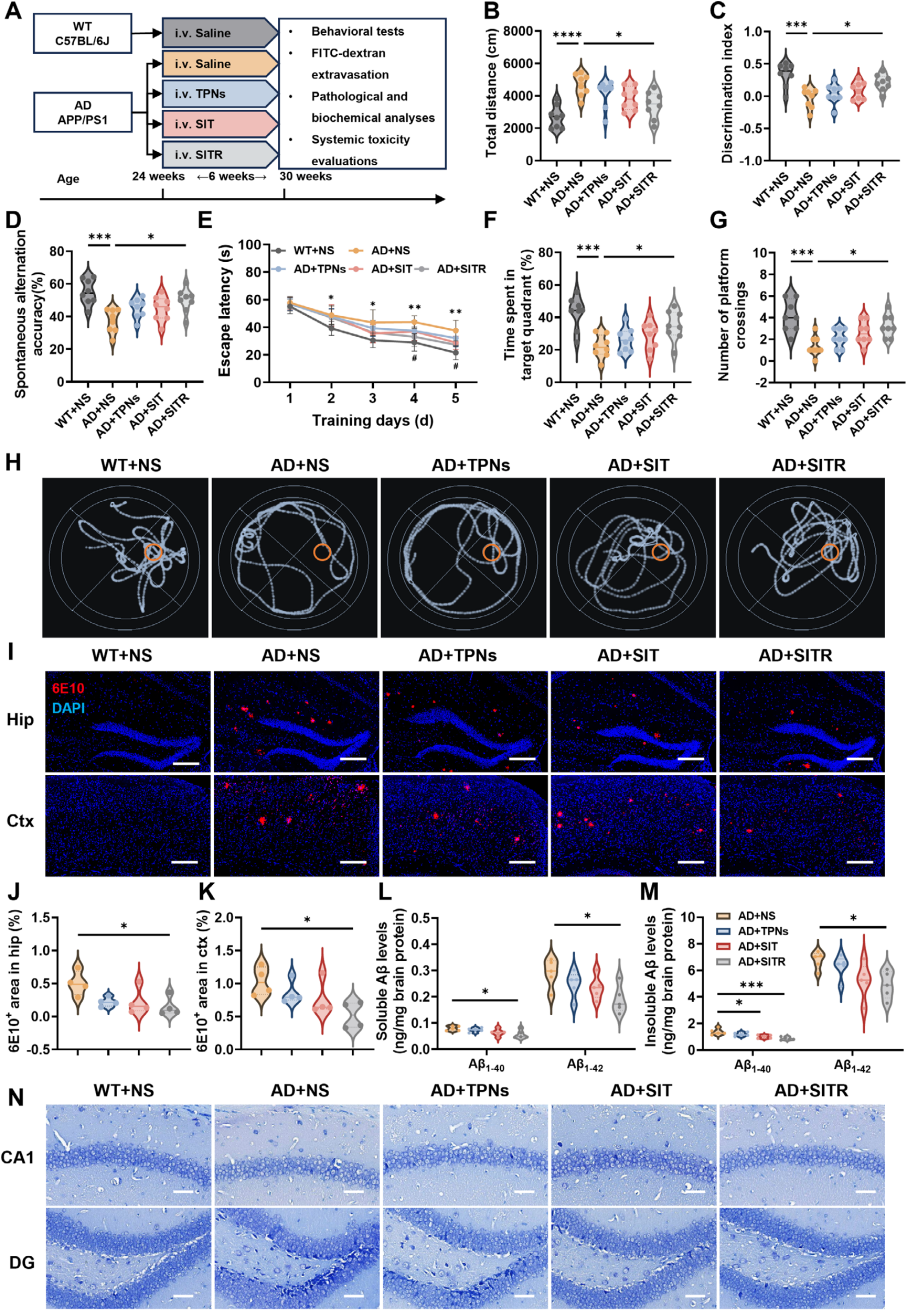
SITR improves cognitive function and alleviates pathological damage in APP/PS1 mice. (A) Schematic of the in vivo experimental design. Six‐month‐old APP/PS1 mice received NS, TPNs, SIT, or SITR by intravenous tail vein injection once weekly for six consecutive weeks; WT littermates received NS (*n* = 8–9 mice per group). Behavioral tests were performed after treatment. Following behavioral tests, mice were deeply anesthetized and euthanized by transcardial perfusion for subsequent pathological, biochemical, and systemic toxicity evaluations. (B) Open field test: total movement distance and central exploration distance (*n* = 8 mice per group). (C) Novel object recognition (NOR) test: discrimination index for novel object preference (*n* = 8 mice per group). (D) Y‐maze test: spontaneous alternation percentage (*n* = 8 mice per group). (E) Morris water maze (MWM): escape latency during acquisition training on days 1–5 (*n* = 8 mice per group). (F) Probe trial of MWM on day 6: percentage of time spent in the target quadrant (*n* = 8 mice per group). (G) Probe trial of MWM on day 6: number of platform crossings (*n* = 8 mice per group). (H) Representative swimming trajectories in the probe trial of MWM. (I) Representative immunofluorescence images of Aβ plaques (6E10 antibody) in hippocampus (hip) and cortex (ctx). Scale bar = 200 µm. (J and K) Quantification of Aβ plaque burden in hippocampus (hip) (J) and cortex (ctx) (K). (*n* = 4 mice per group). (L,M) ELISA quantification of soluble (Tris‐soluble) (L) and insoluble (guanidine‐soluble) (M) Aβ_1__−__4__0_ and Aβ_1__−__4__2_ in cortical tissue (*n* = 5 mice per group). (N) Representative Nissl staining images of hippocampal CA1 and DG regions. Scale bar = 50 µm. One‐way ANOVA followed by Tukey's multiple‐comparison test for (B–D, F, G, J, and K) and separately among treatment groups for Aβ_1_–_4__0_ and Aβ_1_–_4__2_ in (L,M); repeated‐measures two‐way ANOVA followed by Tukey's multiple‐comparison test for (E). For (B–D, F, G, L, M, J, and K), ^*^ *p* < 0.05, ^**^ *p* < 0.01, ^***^ *p* < 0.001, ^****^ *p* < 0.0001. For (E), ^*^ *p* < 0.05 and ^**^ *p* < 0.01 indicate AD+NS versus WT+NS, and #*p* < 0.05 indicates AD+SITR versus AD+NS.

In the open field test, AD model mice exhibited significantly increased total movement distance compared with WT controls, indicative of hyperactivity and behavioral disinhibition. SITR treatment significantly reduced locomotor hyperactivity (Figure 8B and Figure S19A,B). In the novel object recognition (NOR) test, AD mice failed to discriminate between novel and familiar objects, with discrimination indices approaching zero. SITR treatment significantly restored recognition memory, as reflected by increased discrimination indices (Figure 8C). Similarly, in the Y‐maze test, AD mice showed reduced spontaneous alternation, while SITR significantly improved this parameter, suggesting recovery of working memory and discrimination learning (Figure 8D).

Spatial learning and memory were further evaluated using the Morris water maze (MWM) (Figure 8E–H). AD mice exhibited prolonged escape latencies during the learning phase, and decreased target quadrant dwell time and platform crossings during the probe test. SITR significantly shortened escape latencies (Figure 8E) and increased both target quadrant dwell time and platform crossings (Figure 8F,G). Representative swimming trajectories further confirmed improved spatial memory performance (Figure 8H). No differences in swimming speed or total swimming distance were observed among groups (Figure S19C,D), excluding motor impairment as a confounding factor. Collectively, these results demonstrate that SITR enhances both short‐term working memory and long‐term spatial memory in AD mice.

Histopathological analyses corroborated the behavioral findings. Immunofluorescence staining with anti‐6E10 antibody revealed abundant Aβ plaque deposition in the cortex and hippocampus of AD mice, which was markedly reduced by SITR treatment (Figure 8I–K). ELISA further confirmed significantly reduced levels of both soluble and insoluble fractions of Aβ_1__−__4__0_ and Aβ_1__−__4__2_ in SITR‐treated brains compared with those of untreated AD mice (Figure 8L,M).

Nissl staining showed decreased and irregularly distributed Nissl bodies in the hippocampal cornu ammonis 1 (CA1) and dentate gyrus (DG) of AD mice, whereas SITR treatment restored Nissl body density and distribution, indicating improved neuronal structural integrity (Figure 8N).

Taken together, SITR exhibited superior therapeutic efficacy compared with SIT and TPNs, significantly improving behavioral performance, reducing Aβ burden, and alleviating neuronal damage in AD mice. These results systematically validate the advantages of targeted siRNA delivery for AD intervention.

### 2.9 Mechanisms of SITR‐Mediated Aβ Clearance, BBB Protection, and Neuroinflammation Attenuation

To elucidate the mechanisms underlying SITR's therapeutic effects, we conducted multidimensional analyses in APP/PS1 mice.

First, the gene‐silencing capacity of SITR was validated. Reverse Transcription Quantitative Real‐Time Polymerase Chain Reaction (RT‐qPCR) analyses demonstrated that SITR effectively downregulated the expression of *Pcsk9* and *Trib3* in brain tissue, while simultaneously upregulating *Lrp1*  (Figure 9A–C). Consistently, ELISA quantification revealed significantly elevated plasma levels of Aβ_1__−__4__2_ and a rising trend in Aβ_1__−__4__0_ in SITR‐treated mice compared with untreated AD controls, suggesting enhanced Aβ clearance across the BBB via LRP1‐mediated transport (Figure 9D).

**FIGURE 9:**
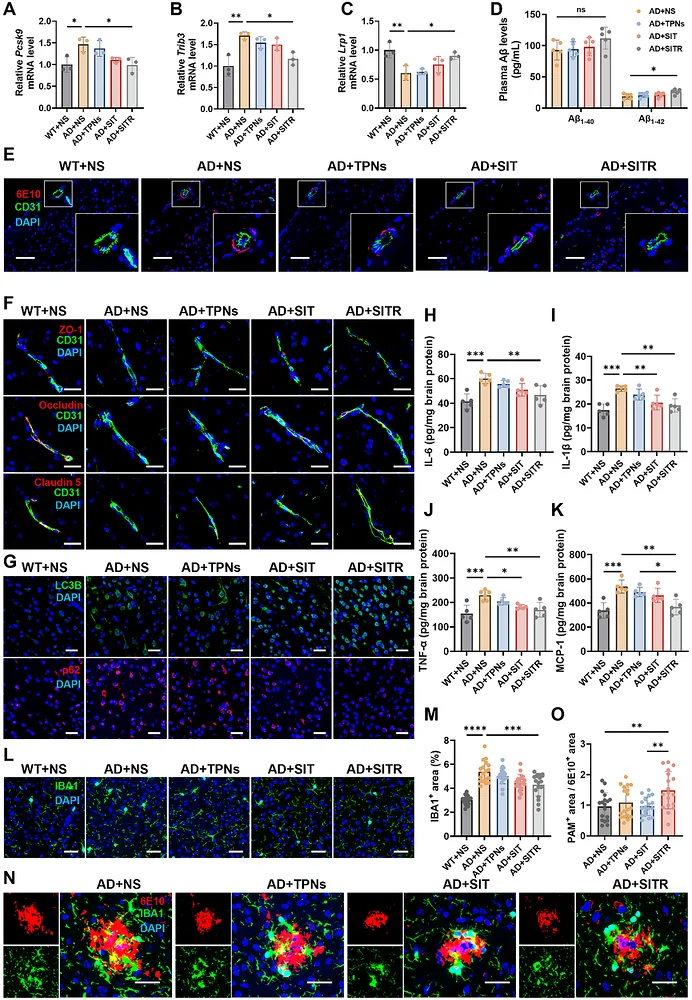
Mechanistic studies of SITR in promoting Aβ clearance, protecting BBB integrity, and alleviating neuroinflammation in APP/PS1 mice. (A to C) Relative mRNA expression of *Pcsk9* (A), *Trib3* (B), and *Lrp1* (C) in cortical tissues (*n* = 3 mice per group). Values were normalized to the mean value of the WT+NS group, which was set to 1. (D) ELISA quantification of plasma Aβ_1__−__4__0_ and Aβ_1__−__4__2_ levels (*n* = 5 mice per group). (E) Representative immunofluorescence images of co‐localization of CD31 (endothelial marker, green) and Aβ (6E10 antibody, red) showing vascular Aβ deposition. Scale bar = 200 µm. (F) Representative images of CD31 co‐localization with tight junction proteins ZO‐1, occludin, and claudin‐5. Scale bar = 25 µm. (G) Immunofluorescence images of autophagy markers LC3B and p62 in cortical tissue. Scale bar = 25 µm. (H–K) ELISA quantification of pro‐inflammatory cytokines IL‐6 (H), IL‐1β (I), TNF‐α (J), and MCP‐1 (K) in cortical tissue (*n* = 5 mice per group). (L–M) Representative immunofluorescence images (L) and quantification (M) of IBA1^+^ microglia. Scale bar = 25 µm; *n* = 3 mice per group, six measurements were taken from each mouse. (N,O) Co‐localization of IBA1 (microglial marker, green) with Aβ (6E10, red) (N); Quantification of plaque‐associated microglia (PAMs) and intracellular Aβ burden (O). Scale bar = 25 µm; *n* = 3 mice per group, six measurements were taken from each mouse. Data are presented as mean ± SD. One‐way ANOVA followed by Tukey's multiple‐comparison test for (A‐C, H‐K, M, and O) and separately among treatment groups for Aβ_1_–_4__0_ and Aβ_1_–_4__2_ in (D). ns, not significant; ^*^ *p* < 0.05, ^**^ *p* < 0.01, ^***^ *p* < 0.001, ^****^ *p* < 0.0001.

We next assessed CAA, a frequent AD complication characterized by vascular Aβ deposition [6, 10]. Double immunofluorescence staining of Aβ (6E10) and CD31 demonstrated that SITR reduced Aβ deposition around cerebral vessels (Figure 9E), indicating alleviated CAA pathology.

To evaluate BBB integrity, we analyzed the expression of endothelial tight junction proteins. Immunofluorescence revealed that SITR significantly restored the levels of ZO‐1, occludin, and claudin‐5 in cortical vasculature (Figure 9F and Figure S20, A to C). FITC‐dextran (40 kDa) leakage assays confirmed reduced BBB permeability in SITR‐treated mice (Figure S21). Furthermore, SITR activated endothelial autophagy, as evidenced by increased LC3B signal and reduced p62 expression (Figure 9G and Figure S22A,B), indicating restoration of autophagic flux and structural maintenance of the BBB.

The anti‐inflammatory effects of SITR were also examined. ELISA revealed that pro‐inflammatory cytokines (IL‐6, IL‐1β, TNF‐α, and MCP‐1) were significantly elevated in AD mice but markedly suppressed following SITR treatment (Figure 9H–K). Immunofluorescence analysis showed a reduction in IBA1^+^ microglial density in SITR‐treated brains (Figure 9L,M). Importantly, co‐localization of IBA1 with Aβ plaques revealed an increase in plaque‐associated microglia (PAMs) after SITR administration, along with reduced intracellular Aβ load per microglial cell (Figure 9N,O). These findings indicate that SITR enhances microglial phagocytosis and degradation of Aβ, thereby facilitating its clearance from brain tissue.

These in vivo findings connect the cellular mechanisms identified in vitro with therapeutic outcomes in APP/PS1 mice. In summary, SITR promotes Aβ clearance and attenuates AD pathology through synergistic mechanisms, including target gene silencing, upregulation of LRP1‐mediated efflux, alleviation of CAA pathology, restoration of BBB integrity via autophagy regulation, and suppression of neuroinflammation.

### 2.10 Systematic Safety Evaluation of SITR in APP/PS1 Mice

To assess the in vivo biosafety of SITR, a systematic safety evaluation was conducted in APP/PS1 mice after six consecutive weeks of treatment. Throughout the treatment period, no significant differences in body weight were observed between groups (Figure S23), indicating that SITR administration did not overtly affect overall growth or systemic health.

We first evaluated serum biochemical parameters and organ‐injury markers. Compared with the AD+NS group, SITR treatment did not induce significant adverse changes in liver‐related parameters, including aspartate aminotransferase (AST), alanine aminotransferase (ALT), alkaline phosphatase (ALP), and total protein (TP) (Figure S24A–D). Renal function‐related parameters, including blood urea nitrogen (BUN), creatinine (CRE), and uric acid (UA), also showed no significant pathological elevation after SITR treatment (Figure S24E–G). In addition, serum cardiac injury‐related markers, including cardiac troponin I (cTnI), creatine kinase‐MB (CK‐MB), and lactate dehydrogenase (LDH), were not abnormally elevated (Figure S24H–J). These data provide no biochemical evidence of overt liver, kidney, or myocardial injury under the current treatment regimen.

Hematological analysis was performed to evaluate potential blood toxicity or systemic inflammatory abnormalities. White blood cells (WBC), red blood cells (RBC), hemoglobin (HGB), and platelets (PLT) showed no significant adverse changes after SITR treatment compared with control groups (Figure S24K–N). These findings do not suggest obvious hematological toxicity within the tested groups and observation period.

Given that ex vivo biodistribution analysis revealed substantial liver and kidney fluorescence signals, we further evaluated its peripheral impacts on glucose and lipid metabolism, considering the roles of PCSK9 and TRIB3 in LDL‐C regulation and insulin signaling, respectively [54, 55]. Fasting glucose (GLU), serum insulin, and the homeostatic model assessment of insulin resistance (HOMA‐IR) were not significantly altered in SITR‐treated mice (Figure S25A–C), suggesting no obvious disturbance of glucose metabolism or insulin sensitivity. Serum lipid profiling showed that total cholesterol (TCHO) and triglycerides (TG) exhibited a mild decreasing trend in the SIT‐ and SITR‐treated groups. Low‐density lipoprotein cholesterol (LDL‐C) was significantly reduced after SIT and SITR treatment, whereas high‐density lipoprotein cholesterol (HDL‐C) remained largely unchanged (Figure S25D–G). These lipid changes are consistent with a PCSK9‐related lipid‐modulatory effect [54, 56].

Finally, histopathological evaluation of major organs, including the heart, liver, spleen, lungs, and kidneys, was performed using hematoxylin and eosin (H&E) staining. Representative H&E images did not show obvious treatment‐related morphological abnormalities in SITR‐treated mice (Figure S26A). Semi‐quantitative histopathological scoring across all groups showed only occasional minimal histological findings in major organs. No significant differences in histopathological scores were observed among groups in the heart, liver, spleen, lungs, or kidneys (Figure S26B–F). These results provide no clear histopathological evidence of SITR‐associated short‐term tissue toxicity under the current treatment regimen.

Collectively, within the parameters and duration of the current study, no clear biochemical, hematological, or histopathological indications of tissue toxicity were observed, indicating that SITR treatment was well tolerated over the 6‐week regimen. However, the observed liver/kidney fluorescence signals and LDL‐C reduction indicate that peripheral exposure and peripheral PCSK9‐related pharmacodynamic effects cannot be fully excluded. Therefore, long‐term metabolic safety, peripheral target engagement, and dose‐dependent off‐tissue effects require further investigation.

### 2.11 Integrated Discussion and Limitations

The present study establishes a dual‐target Aβ clearance strategy that coordinates restoration of transendothelial efflux with enhancement of intracellular autophagic degradation. Unlike approaches that primarily reduce Aβ production, inhibit Aβ aggregation, or passively remove extracellular plaques, SITR is designed to correct two sequential clearance barriers. PCSK9 silencing stabilizes LRP1 and restores Aβ uptake and efflux across the neurovascular interface, whereas TRIB3 silencing relieves an intracellular autophagy–lysosomal degradation bottleneck and promotes Aβ degradation. Importantly, PCSK9 and TRIB3 should not be interpreted as unrelated or redundant targets. Instead, they act as functionally complementary regulators at distinct steps of Aβ clearance: PCSK9 mainly controls the LRP1‐dependent uptake/efflux arm, while TRIB3 limits the intracellular degradative arm under increased Aβ‐loading conditions. Consistent with this interpretation, PCSK9 silencing alone does not markedly alter basal TRIB3 expression in the absence of exogenous Aβ, whereas TRIB3 is upregulated as a downstream consequence of the increased intracellular Aβ‐handling burden caused by PCSK9 silencing. These findings support functional coupling within the Aβ clearance pathway, rather than a direct biochemical interaction between PCSK9 and TRIB3.

This framework may help explain why single‐pathway interventions have shown limited therapeutic effects in AD [57]. Enhancing Aβ uptake or efflux alone may increase intracellular Aβ‐handling burden if autophagic degradation remains impaired [38, 39, 40]. Conversely, stimulating intracellular degradation without restoring BBB efflux may be insufficient to reduce vascular and parenchymal Aβ accumulation [58]. By combining these two mechanisms, SITR reduces Aβ deposition, CAA, BBB disruption, and neuroinflammation in APP/PS1 mice. The reduction of vascular Aβ deposition is particularly relevant because cerebrovascular dysfunction and CAA contribute to cognitive decline and may complicate anti‐Aβ antibody therapies [7, 10, 12].

The microglial effects of SITR further support a broader neuroimmune mechanism. SITR enhanced microglial Aβ uptake and degradation, reduced ROS and NO production, suppressed IL‐6, IL‐1β, TNF‐α, and MCP‐1 release, and shifted microglia toward a CD206^+^ M2‐like phenotype. These anti‐inflammatory effects may be partly explained by the restoration of autophagy‐dependent intracellular proteostasis in microglia [59, 60]. Autophagy contributes to microglial Aβ degradation and negatively regulates Aβ‐induced NLRP3 inflammasome activation, linking defective autophagy to chronic neuroinflammation [61]. Consistently, autophagy has also been implicated in the regulation of microglial polarization and inflammatory responses [27, 60].

The autophagy–inflammation axis may also intersect with cellular senescence. Recent studies have shown that microglial autophagy supports amyloid plaque engagement and helps prevent senescence‐associated microglial states [62]. Conversely, senescent or senescence‐like glial cells may amplify chronic neuroinflammation through senescence‐associated secretory phenotype (SASP)‐like cytokines and chemokines, thereby contributing to AD progression [63]. In the present study, SITR reduces oxidative stress, pro‐inflammatory cytokine production, and M1‐like microglial activation, raising the possibility that it may attenuate senescence‐associated inflammatory remodeling. However, cellular senescence markers, including p16, p21, senescence‐associated β‐galactosidase, γH2AX, and Lamin B1, were not directly assessed in the present study [64]. Therefore, whether TRIB3 silencing directly modulates microglial senescence remains unresolved. Future studies should examine microglial senescence markers and NLRP3 inflammasome activity in AD models.

The systemic biodistribution and metabolic findings require careful interpretation. Although RAP modification enhances brain accumulation and increases SITR‐associated signals in CD31^+^ cerebrovascular endothelial cells and IBA1^+^ microglia, ex vivo fluorescence imaging also revealed substantial liver and kidney signals. Therefore, SITR should be considered a brain‐enriched rather than brain‐exclusive delivery system. Systemically, PCSK9 is a key regulator of cholesterol metabolism, and PCSK9 inhibition is an established strategy for lowering circulating LDL‐C [54, 56]. Accordingly, the LDL‐C reduction observed in SIT‐ and SITR‐treated mice is consistent with a peripheral PCSK9‐related pharmacodynamic effect [54, 56]. This change is not necessarily a toxicity signal, because clinically approved PCSK9‐targeting antibodies, including evolocumab and alirocumab, systemically reduce LDL‐C and have shown acceptable tolerability in large cardiovascular outcome studies [65, 66, 67]. In the FOURIER trial, evolocumab reduced LDL‐C and cardiovascular events without a significant increase in overall adverse events, including new‐onset diabetes or neurocognitive events [65]. Long‐term safety analyses from ODYSSEY OUTCOMES similarly support the tolerability of alirocumab, with local injection‐site reactions being the main excess adverse event [67]. Recent large‐scale meta‐analyses also support the cognitive safety of lipid‐lowering therapies, although their cognitive benefit in AD or cognitive decline remains debated [68, 69]. Nevertheless, the therapeutic goal of SITR differs from lipid‐lowering PCSK9 antibodies: SITR is intended to enhance brain Aβ clearance rather than to treat dyslipidemia. Moreover, although dyslipidemia has been implicated as a vascular‐metabolic risk factor for AD [70, 71, 72], the present study is not designed to evaluate lipid‐driven AD pathology and is not performed in a hyperlipidemic AD model. Thus, LDL‐C reduction should be interpreted as an expected peripheral pharmacodynamic consequence of PCSK9 silencing rather than as an independent mechanism underlying the anti‐AD efficacy of SITR. Peripheral exposure, lipid modulation, and possible TRIB3‐related metabolic effects require further evaluation during translational development, particularly in long‐term studies under dyslipidemic conditions.

Several limitations should be acknowledged. First, therapeutic efficacy is evaluated only in male APP/PS1 mice, an amyloid‐dominant model that does not fully recapitulate tau pathology, sex‐specific responses, or the clinical heterogeneity of AD; validation in additional AD models and human‐derived samples is therefore needed. Second, the current study primarily assesses a 6‐week treatment regimen, and long‐term safety, immunogenicity, repeated‐dose tolerability, and reversibility of peripheral metabolic effects remain to be determined. Third, although RAP/RAGE modification enhances brain enrichment, SITR is not brain‐exclusive, as liver and kidney exposure and LDL‐C reduction are observed; peripheral target engagement and off‐tissue effects require further evaluation. Finally, the data support pathway‐level functional coupling between PCSK9‐mediated Aβ uptake/efflux and TRIB3‐associated autophagic stress, but direct PCSK9–TRIB3 biochemical interaction, microglial senescence, and NLRP3 inflammasome activity were not directly examined.

Despite these limitations, the current findings provide proof‐of‐concept that coordinated correction of BBB efflux impairment and intracellular autophagic degradation blockade can enhance Aβ clearance and protect the neurovascular unit. This dual‐mechanism strategy may complement existing Aβ‐directed therapies and provide a framework for developing next‐generation multi‐target nanotherapeutics for AD.

## 3 Conclusion

In this study, we identified PCSK9–LRP1 dysregulation and TRIB3‐associated autophagic blockade as two functionally connected barriers to Aβ clearance in AD. PCSK9 silencing restored LRP1‐dependent Aβ uptake and transendothelial efflux, whereas TRIB3 silencing relieved the intracellular autophagy–lysosomal degradation bottleneck and promoted Aβ degradation. Based on this mechanism, we developed SITR, a RAP‐modified tea‐polyphenol nanocarrier for brain‐enriched co‐delivery of siPCSK9 and siTRIB3. In APP/PS1 mice, SITR improved cognitive performance, reduced Aβ and CAA burden, preserved BBB and neuronal integrity, restored autophagy, and suppressed neuroinflammation. Under the tested 6‐week dosing regimen, SITR appears to be well tolerated based on the measured parameters, although liver/kidney exposure and LDL‐C reduction indicate that long‐term metabolic safety and peripheral target engagement require further investigation. Collectively, our work establishes PCSK9 and TRIB3 as complementary therapeutic targets in AD and validates SITR as an effective nanoplatform that integrates “enhanced Aβ efflux + restored autophagic degradation” into a synergistic strategy for disease modification. This dual‐target approach may provide a promising framework for the next generation of AD therapeutics and represents a step toward overcoming the limitations of single‐pathway interventions.

## 4 Experimental Section

### 4.1 Materials

Three distinct siRNAs against PCSK9 (siPCSK9‐1, siPCSK9‐2, siPCSK9‐3) and three against TRIB3 (siTRIB3‐1, siTRIB3‐2, siTRIB3‐3), along with a negative siNC, were designed and synthesized by Sangon Biotech (Shanghai, China), with their specific sequences detailed in Table S2. FAM‐ and Cy5.5‐labeled siRNAs for tracking experiments were procured from Youkang Biotechnology (Hangzhou, China), and transfection was performed using GMTrans Liposomal Transfection Reagent from Jiman Biotechnology (Shanghai, China). Recombinant human Aβ_1–42_ monomers, both unlabeled and labeled with 5‐TAMRA, as well as the RAP peptide (SH‐PEG_2000_‐ELKVLMEKEL), were sourced from Qiangyao Biotechnology (Shanghai, China). For immunological and cellular assays, flow cytometry antibodies, including PE‐conjugated anti‐mouse CD80 antibody (clone: 16‐10A1, #104707), PE/Cyanine7‐conjugated anti‐mouse CD86 antibody (clone: GL‐1, #105014), and PE/Dazzle 594‐conjugated anti‐mouse CD206 antibody (clone: C068C2, #141731), were obtained from Biolegend (San Diego, CA, USA), and an Annexin V‐Alexa Fluor 647/PI apoptosis assay kit was obtained from 4A Biotech (Beijing, China). A suite of detection reagents was sourced from Beyotime Biotechnology (Shanghai, China), including DAPI staining solution, LysoTracker Red, FITC‐labeled dextrans (4 kDa and 40 kDa), the mCherry‐GFP‐LC3B reporter plasmid (pCMV‐mCherry‐GFP‐LC3B), ROS Assay Kit (DCFH‐DA), NO Assay Kit (Griess reagent method), Calcein AM/PI Cell Double‐Staining Kit, Autophagy Staining Assay Kit (MDC method), and a Total Thiol Assay Kit (DTNB method). Cytokine and metabolic profiling was conducted using ELISA kits for human Aβ_1–40_  (JL41245) and Aβ_1–42_ (JL41255) as well as for mouse IL‐6 (JL20268), IL‐1β (JL18442), TNF‐α (JL10484), MCP‐1 (JL20304), cTnI (JL11280), and insulin (JL11459) from Shanghai Jianglai Industrial Limited By Share Ltd (Shanghai, China), in addition to clinical biochemistry assay kits from Yichuanbio (Guangzhou, China) for assessing AST, ALT, ALP, TP, CRE, BUN, UA, TCHO, TG, LDL‐C, HDL‐C, and GLU levels. Key small molecules and biochemicals included epigallocatechin gallate (EGCG, ≥98% purity) from Huagao Bioproducts Co. (Chengdu, China); FITC‐inulin, 3‐MA, HCQ, manganese chloride tetrahydrate (MnCl_2_·4H_2_O), GSH, and crystal violet from Aladdin Biochemical Technology (Shanghai, China); HEPES buffers (pH 8.0 and 7.4) from Macklin Biochemical (Shanghai, China); and LPS and RNase A from Solarbio Life Sciences (Beijing, China). Cell culture consumables included Transwell inserts (polycarbonate, 0.4 µm pore; Corning, New York, USA), DMEM (high glucose), and FBS from Gibco (New York, USA), while 0.25% trypsin, penicillin‐streptomycin, and Opti‐MEM reduced‐serum medium were sourced from Sigma (St. Louis, USA), HyClone (Logan, USA), and Thermo Fisher Scientific (Waltham, USA), respectively. All other chemicals and solvents were of standard analytical or reagent grade.

### 4.2 Cell Culture

The human cerebral microvascular endothelial cell line (hCMEC/D3) and the human microglial cell line (HMC3) were obtained from Shanghai Zhongqiaoxinzhou Biotechnology (Shanghai, China). The mouse brain microvascular endothelial cell line (bEnd.3), the mouse hippocampal neuronal cell line (HT22), and the mouse microglial cell line (BV2) were acquired from Haixing Biotechnology (Beijing, China). All cell lines were cultured in DMEM supplemented with 10% FBS and 1% penicillin‐streptomycin at 37°C in a humidified atmosphere of 5% CO_2_.

### 4.3 Animal Models and Ethics

All animal experiments were conducted in accordance with protocols approved by the Animal Care and Use Committee of Central South University (Approval No.: CSU‐2024‐0398). Male APP/PS1 transgenic mice (on a C57BL/6 background) and their wild‐type (WT) littermates aged 24 weeks were obtained from Cyagen Biosciences Inc. (Suzhou, China). Animals were housed under specific pathogen‐free (SPF) conditions with a controlled ambient temperature (22°C ± 2°C) and a 12‐h light/dark cycle, and were provided with free access to food and water.

### 4.4 Single‐Nucleus Transcriptome Analysis

We conducted a reanalysis of a publicly available snRNA‐seq dataset (GEO: GSE188545) [36], which profiled the human middle temporal gyrus from patients with AD and HCs. All analyses were performed using Seurat (v4.0) in R. Following standard quality control, genes detected in fewer than three nuclei were excluded, and nuclei were retained only if they expressed between 200 and 2,500 genes (nFeature_RNA) and exhibited mitochondrial UMI percentages below 15%.

Counts were normalized per sample using the NormalizeData function, and the top 2000 highly variable genes were selected with FindVariableFeatures. Datasets were integrated using IntegrateData to correct for batch effects. The integrated data were scaled and subjected to principal component analysis. A shared nearest‐neighbor (SNN) graph was constructed with FindNeighbors, and clusters were identified using FindClusters at a resolution of 0.5. Low‐dimensional embeddings were generated with UMAP for visualization. Cell types were annotated using canonical marker genes: cerebrovascular endothelial cells were identified by expression of *CDH5*, *CLDN5*, and *VWF*, and microglia subpopulation was defined by specific upregulation of *GPNMB*. To identify differentially expressed genes (DEGs) between AD and HC within specific cell populations, we used FindMarkers (wilcoxon test) and considered genes with |average log_2_ fold change| > 0.25 and Benjamini–Hochberg‐adjusted *p* < 0.05 as significant.

### 4.5 RNA‐Sequencing and Transcriptomic Analysis

hCMEC/D3 cells were treated with 5 µm Aβ_1__−__4__2_ or dimethyl sulfoxide (DMSO) for 48 h; HMC3 cells were treated with 5 µm Aβ_1__−__4__2_ or DMSO for 72 h; bEnd.3 cells were first transfected with 50 nm siPCSK9 or siNC using GMTrans Liposomal Transfection Reagent. After 24 h of transfection, the cells were treated with 500 nm Aβ_1__−__4__2_ for 4 h. Following removal of the Aβ_1__−__4__2_‐containing supernatant, the cells were further cultured for 24 h.

After interventions, total RNA was extracted from each group using Trizol reagent followed by chloroform extraction and isopropanol precipitation. The RNA samples were sent to Beijing Novogene Co., Ltd. for transcriptome sequencing. RNA integrity was assessed using the Agilent 2100 Bioanalyzer. The sequencing library was constructed through processes including mRNA enrichment, fragmentation, complementary DNA (cDNA) synthesis, and PCR amplification, followed by sequencing on the Illumina platform. Bioinformatic analysis was performed as follows: raw sequencing data were quality‐controlled and filtered using fastp; clean reads were aligned to the reference genome (human GRCh38 or mouse mm10) using HISAT2; gene expression levels were quantified using featureCounts (v2.0.6); DEGs were identified with DESeq2 (screening thresholds: |log_2_(fold change)| > 0 or |log_2_(fold change)| > 0.2, and *p* < 0.05); finally, Gene Ontology (GO) annotation and Kyoto Encyclopedia of Genes and Genomes (KEGG) pathway enrichment analyses of the DEGs were conducted using clusterProfiler.

To identify differentially expressed autophagy‐related genes (DEAGs), we systematically screened autophagy‐related genes by integrating existing database resources and transcriptomic analysis results. Specifically, 232 autophagy‐related genes were obtained from The Human Autophagy Database (https://www.autophagy.lu/), and 405 autophagy‐related genes were extracted from the Human Autophagy Modulator Database (HAMdb, http://hamdb.scbdd.com) [73] to construct an autophagy‐related gene set. Based on mRNA sequencing data provided by Novogene, DEGs between siNC combined with Aβ treatment and siPCSK9 combined with Aβ treatment in bEnd.3 cells were identified. Autophagy‐related genes were screened from these DEGs, ultimately identifying DEAGs under siPCSK9 intervention. The target genes were visualized using a clustered heatmap generated with the “pheatmap” R package. Further GO functional annotation and KEGG pathway enrichment analyses of the DEAGs were performed using the Metascape online platform [74] to elucidate their potential biological functions. A significance threshold was set at both *p*‐value and FDR < 0.05.

### 4.6 Preparation of Recombinant Human Aβ_1–42_ Oligomers

Aβ_1–42_ peptide (1 mg) was dissolved in 1 mL HFIP, sonicated for 30 min at room temperature, and dried under nitrogen to form a peptide film. After lyophilization, the film was aliquoted and stored at −80°C. For oligomer preparation, the film was dissolved in DMSO to obtain a 7.384 mm stock solution, which was then diluted in complete medium with 10% FBS and incubated at 37°C for 24 h to form oligomers. Following incubation, the solution was centrifuged at 16 000 × g for 20 min at 4°C to remove insoluble aggregates, and the supernatant containing soluble oligomers was carefully collected for subsequent cell treatment experiments.

### 4.7 Establishment of AD Cell Models

To model AD‐like damage in brain cell types, bEnd.3 and BV2 cells were treated with 5–20 µm Aβ_1__−__4__2_ oligomers for 24 h to simulate injured cerebralvascular endothelial cells and microglia, respectively. Additionally, BV2 microglial cells were stimulated with 1 µg/mL LPS for 24 h to establish an AD‐related neuroinflammatory model.

### 4.8 siRNA Transfection

For siRNA‐mediated knockdown, bEnd.3 and BV2 cells in the normal control group were transfected with 50 nm siNC. Experimental groups were transfected with either 50 nm siPCSK9, 50 nm siTRIB3, or a combination of both (50 nm each). Transfection was performed using GMTrans Liposomal Transfection Reagent according to the manufacturer's instructions. Briefly, bEnd.3 and BV2 cells (1.5–2 × 10^5^ cells/well) were seeded in 12‐well plates when cells reached approximately 75% confluency. siRNA and transfection reagent were separately diluted in Opti‐MEM reduced‐serum medium, incubated at room temperature for 5 min, combined, and incubated for another 30 min to form complexes. The mixture was then added to the cells. After 4–6 h, the medium was replaced with fresh complete medium. Knockdown efficiency was assessed at 24 or 48 h post‐transfection, and cells meeting efficiency criteria were used in subsequent experiments.

### 4.9 Preparation of TPNs

TPNs were synthesized according to established protocols from our research group using a metal ion‐catalyzed oxidative self‐polymerization strategy [50, 51, 52, 53]. Briefly, a 10 mm HEPES buffer (pH 8.0) was prepared at 25°C, and the final reaction volume was adjusted to 1 mL unless otherwise specified. EGCG was added to the HEPES buffer under continuous magnetic stirring to obtain a final concentration of 2.5 mm, followed by stirring for 30 min. MnCl_2_ solution was then slowly added dropwise to the reaction mixture under continuous stirring at 37°C to obtain a final concentration of 2 mm. The reaction was maintained at 37°C under continuous stirring for 2 h. The resulting product was centrifuged at 16 000 × g for 15 min at 4°C. The supernatant was discarded, and the nanoparticle pellet was washed twice with 10 mm HEPES buffer (pH 7.4). After each washing step, the sample was centrifuged at 16 000 × g for 15 min at 4°C. Finally, the pellet was collected, resuspended in 1 mL of 10 mm HEPES buffer (pH 7.4) to obtain the TPNs stock dispersion. The resulting TPNs were stored at 4°C protected from light until further use.

### 4.10 Preparation of SIT and FAM/Cy5.5‐Labeled SIT

To co‐deliver siPCSK9 and siTRIB3, the two siRNAs were incorporated during TPNs self‐assembly to prepare SIT nanoparticles. Briefly, 10 mm HEPES buffer (pH 8.0) was prepared at 25°C, and the final reaction volume was adjusted to 1 mL unless otherwise specified. EGCG was dissolved in the buffer under magnetic stirring to a final concentration of 2.5 mm.

siPCSK9 and siTRIB3 were premixed at an equimolar ratio. The final feeding concentration of each siRNA in the reaction system was 2 µm, corresponding to a total siRNA feeding concentration of 4 µm. The siPCSK9 sense strand was 5′‐GCACAUGCUUCAUGUCACATT‐3′, and the antisense strand was 5′‐UGUGACAUGAAGCAUGUGCTT‐3′. The siTRIB3 sense strand was 5′‐CCAAGUGUCCAGUCCUAAATT‐3′, and the antisense strand was 5′‐UUUAGGACUGGACACUUGGTT‐3′.

The siRNA mixture was slowly added dropwise to the EGCG solution under continuous stirring. After stirring for 30 min, MnCl_2_ solution was slowly added to the mixture to a final concentration of 2 mm, followed by continuous stirring at 37°C for 2 h. After the reaction, the mixture was centrifuged at 16 000 × g for 15 min at 4°C to remove unencapsulated siRNA and soluble reaction components. The pellet was collected and washed twice with 10 mm HEPES buffer (pH 7.4). After each washing step, the sample was centrifuged at 16 000 × g for 15 min at 4°C. Finally, the pellet was resuspended in 1 mL of 10 mm HEPES buffer (pH 7.4) to obtain the SIT nanoparticle dispersion.

For fluorescence‐tracking experiments, FAM‐ or Cy5.5‐labeled siRNAs were used in place of the corresponding unlabeled siRNAs, while the siPCSK9:siTRIB3 molar ratio and total siRNA feeding concentration were kept unchanged. All procedures involving fluorescently labeled siRNAs were performed under light‐protected conditions. The resulting fluorescent formulations were denoted FAM‐SIT and Cy5.5‐SIT, respectively.

### 4.11 Preparation of SITR and FAM/Cy5.5‐Labeled SITR

The brain‐enriched formulation SITR was prepared by modifying the SIT core with SH‐PEG_2__000_‐RAP. Briefly, the SIT core was first generated following the procedure described in Section 4.10 using the same siRNA sequences and formulation parameters: final reaction volume, 1 mL; EGCG, 2.5 mm; siPCSK9, 2 µm; siTRIB3, 2 µm; total siRNA feeding concentration, 4 µm; MnCl_2_, 2 mm; siRNA–EGCG pre‐complexation, 30 min; and MnCl_2_‐catalyzed self‐assembly, 2 h at 37°C.

After formation of the SIT core, SH‐PEG_2__000_‐RAP was directly added to the reaction mixture without intermediate purification to a final concentration of 0.4 mm. The mixture was further stirred at 37°C for 30 min to allow stable RAP modification on the nanoparticle surface. The mixture was then centrifuged at 16 000 × g for 15 min at 4°C to remove unbound RAP, unencapsulated siRNA, and soluble impurities. The supernatant was discarded, and the pellet was washed twice with 10 mm HEPES buffer (pH 7.4). After each washing step, the sample was centrifuged at 16 000 × g for 15 min at 4°C. Finally, the pellet was resuspended in 1 mL of 10 mm HEPES buffer (pH 7.4) to obtain the SITR nanoparticle dispersion.

FAM‐SITR and Cy5.5‐SITR were prepared using the same procedure, except that FAM‐ or Cy5.5‐labeled siRNAs were used instead of the corresponding unlabeled siRNAs. The siPCSK9:siTRIB3 molar ratio, total siRNA feeding concentration, EGCG concentration, MnCl_2_ concentration, and SH‐PEG_2__000_‐RAP concentration were kept unchanged. All procedures involving fluorescently labeled siRNAs were performed under light‐protected conditions. The resulting fluorescent formulations were denoted FAM‐SITR and Cy5.5‐SITR, respectively.

### 4.12 Physicochemical Characterization, Morphology, siRNA Encapsulation Efficiency, and RAP Grafting Rate of Nanoparticles

The hydrodynamic diameter, zeta potential, and PDI of TPNs, SIT, and SITR were measured using a Malvern Zetasizer Nano ZS90 instrument (Malvern Panalytical, UK). Samples were appropriately diluted in 10 mm HEPES buffer (pH 7.4) and analyzed at 25°C in quartz cuvettes.

Morphological examination and elemental composition analysis of SITR were performed using TEM (Titan G2 60–300, FEI, USA) combined with energy‐dispersive X‐ray spectroscopy (EDS). For TEM imaging, a small aliquot of SITR dispersion was dropped onto a copper grid and air‐dried at room temperature. The samples were imaged without staining. EDS elemental mapping was conducted to assess the spatial distribution of carbon (C), oxygen (O), nitrogen (N), phosphorus (P), sulfur (S), and manganese (Mn).

The encapsulation efficiency (EE) of siRNA in SIT and SITR was determined using agarose gel electrophoresis. Nanoparticles were prepared with different final siRNA feeding concentrations of 2, 4, and 8 µm. After centrifugation at 16 000 × g for 15 min at 4°C, the supernatant containing unencapsulated siRNA was collected. Samples were mixed with loading buffer and electrophoresed on a 2% agarose gel at 150 V for 15 min. Gels were stained with Gel‐Red and visualized using a ChemiDoc XRS+ imaging system (Bio‐Rad, USA). siRNA band intensities were quantified with Image Lab software (Bio‐Rad, USA), and EE was calculated as the following Equation (1):

(1) $$\begin{array}{ccc} \mathrm{EE}\left(\%\right) & = & \frac{\mathrm{Total}\;\mathrm{siRNA}\;\mathrm{input}-\mathrm{Unencapsulated}\;\mathrm{siRNA}\;\mathrm{in}\;\mathrm{supernatant}}{\mathrm{Total}\;\mathrm{siRNA}\;\mathrm{input}}\\ & & \times 100\% \end{array}$$

The GR of SH‐PEG_2__000_‐RAP onto SITR was evaluated using a total thiol assay kit. SITR formulations were prepared using different RAP feeding concentrations of 0.1, 0.2, 0.4, and 0.6 mm. After centrifugation at 16 000 × g for 15 min at 4°C, the thiol content in the nanoparticle pellet and supernatant was measured according to the manufacturer's instructions. The GR was calculated as the following Equation (2):

(2) $$\begin{array}{ccc} \mathrm{GR}\;\left(\%\right) & = & \frac{\mathrm{Thiol}\;\mathrm{content}\;\mathrm{associated}\;\mathrm{with}\;\mathrm{SITR}}{\mathrm{Thiol}\;\mathrm{content}\;\mathrm{associated}\;\mathrm{with}\;\mathrm{SITR}+\mathrm{Thiol}\;\mathrm{content}\;\mathrm{in}\;\mathrm{supernatant}}\\ & & \;\times 100\% \end{array}$$

### 4.13 In Vitro Characterization of Nanoparticle Stability and Release Profile

The colloidal stability of SIT and SITR was evaluated in different physiological media. Nanoparticle dispersions were centrifuged at 16 000 × g for 15 min at 4°C, and the pellets were resuspended in 10 mm HEPES buffer (pH 7.4), PBS (pH 7.4), normal saline (NS), or DMEM supplemented with 10% FBS. The dispersions were incubated at 37°C. At predetermined time points of 0, 12, 24, 36, and 48 h, the hydrodynamic diameter and PDI were measured to monitor colloidal stability and aggregation behavior.

The siRNA release profile of SITR was evaluated in two release media: PBS (pH 7.4), which mimics the extracellular circulation environment, and PBS (pH 7.4) containing 10 mm GSH, which mimics the intracellular reducing environment. SITR dispersions containing equivalent amounts of siRNA were centrifuged at 16 000 × g for 15 min at 4°C, and the pellets were resuspended in the release media. Each release system was divided into equal‐volume independent samples for the indicated time points, with identical SITR content and release volume in each sample. Samples were incubated at 37°C under shaking at 100 rpm. At 0.5, 1, 2, 4, 8, 12, 24, and 48 h, the corresponding independent samples were collected and centrifuged at 16 000 × g for 15 min at 4°C. The supernatants were collected and stored at −20°C until analysis. The amount of released siRNA in the supernatant was quantified by 2% agarose gel electrophoresis. The release profile was plotted according to the percentage of released siRNA relative to the initial siRNA amount.

### 4.14 Nuclease Resistance and Serum Stability Assay

To evaluate the protective effect of SITR against enzymatic and serum‐mediated degradation, both RNase protection and serum stability assays were performed. Free FAM‐labeled siRNA and FAM‐SITR containing equivalent amounts of siRNA were used in both experiments. For the nuclease resistance assay, samples were treated with RNase A at a concentration of 1 µg/mL for 0, 0.5, 1, and 2 h at 37°C. For the serum stability assay, samples were incubated in DMEM supplemented with 10% FBS for 0 and 24 h at 37°C. Following incubation, all samples underwent identical processing: reactions were terminated by adding 20 mm EDTA followed by heating at 65°C for 10 min to inhibit further nucleic acid degradation. Subsequently, 20 mm GSH was added to dissociate the nanoparticles and release the encapsulated siRNA. After centrifugation at 16 000 × g for 15 min at 4°C, the supernatant was collected and analyzed by 2% agarose gel electrophoresis to evaluate siRNA integrity.

### 4.15 Nanoparticle‐Mediated Cell Intervention

bEnd.3 and BV2 cells were subjected to various nanoparticle treatments for 24 h. The experimental groups included: PBS‐treated normal control; TPNs (19.2 µg/mL); SIT (containing 19.2 µg/mL TPNs, 0.9 µg/mL siPCSK9 and 0.9 µg/mL siTRIB3); and SITR (containing 19.2 µg/mL TPNs, 0.9 µg/mL siPCSK9 and 0.9 µg/mL siTRIB3, and 41.3 µg/mL SH‐PEG_2__000_‐RAP).

### 4.16 In Vitro Cytotoxicity Assay of Nanoparticles

bEnd.3, BV2, and HT22 cells (1 × 10^4^ cells/well) were seeded in 96‐well plates and treated with varying concentrations of TPNs, SIT, or SITR for 24 h. Cell viability was evaluated using the CCK‐8 assay by measuring the OD_4__5__0_ nm after 1–2 h of incubation.

### 4.17 In Vitro Targeted Cellular Uptake of Nanoparticles

Cellular uptake and targeting efficiency of SITR were evaluated in bEnd.3 and BV2 cells pre‐treated with 10 µm Aβ_1__−__4__2_ for 12 h. The following groups were established: cells incubated with naked siRNA, FAM‐SIT, or FAM‐SITR for 12 h; and cells pre‐treated with both Aβ_1__−__4__2_ and RAP followed by FAM‐SITR incubation (SITR+RAP group). After treatment, cells were washed with PBS, fixed with paraformaldehyde, and nuclei were stained with DAPI. Cellular uptake was qualitatively assessed by fluorescence microscopy based on FAM green fluorescence. For quantitative analysis, Aβ_1__−__4__2_‐pre‐treated cells were incubated with the above formulations for 2, 4, and 8 h, followed by PBS washing and collection. FAM fluorescence intensity was measured using flow cytometry to compare nanoparticle internalization over time.

### 4.18 Nanoparticle Penetration Across In Vitro BBB Model

An in vitro BBB model was established by seeding 5×10^4^ bEnd.3 cells into the upper chamber of a 0.4 µm polycarbonate membrane Transwell system. The TEER was monitored daily using a Millicell ERS‐2 volt‐ohm meter. The TEER value (Ω·cm^2^) was calculated as (R − R_0_) × A, where R is the measured resistance of the cell monolayer, R_0_ is the resistance of a cell‐free insert, and A is the effective membrane area. After 6 days, when TEER stabilized at 29.8 Ω·cm^2^, indicating intact monolayer formation, permeability assays were initiated. bEnd.3 cells were pre‐treated with 10 µm Aβ_1__−__4__2_ for 12 h. Naked siRNA, FAM‐SIT, or FAM‐SITR were added to the upper chamber, while phenol red‐free DMEM with 10% FBS was placed in the lower chamber. The filtrate in the lower chamber was collected at 1, 3, 6, and 12 h, with fresh medium replenished at each interval. FAM fluorescence in the collected samples was quantified using a microplate reader to evaluate the BBB penetration capacity of SITR.

### 4.19 Endosomal/Lysosomal Escape Assay

To assess the endosomal/lysosomal escape capability of nanoparticles, bEnd.3 and BV2 cells (4×10^4^ cells per dish) were pretreated with 10 µm Aβ_1__−__4__2_ for 12 h, followed by incubation with FAM‐SITR for 2, 4, 6, and 8 h. Cells were then washed with PBS and stained with 200 nm LysoTracker Red (37°C, 60 min) to label lysosomes. After fixation with paraformaldehyde and nuclear staining with DAPI, the subcellular distribution of nanoparticles (green fluorescence) and lysosomes (red fluorescence) was visualized using a confocal laser scanning microscope (LSM900, Zeiss, Germany). Co‐localization analysis was performed to evaluate the endosomal/lysosomal escape efficiency.

### 4.20 RT‐qPCR

Total RNA was extracted from cells or mouse brain tissues using the M5 Universal Plus RNA Mini Kit (Mei5bio, China, MF167‐01). cDNA was synthesized using HiScript II QRT SuperMix for qPCR (Vazyme Biotech, China) on a Bio‐Rad PCR thermal cycler, and quantitative PCR was performed with ChamQ Universal SYBR qPCR Master Mix (Vazyme Biotech, China) on a CFX Connect Real‐Time PCR Detection System (Bio‐Rad, USA). The relative expression levels of target genes were calculated using the 2^−^ΔΔCt method with normalization to *Actb*. The primer sequences used were listed in Table S3.

### 4.21 Protein Extraction and WB Analysis

Total protein was extracted from cells or mouse brain tissues using radioimmunoprecipitation assay (RIPA) buffer (Boster Biological Technology, China) supplemented with protease and phosphatase inhibitors (Seven Biotechnology, China). Protein concentration was determined using a BCA Protein Assay Kit (Boster Biological Technology, China). Equal amounts of protein were separated by SDS‐PAGE on 4%–12%, 10%, or 15% polyacrylamide gels and transferred to 0.45 µm Polyvinylidene difluoride (PVDF) membranes (Millipore, USA). After blocking with 5% non‐fat milk, membranes were incubated overnight at 4°C with the following primary antibodies: anti‐PCSK9 (1:1000, #ab185194, Abcam), anti‐LRP1 (1:2000, #A0633, Abclonal), anti‐LC3B (1:2000, #ET1701‐65, Huabio), anti‐p62 (1:5000, #HA721171, Huabio), anti‐GAPDH (1:50000, #ET1601‐4, Huabio), anti‐β‐tubulin (1:2000, #10094‐1‐AP, Proteintech), and anti‐β‐actin (1:20000, #66009‐1‐Ig, Proteintech). Membranes were then incubated with HRP‐conjugated Goat anti‐Mouse IgG (1:20000, #HA1006, Huabio) or HRP‐conjugated Goat anti‐Rabbit IgG (1:20000, #HA1001, Huabio) at room temperature for 1 h. Protein bands were visualized using an ultra‐sensitive ECL chemiluminescence reagent (Beyotime Biotechnology, China) and imaged with a ChemiDoc XRS+ system (Bio‐Rad, USA). Quantification was performed using Image Lab software (Bio‐Rad, USA), with GAPDH, β‐tubulin, or β‐actin as the loading control. The relative expression of each target protein was calculated as the ratio of its optical density to that of the reference protein.

### 4.22 Cellular Immunofluorescence Staining

bEnd.3 cells grown on coverslips were processed after transfection and treatment. For basal TRIB3 expression analysis after PCSK9 silencing, bEnd.3 cells were transfected with 50 nm siNC or siPCSK9 for 24 h in the absence of exogenous Aβ. Cells were fixed with 4% paraformaldehyde, permeabilized, blocked with 3% BSA, and incubated with rabbit anti‐TRIB3 antibody (1:2000, #GB113809, Servicebio) overnight at 4°C. After washing with PBS, coverslips were incubated with Cy3‐conjugated anti‐rabbit secondary antibody (Beyotime Biotechnology, China) for 1 h at room temperature. Nuclei were counterstained with DAPI, and coverslips were mounted with anti‐fade medium for fluorescence microscopy.

For mechanistic co‐localization analysis of TRIB3 with autophagy‐related proteins, sequential Tyramide Signal Amplification (TSA)‐based staining was performed. Following fixation with 4% paraformaldehyde and permeabilization, endogenous peroxidase was quenched with 3% H_2_O_2_, and non‐specific binding sites were blocked with 3% BSA. Coverslips were first incubated with rabbit anti‐TRIB3 (1:2000, #GB113809, Servicebio) at 4°C overnight, followed by HRP‐conjugated anti‐rabbit secondary antibody and Tyramide‐555 amplification. After elution with antibody elution buffer (#G1266, Servicebio) and PBS rinsing, coverslips were incubated with either anti‐PPARα (1:2000, #GB11163, Servicebio) or anti‐p62 (1:5000, #GB11531, Servicebio) overnight at 4°C, followed by HRP‐conjugated secondary antibody and Tyramide‐488 amplification. Nuclei were counterstained with DAPI, and coverslips were mounted with anti‐fade medium for fluorescence microscopy.

Images from all groups within the same experiment were acquired under identical exposure and acquisition settings. Fluorescence intensity and co‐localization were analyzed using ImageJ/Fiji software (National Institutes of Health, Bethesda, MD, USA). For fluorescence quantification, mean fluorescence intensity (MFI) was measured from randomly selected fields. Where indicated, relative fluorescence intensity was normalized to the mean value of the corresponding control group, which was set to 100%.

### 4.23 Aβ Uptake Assay

To evaluate Aβ uptake in bEnd.3 and BV2 cells, the cells were subjected to two intervention strategies for 24 h: gene interference and nanoparticle treatment. For qualitative analysis, cells were incubated with 500 nm 5‐TAMRA‐labeled Aβ_1__−__4__2_ in DMEM containing 5% FBS at 37°C for 4 h, followed by fixation with 4% paraformaldehyde and DAPI staining. Internalization of Aβ was visualized using an LSM900 confocal microscope (Zeiss, Germany). For quantitative assessment, cells were treated with 500 nm 5‐TAMRA‐Aβ_1__−__4__2_ for 2, 4, or 8 h, washed with PBS to remove unbound Aβ, and analyzed using a CytoFLEX flow cytometer (Beckman Coulter, China) to measure cell‐associated fluorescence [75].

### 4.24 Aβ Non‐Specific Binding Assay

After gene interference for 24 h, bEnd.3 and BV2 cells were incubated with 500 nm 5‐TAMRA‐Aβ_1__−__4__2_ in DMEM with 5% FBS at 4°C for 2, 4, or 8 h to inhibit internalization. After washing with PBS to remove unbound Aβ, cells were analyzed by flow cytometry to quantify surface‐bound fluorescence [75].

### 4.25 Aβ Degradation Assay

bEnd.3 and BV2 cells were subjected to gene interference or nanoparticle treatment for 24 h. Cells were then incubated with 500 nm Aβ_1__−__4__2_ in DMEM with 5% FBS at 37°C for 4 h, washed, and lysed with 5 m guanidine HCl in 50 mm Tris‐HCl (pH 8.0). Cell‐associated Aβ was measured using a human Aβ_1__−__4__2_ ELISA kit. For degradation assessment, after the 4 h uptake, the medium was replaced with fresh complete medium with or without autophagy inhibitors (2 mm 3‐MA or 25 µm HCQ) and incubated for 24 h. Aβ levels were again measured by ELISA [75]. The Aβ_1__−__4__2_ clearance rate (%) was calculated using the following Equation (3):

(3) $$\begin{array}{ccc} & & A\beta_{1-42}\;\mathrm{clearance}\;\left(\%\right)\\ & & \;=\frac{A\beta \;\mathrm{level}\;\mathrm{after}\;4\;h\;\mathrm{uptake}-A\beta \;\mathrm{level}\;\mathrm{after}\;24\;h\;\mathrm{degradation}}{A\beta \;\mathrm{level}\;\mathrm{after}\;4\;h\;\mathrm{uptake}}\\ & & \;\times 100\% \end{array}$$

### 4.26 In Vitro BBB Model and Aβ_1–42_ Transcytosis Assay

An in vitro BBB model was established by seeding 5 × 10^4^ bEnd.3 cells into the upper chamber of a 24‐well Transwell insert equipped with a 0.4 µm pore‐size polycarbonate membrane. The TEER was monitored daily until values stabilized, indicating the formation of a confluent endothelial monolayer. Two intervention strategies, gene interference or nanoparticle treatment, were implemented for 24 h. To evaluate Aβ_1–42_ clearance in the Aβ transcytosis assay, 100 nm 5‐TAMRA‐Aβ₁_−__4__2_ and 2.5 µm FITC‐inulin were added to either the apical (A) compartment (blood side) to study A→B transport, or the basolateral (B) compartment (brain tissue side) to study B→A transport. After 6 h of incubation, medium from both chambers was collected. Fluorescence intensity was quantified using an Infinite M200 PRO microplate reader (Tecan, Austria), with 5‐TAMRA‐Aβ₁_−__4__2_ measured at Ex/Em = 560/592 nm and FITC‐inulin at Ex/Em = 485/520 nm. The clearance quotients (CQ) for A→B (5‐TAMRA‐Aβ₁_−__4__2_ CQ_A→B_) and B→A (5‐TAMRA‐Aβ₁_−__4__2_ CQ_B→A_) were calculated according to the following Equations (4 and 5) to evaluate the transcytosis of Aβ in the BBB model [76].

(4) $$\begin{array}{ccc} \;5 & - & \mathrm{TAMRA}-A\beta_{1-42}\;\mathrm{CQA}\to B\\ & & =\frac{\frac{5-\mathrm{TAMRA}-A\beta_{1-42}\mathrm{in}\;\mathrm{basolateral}\;\mathrm{compartment}}{\mathrm{Total}\;5-\mathrm{TAMRA}-A\beta_{1-42}}}{\frac{\mathrm{FITC}-\mathrm{inulin}\;\mathrm{in}\;\mathrm{basolateral}\;\mathrm{compartment}}{\mathrm{Total}\;\mathrm{FITC}-\mathrm{inulin}}} \end{array}$$

(5) $$5-\mathrm{TAMRA}-A\beta_{1-42}\;\mathrm{CQB}\to A=\frac{\frac{5-\mathrm{TAMRA}-A\beta_{1-42}\;\mathrm{in}\;\mathrm{apical}\;\mathrm{compartment}}{\mathrm{Total}\;5-\mathrm{TAMRA}-A\beta_{1-42}}}{\frac{\mathrm{FITC}-\mathrm{inulin}\;\mathrm{in}\;\mathrm{apical}\;\mathrm{compartment}}{\mathrm{Total}\;\mathrm{FITC}-\mathrm{inulin}}}$$

The total fluorescence intensity of 5‐TAMRA‐Aβ₁_−__4__2_ and FITC‐inulin was defined as the sum of the fluorescence intensities measured in the apical and basolateral compartments.

### 4.27 In Vitro Cerebrovascular Endothelial Cells Barrier Function

After a confluent monolayer of bEnd.3 cells was established in the upper chamber of a Transwell insert (0.4 µm PC membrane), as confirmed by stable TEER, the cells were subjected to interventions including gene interference or nanoparticle treatment. All groups were then exposed to 20 µm recombinant human Aβ₁_−__4__2_ for 24 h. Barrier integrity was assessed by adding 1 mg/mL FITC‐dextran (4 kDa) to the upper chamber. After 1 h, fluorescence in the lower chamber was quantified using a microplate reader (Ex/Em = 485/520 nm), and the flux of FITC‐dextran was calculated to evaluate paracellular permeability.

### 4.28 Analysis of Autophagy in Cerebrovascular Endothelial Cells

Autophagic activity in bEnd.3 cells was evaluated using the following integrated approaches: MDC staining, TEM, WB of autophagy markers, and live‐cell imaging of autophagic flux with a dual fluorescent reporter.

For MDC staining, cells treated with 500 nm or 5 µm Aβ₁_−__4__2_ for 24 h were stained according to the manufacturer's protocol to visualize autophagic vacuoles. For TEM analysis, cells pretreated with PBS, TPNs, or SITR for 24 h and then exposed to 20 µm Aβ₁_−__4__2_ for an additional 24 h were processed through glutaraldehyde and osmium tetroxide fixation, graded ethanol‐acetone dehydration, resin embedding, and polymerization. Ultrathin sections (70–90 nm) were prepared with a Leica UC7 ultramicrotome, stained with uranyl acetate and lead citrate, and imaged using a HITACHI H‐7800 TEM (Hitachi, Japan). Parallel samples from the same treatment conditions were subjected to WB to examine protein levels of LC3‐II and p62.

To dynamically monitor autophagic flux, bEnd.3 cells were transfected with the mCherry‐GFP‐LC3B plasmid. In the gene interference group, cells were co‐transfected with 1 µg of plasmid along with 50 nm siPCSK9, siTRIB3, their combination, or siNC for 24 h, then treated with 20 µm Aβ₁_−__4__2_ for another 24 h. In the nanoparticle treatment group, cells transfected with 1 µg of plasmid were incubated with PBS, TPNs, or SITR for 24 h before Aβ₁_−__4__2_ exposure. Autophagic flux was qualitatively evaluated by confocal microscopy through observation of autophagosomes (GFP^+^mCherry^+^, yellow/orange puncta) and autolysosomes (GFP^−^mCherry^+^, red puncta), with the relative abundance and distribution of puncta reflecting autophagic degradation activity [77, 78, 79].

### 4.29 Detection of ROS and NO

Intracellular ROS levels were measured using the fluorescent probe DCFH‐DA. For fluorescence imaging, cells were incubated with DCFH‐DA at 37°C for 30 min, washed, and imaged with a Cytation5 microscope (BioTek, USA). For quantitative analysis, cells were collected after DCFH‐DA loading and analyzed by flow cytometry (CytoFLEX, Beckman Coulter) to detect FITC fluorescence. NO production was assessed by measuring nitrite accumulation in cell supernatants using a Griess reagent kit. After mixing 50 µL supernatant with Griess Reagents I and II, absorbance was measured at 540 nm using an Infinite M200 PRO microplate reader (Tecan, Austria).

### 4.30 Evaluation of Microglial Polarization and Inflammatory Response

The polarization state of BV2 microglial cells was evaluated by analyzing characteristic surface markers and cytokine secretion profiles. Cells were seeded at a density of 2×10^5^ cells per well in 12‐well plates and subjected to two treatment paradigms: gene interference or nanoparticle treatment for 24 h, followed by stimulation with LPS (1 µg/mL) for another 24 h to induce pro‐inflammatory activation and evaluate therapeutic interventions. Phenotypic polarization was analyzed by flow cytometry using fluorochrome‐conjugated antibodies against M1‐like markers (CD80, CD86) and the M2‐like marker (CD206). Polarization toward pro‐inflammatory states was further confirmed by quantifying characteristic cytokines (IL‐6, IL‐1β, TNF‐α, MCP‐1) in cell supernatants using commercial ELISA kits according to the manufacturer's instructions.

### 4.31 Assessment of Cellular Viability and Apoptosis

Cellular viability, cytotoxicity, and apoptotic response were evaluated using complementary assays. For viability assessment, bEnd.3 or BV2 cells were seeded at a density of 1×10^4^ cells per well in 96‐well plates and subjected to various treatments. These included gene transfection or nanoparticle‐based interventions, followed by exposure to Aβ₁_−__4__2_. Cells were incubated with 10% CCK‐8 reagent (APExBIO, USA) at 37°C for 1–2 h, and absorbance at 450 nm was measured using an Infinite M200 PRO microplate reader (Tecan, Austria). Parallel samples were stained with Calcein AM/PI working solution (1:1:1000) at 37°C for 30 min in the dark, and live (green) and dead (red) cells were visualized using a Cytation5 fluorescence microscope (BioTek, USA).

For apoptosis detection, 2×10^5^ bEnd.3 cells per well in 12‐well plates were treated with PBS, TPNs, or SITR for 24 h followed by intervention with 20 µm Aβ₁_−__4__2_ for another 24 h. Cells were digested with EDTA‐free trypsin, collected, and resuspended in 100 µL Annexin V binding buffer per sample. After staining with 5 µL Annexin V/Alexa Fluor 647 and 10 µL PI (20 µg/mL) at room temperature for 15 min in the dark, samples were diluted with 400 µL Annexin V binding buffer and analyzed using a BD FACSCalibur flow cytometer (BD Biosciences). Data processing was performed using FlowJo v10.8 software (TreeStar, USA).

### 4.32 In Vivo Biodistribution and Cellular Uptake

To evaluate in vivo biodistribution and brain‐enriched delivery, Cy5.5‐labeled siRNA was used as a fluorescent tracer. 6‐month‐old APP/PS1 mice were intravenously injected with 100 µL of one of the following: NS, free Cy5.5‐siRNA (Cy5.5‐siPCSK9/siTRIB3, 1375.9 µg/kg each), Cy5.5‐SIT (containing 31.0 mg/kg TPNs and 1375.9 µg/kg each Cy5.5‐siRNA), or Cy5.5‐SITR (containing 31.0 mg/kg TPNs, 1375.9 µg/kg each Cy5.5‐siRNA, and 66.7 mg/kg SH‐PEG_2__000_‐RAP). Longitudinal whole‐body fluorescence imaging was performed at 2, 4, 8, 12, and 24 h post‐injection using an AniView100 imaging system (Boluteng Biotechnology, China). Imaging parameters were kept consistent across groups within each imaging experiment. For longitudinal quantification, the Cy5.5‐associated fluorescence signal in the cranial region was quantified by manually drawing regions of interest (ROIs) over the cranial area of each mouse using the imaging software. The same region‐selection criteria were applied across groups and time points, and the signal was expressed as average radiant efficiency.

At 24 h post‐injection, mice were euthanized, and the brain and major organs (heart, liver, spleen, lung, kidney) were excised and imaged ex vivo under the same imaging conditions. For ex vivo biodistribution analysis, ROIs were drawn over each collected organ, and average radiant efficiency was quantified using the imaging software. For relative organ distribution analysis, the signal from each organ was normalized to the mean kidney signal within the corresponding treatment group, which was set to 1.

To assess cell‐associated brain distribution, brain tissues collected at 24 h post‐injection were processed for immunofluorescence staining of CD31^+^ cerebrovascular endothelial cells and IBA1^+^ microglia. Fresh‐frozen brain sections (10 µm) were prepared, permeabilized with 0.3% Triton X‐100, and blocked with goat serum. Sections were immunostained with anti‐IBA‐1 (1:10000, #AFRM0011, Aifang) and anti‐CD31 (1:3000, #AB281583, Abcam) antibodies overnight at 4°C, followed by incubation with fluorophore‐conjugated secondary antibodies and DAPI. Cy5.5‐labeled siRNA‐associated signals were assessed by fluorescence microscopy based on their spatial association with IBA1^+^ microglia or CD31^+^ cerebrovascular endothelial cells.

### 4.33 Experimental Design and Drug Administration in Animal Models

24‐week‐old APP/PS1 mice (*n* = 8–9 per group) were randomly assigned to four experimental groups, while age‐matched WT C57BL/6 littermates were included as a normal control group. All groups received weekly tail vein injections for six consecutive weeks. The APP/PS1 groups were administered the following: normal saline (NS group), TPNs (20.7 mg/kg, TPNs group), SIT (containing 20.7 mg/kg TPNs, 917.2 µg/kg siPCSK9 and 917.2 µg/kg siTRIB3, SIT group), or SITR (containing 20.7 mg/kg TPNs, 917.2 µg/kg siPCSK9 and 917.2 µg/kg siTRIB3, 44.5 mg/kg SH‐PEG_2__000_‐RAP, SITR group). The WT control group received NS. Throughout the treatment period, the body weight of all mice was monitored every three days to assess overall health and potential treatment‐related toxicity.

### 4.34 Behavioral Tests

Behavioral tests were performed to evaluate spontaneous locomotor activity, anxiety‐like behavior, recognition memory, working memory, and spatial learning and memory. Behavioral testing was initiated 24 h after the final treatment. The tests were conducted in the following order: open field test, NOR test, Y‐maze test, and MWM test. Before each test, mice were transferred to the behavioral testing room and allowed to habituate for at least 30 min. All behavioral experiments were performed during the same period each day under stable illumination and quiet environmental conditions. After each mouse was tested, the apparatus and objects were cleaned with 75% ethanol and allowed to dry completely to minimize olfactory cues. Behavioral data were recorded and analyzed using the SMART 3.0 automated video‐tracking system (Panlab, Spain). Investigators responsible for behavioral analysis were blinded to group allocation.

In the open field test, mice were gently placed in the center of a 40 × 40 × 30 cm arena and allowed to freely explore for 5 min while their movement trajectory, time spent in the central area, and total moving distance were recorded to assess spontaneous activity and anxiety‐like behavior.

The NOR test consisted of a training phase and a test phase and was performed in the same 40 × 40 × 30 cm arena. During the training phase, each mouse was placed in an arena containing two identical objects and allowed to explore freely for 10 min. After training, the mouse was returned to its home cage. 6 h later, the test phase was performed by replacing one familiar object with a novel object with a different shape and color. The mouse was allowed to explore freely for another 10 min. The time spent exploring the novel and familiar objects was recorded. Object exploration was defined as direct sniffing or touching of the object with the nose oriented toward the object; climbing on or sitting near the object was not counted as exploration. The discrimination index was calculated as follows: Discrimination index = (Time exploring the novel object − Time exploring the familiar object) / (Time exploring the novel object + Time exploring the familiar object).

For the Y‐maze test, each mouse was placed at the end of one arm of a Y‐shaped maze and allowed to freely explore for 8 min. The sequence of arm entries, total number of arm entries, and movement trajectory were recorded. An arm entry was counted only when all four paws entered the arm. Starting from the third arm entry, a correct spontaneous alternation was counted if the mouse entered an arm different from the previous two consecutive entries. Spontaneous alternation accuracy (%) was calculated as follows: Spontaneous alternation accuracy (%) = [Number of correct alternations / (Total number of arm entries − 2)] × 100%.

Spatial learning and memory were assessed using the MWM test. The test was conducted in a circular pool (100 cm in diameter, 50 cm in height) with water temperature maintained at 22°C ± 1°C. The test consisted of a 5‐day acquisition training phase followed by a probe trial on day 6. During the acquisition phase, a hidden platform (10 cm in diameter) was submerged 1 cm below the water surface in a fixed location within the target quadrant. Each mouse received four training trials per day from different starting points. The starting positions were pseudo‐randomized and counterbalanced across groups. Each trial lasted for a maximum of 60 s, and the escape latency, defined as the time required to find the hidden platform, was recorded. Mice that found the platform were allowed to stay on it for 5 s; mice that failed to find the platform within 60 s were gently guided to the platform and allowed to remain there for 5 s. On day 6, a probe trial was conducted with the platform removed. Each mouse was released from the quadrant opposite to the target quadrant and allowed to swim freely for 60 s. The time spent in the target quadrant, the number of crossings over the platform's original location, and the swimming speed were recorded to evaluate spatial memory retention and exclude potential motor impairment.

### 4.35 Immunofluorescence Staining of Brain Tissue Sections

Paraffin‐embedded brain sections (4 µm) were prepared from hemispheres collected after transcardial perfusion with ice‐cold saline, followed by immersion fixation in 4% paraformaldehyde at 4°C for 24–48 h. Sections were subjected to dewaxing, rehydration, and antigen retrieval in citrate buffer (pH 6.0). Endogenous peroxidase was inactivated with 3% H_2_O_2_, and nonspecific binding sites were blocked with normal goat serum. For TSA‐based sequential staining, sections were first incubated overnight at 4°C with primary antibodies. The following primary antibodies were used: anti‐TRIB3 (1:1000, #66702‐1‐Ig, Proteintech), anti‐6E10 (1:500, #SIG‐39300, BioLegend), anti‐CD31 (1:3000, #AB281583, Abcam), anti‐IBA1 (1:10000, #AFRM0011, AiFang), anti‐ZO‐1 (1:1000, #21773‐1‐AP, Proteintech), anti‐Occludin (1:2000, #GB111401‐100, Servicebio), anti‐Claudin5 (1:200, #ET1703‐58, Huabio), anti‐LC3B (1:1000, #AB48394, Abcam), and anti‐p62 (1:500, #AB91526, Abcam). After washing with PBST, sections were incubated with an HRP‐conjugated secondary antibody for 30 min at room temperature, followed by signal amplification using Tyramide‐570. For multiplexed staining, antibody complexes were eluted after the first round of staining, and the entire procedure (from antigen retrieval to tyramide development) was repeated sequentially using a second primary antibody and Tyramide‐520. Finally, nuclei were counterstained with DAPI, and slides were mounted with anti‐fade mounting medium for observation under a Zeiss Axio Observer 5 fluorescence microscope.

For quantification of endothelial‐associated TRIB3 fluorescence under AD‐like pathological conditions, brain sections from untreated 6‐month‐old APP/PS1 mice and age‐matched WT littermate controls were co‐stained with anti‐TRIB3 and anti‐CD31 antibodies. This analysis was performed using an independent untreated cohort, separate from the six‐week treatment cohort used for therapeutic efficacy and safety evaluation. CD31^+^ vessels with clearly identifiable vascular morphology were manually selected as ROIs based on the CD31 channel, and the same ROIs were then applied to the TRIB3 channel to measure TRIB3 MFI. Six CD31^+^ vascular ROIs were analyzed from each mouse. For statistical analysis, the mean value of the six ROI measurements from each mouse was used as one biological replicate. TRIB3 MFI was quantified using ImageJ/Fiji software (National Institutes of Health, Bethesda, MD, USA), and relative TRIB3 fluorescence intensity was normalized to the mean value of the WT littermate control group, which was set to 100%.

For other tissue immunofluorescence quantifications, MFI, positive area, and area ratio were analyzed using ImageJ/Fiji software. Where indicated, relative MFI values were normalized to the mean value of the corresponding control group, which was set to 100%. Positive area and area‐ratio measurements were analyzed using the original quantified values unless otherwise stated in the corresponding figure legends.

### 4.36 Measurement of Aβ_1–40_ and Aβ_1–42_ in Mouse Brain Tissue and Plasma

Following behavioral tests, cerebral cortex and hippocampal tissues were rapidly dissected on ice, rinsed with ice‐cold PBS, weighed, and minced. Tissues were homogenized in Tris‐buffered saline (50 mm Tris‐HCl, pH 7.4, 150 mm NaCl) containing protease inhibitors at a 1:9 (w/v) ratio using a tissue homogenizer. The homogenate was centrifuged at 10 000 × g for 30 min at 4°C. The supernatant (Tris‐soluble fraction) was collected and stored at −80°C for soluble Aβ analysis. The pellet was resuspended in 50 mm Tris‐HCl buffer (pH 8.0) containing 5 m guanidine hydrochloride, sonicated, and lysed on ice for 30 min. After centrifugation at 10 000 × *g* for 30 min, the supernatant (guanidine‐soluble fraction) was stored at −80°C for insoluble Aβ analysis. Both brain fractions and plasma samples were analyzed for human Aβ_1–40_ and Aβ_1–42_ levels using commercial ELISA kits according to the manufacturer's instructions. Results were normalized to total protein concentration determined by BCA assay [80].

### 4.37 Measurement of Inflammatory Factors in Mouse Brain Tissue

Tris‐soluble brain fractions were analyzed for inflammatory cytokines (IL‐6, IL‐1β, TNF‐α, MCP‐1) using ELISA kits according to manufacturer's instructions, with results normalized to total protein concentration.

### 4.38 Nissl Staining

Brain paraffin sections were deparaffinized and rehydrated. For Nissl staining, sections were stained with methylene blue, differentiated until Nissl bodies were visible, treated with ammonium molybdate, and mounted for observation. Neuronal morphology was analyzed in the hippocampal regions.

### 4.39 In Vivo BBB Permeability Assessment

Following behavioral tests, mice were administered FITC‐dextran (40 kDa; 4 mg/100 µL in PBS, 100 µL per mouse) via tail vein injection. After 15 min, the mice were transcardially perfused with ice‐cold PBS at a rate of 4 mL/min for 5 min to thoroughly flush out intravascular FITC‐dextran. Brains were rapidly dissected, flash‐frozen in liquid nitrogen, and embedded in Optimal Cutting Temperature (OCT) compound. Coronal sections (10 µm thickness) were prepared using a cryostat, and FITC‐dextran extravasation was visualized using a confocal microscope (LSM900, Zeiss, Germany) [81].

### 4.40 In Vivo Systemic Toxicity Assessment of Nanoparticles

Systemic toxicity was evaluated in mice receiving the same treatment regimen as described in Section 4.33. Briefly, 24‐week‐old male APP/PS1 mice were randomly assigned to the AD+NS, AD+TPNs, AD+SIT, and AD+SITR groups, and age‐matched WT mice receiving NS were used as controls. Mice received intravenous tail vein injections once weekly for six consecutive weeks. The administered doses were the same as those described in Section 4.33. Body weight was monitored every 3 days throughout the treatment period.

After completion of treatment and behavioral assessment, mice were fasted overnight with free access to water and deeply anesthetized with pentobarbital sodium (150 mg/kg, intraperitoneally). Blood samples were collected by cardiac puncture under deep anesthesia before transcardial perfusion. For serum biochemical and metabolic analyses, blood was allowed to clot at room temperature and then centrifuged at 1,200 × g for 15 min at 4°C to obtain serum. Serum biochemical and metabolic parameters, including AST, ALT, ALP, TP, BUN, CRE, UA, CK‐MB, LDH, GLU, TCHO, TG, LDL‐C, and HDL‐C, were measured using a TBA‐40FR automatic biochemical analyzer (Toshiba, Japan) according to the manufacturer's instructions. Serum insulin and cTnI were measured using ELISA kits. The homeostatic model assessment of insulin resistance (HOMA‐IR) was calculated as fasting insulin (mIU/L) × fasting glucose (mm) / 22.5. Serum biochemical and metabolic analyses were performed with *n* = 5 mice per group.

For hematological analysis, whole blood was collected into anticoagulant tubes. WBC, RBC, HGB, and PLT were measured by Servicebio (Wuhan, China) using an automated hematology analyzer. Hematological analysis was performed with *n* = 3 mice per group.

After blood collection, mice were euthanized by exsanguination during transcardial perfusion with ice‐cold NS under deep anesthesia. Death was confirmed by cessation of heartbeat and respiration before tissue collection. Major organs, including the heart, liver, spleen, lungs, and kidneys, were harvested for histopathological analysis. Tissues were fixed in 4% paraformaldehyde, embedded in paraffin, sectioned at 4 µm, and stained with H&E according to standard procedures. Histopathological scoring was performed on H&E‐stained sections of major organs, including the heart, liver, spleen, lungs, and kidneys. Five mice were analyzed in each group. Scoring was performed according to the International Harmonization of Nomenclature and Diagnostic Criteria (INHAND) framework and general principles for histopathological severity grading [82, 83]. Organ‐specific pathological changes were evaluated according to the histological characteristics of each organ, including necrosis, inflammatory cell infiltration, hemorrhage/congestion, connective tissue proliferation, alveolar wall thickening, and renal tubular epithelial degeneration. Each lesion parameter was scored using a five‐grade semi‐quantitative scale: 0, absent; 1, minimal; 2, mild; 3, moderate; and 4, severe. For each mouse, an organ‐specific histopathological score was calculated by averaging the scores of the evaluated lesion parameters.

### 4.41 Hemocompatibility Assay

A 2% (v/v) mouse red blood cell (RBC) suspension from C57BL/6 mice was prepared. RBCs were incubated with TPNs, SIT, or SITR (50–800 µg/mL, based on TPNs concentration), saline (negative control), or water (positive control) at 37°C for 3 h. The absorbance of the supernatant was measured at 576 nm using a microplate reader. Hemolysis rate (%) was calculated using the following Equation (6):

(6) $$\begin{array}{ccc} & & \mathrm{Hemolysis}\mathrm{rate}\left(\%\right)\\ & & \;=\frac{\mathrm{Sample}\;\mathrm{absorbance}-\mathrm{Negative}\;\mathrm{control}\;\mathrm{absorbance}}{\mathrm{Positive}\;\mathrm{control}\;\mathrm{absorbance}-\mathrm{Negative}\;\mathrm{control}\;\mathrm{absorbance}}\\ & & \;\times \;100\% \end{array}$$

A hemolysis rate exceeding 5% was considered indicative of significant hemolytic activity.

### 4.42 Statistical Analysis

All statistical analyses were performed using GraphPad Prism software (v10.5.0, San Diego, CA, USA). Data quantification and preprocessing were performed as described in the corresponding experimental sections, including normalization, image quantification, and histopathological scoring where applicable.

Unless otherwise specified, data are presented as mean ± standard deviation (SD). Semi‐quantitative histopathological scores are presented as median and interquartile range (IQR). For in vitro experiments, *n* represents independent biological experiments unless otherwise indicated. For behavioral tests, ELISA assays, biochemical analyses, hematological analyses, body weight measurements, and other animal‐level measurements, *n* represents the number of mice. For tissue immunofluorescence and histological quantification, multiple fields or ROIs were analyzed for each mouse. Detailed sample sizes, field/ROI numbers, and data presentation are provided in the corresponding figure legends.

A value of *p* < 0.05 was considered statistically significant. Differences between two groups were analyzed using two‐tailed unpaired Student's *t*‐test. Comparisons among multiple groups were analyzed by one‐way analysis of variance (ANOVA) followed by Tukey's multiple comparison test. For studies involving multiple groups across different time points, two‐way ANOVA followed by Tukey's multiple comparison test was applied, unless otherwise specified for individual time points. Semi‐quantitative histopathological scores were analyzed using the Kruskal–Wallis test followed by Dunn's multiple comparison test. Statistical significance is denoted as ^*^ *p* < 0.05, ^**^ *p* < 0.01, ^***^ *p* < 0.001, and ^****^ *p* < 0.0001; ns indicates not significant.

## Author Contributions

**Jie Miao**: conceptualization, methodology, writing – original draft, funding acquisition. **Jing Wang**: methodology, investigation, data curation, validation, writing – original draft. **Yankun Li**: software, data curation, methodology, validation. **Haipei Zhang**: methodology, validation, data curation. **Yanli Zhang**: methodology, validation, data curation, funding acquisition. **Qingnian Li**: methodology, validation, data curation. **Bo Xiao**: methodology, validation. **Wenhu Zhou**: conceptualization, methodology, writing – review and editing, supervision, funding acquisition. **Junhong Guo**: conceptualization, methodology, writing – review and editing, supervision, funding acquisition.

## Ethical Approval Statement

All animal experiments were conducted in accordance with protocols approved by the Animal Care and Use Committee of Central South University (Approval No.: CSU‐2024‐0398).

## Conflicts of Interest

The authors declare no conflicts of interest.

## Supporting information




**Supporting File**: advs77929‐sup‐0001‐SuppMat.pdf

## Data Availability

The data that supports the findings of this study are available in the supplementary material of this article.
