# Supplementary material for: Restores Aβ Clearance by Overcoming PCSK9–LRP1 Dysregulation and TRIB3‐Mediated Autophagy Blockade in Alzheimer's Disease
Source: Adv Sci (Weinh). 2026 Sep 29:e77929. Online ahead of print. doi: 10.1002/advs.77929 (PMC13624319; doi:10.1002/advs.77929)
Supplement: Supplementary file 1 [file ADVS-9999-e77929-s001.pdf]

Supporting Information

Restores Aβ Clearance by Overcoming PCSK9–LRP1 Dysregulation and TRIB3-Mediated Autophagy Blockade in Alzheimer's Disease

Jie Miao[1,2†], Jing Wang[3,4†], Yankun Li[5], Haipei Zhang[2], Yanli Zhang[6], Qingnian Li[5], Bo Xiao[3], Wenhu Zhou[5,7*], Junhong Guo[1,2*]

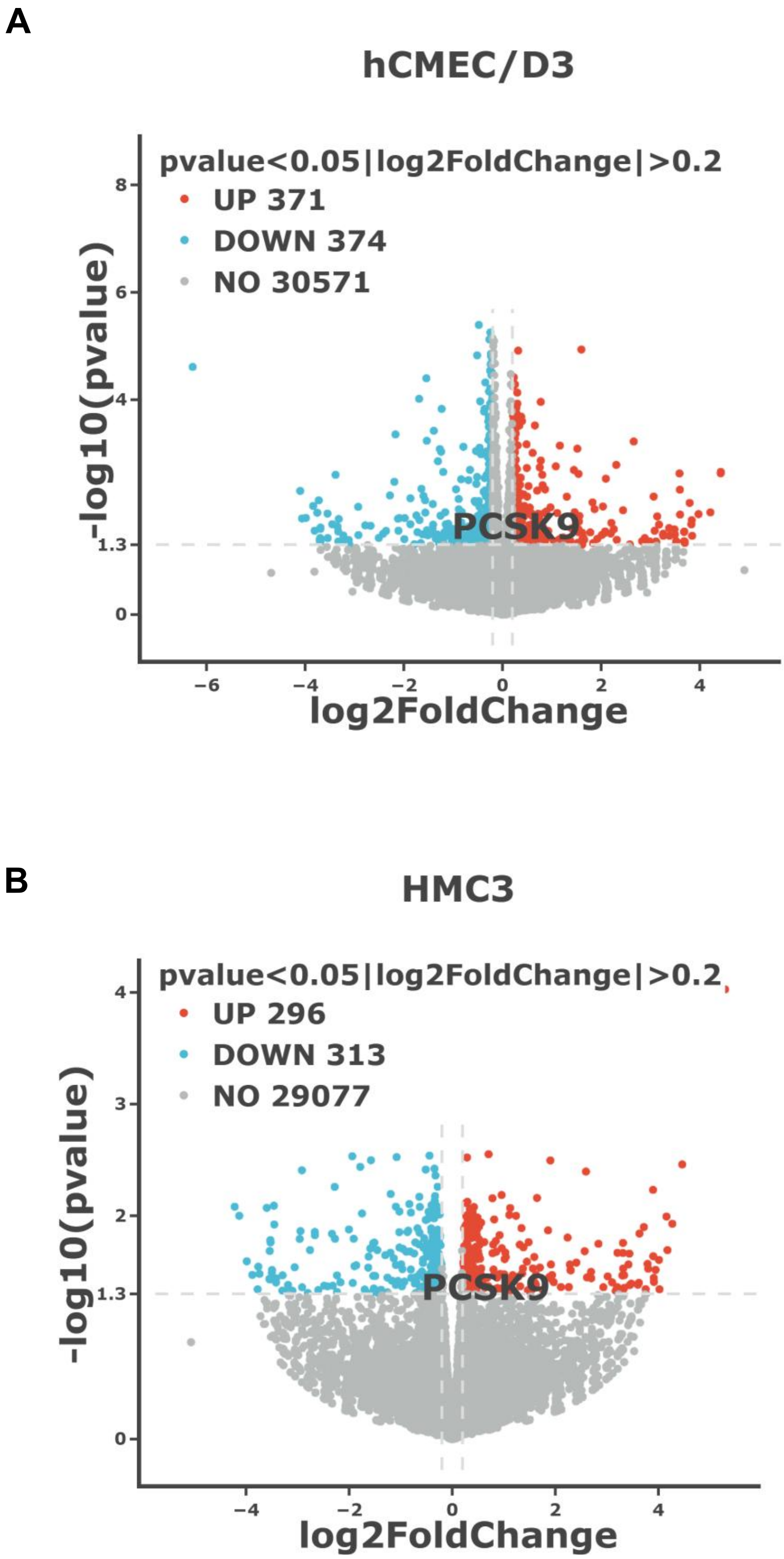

Figure S1. Transcriptome sequencing reveals significant upregulation of PCSK9 in Aβ-treated cells. Volcano plots showing differentially expressed genes (DEGs) in Aβ-treated

human brain endothelial cells (hCMEC/D3) (**A**) and human microglia (HMC3) (**B**). $n = 3$ biological replicates per group. The differential expression thresholds are indicated in the plots. Statistical significance for transcriptomic differential expression was determined using the differential expression analysis described in the Methods.

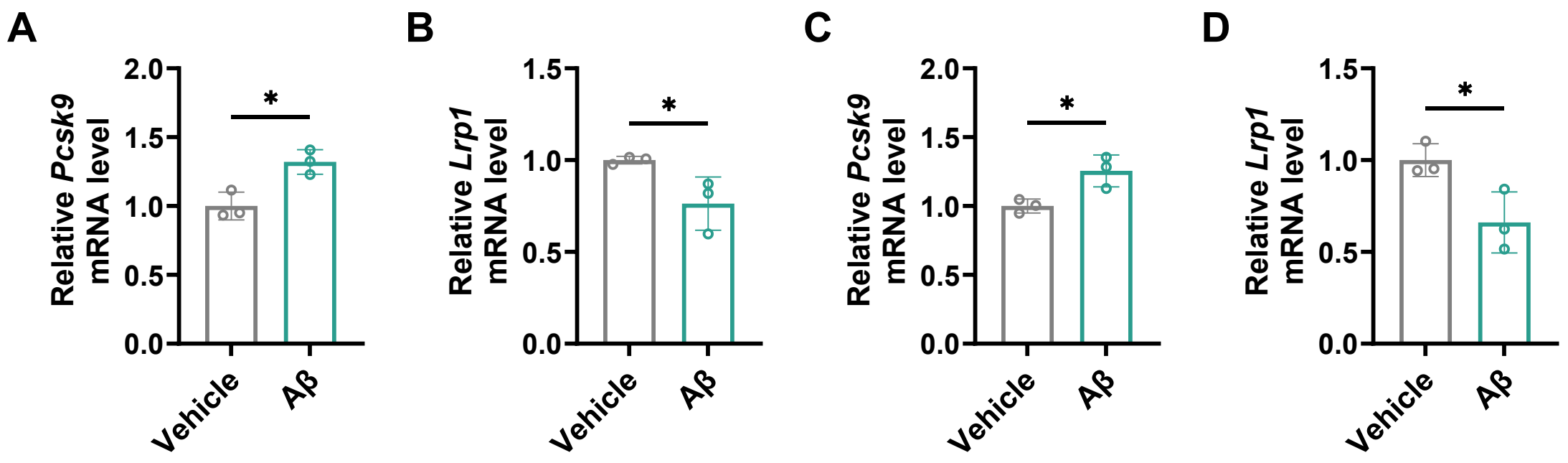

Figure S2. Effects of $A\beta_{1\text{–}42}$ on *Pcsk9* and *Lrp1* mRNA Expression. (A–B) Relative mRNA expression levels of *Pcsk9* (**A**) and *Lrp1* (**B**) in bEnd.3 cells treated with 20 µM recombinant human $A\beta_{1\text{–}42}$ for 24 h. Values were normalized to the mean value of the Vehicle group, which was set to 1. **(C and D)** Relative mRNA expression levels of *Pcsk9* (**C**) and *Lrp1* (**D**) in BV2 cells following 24 h exposure to 20 µM recombinant human $A\beta_{1\text{–}42}$. Values were normalized to the mean value of the Vehicle group, which was set to 1. Data are presented as mean ± SD (n = 3 independent experiments). Unpaired two-tailed Student's t-test for each panel. *$p < 0.05$, **$p < 0.01$, ***$p < 0.001$.

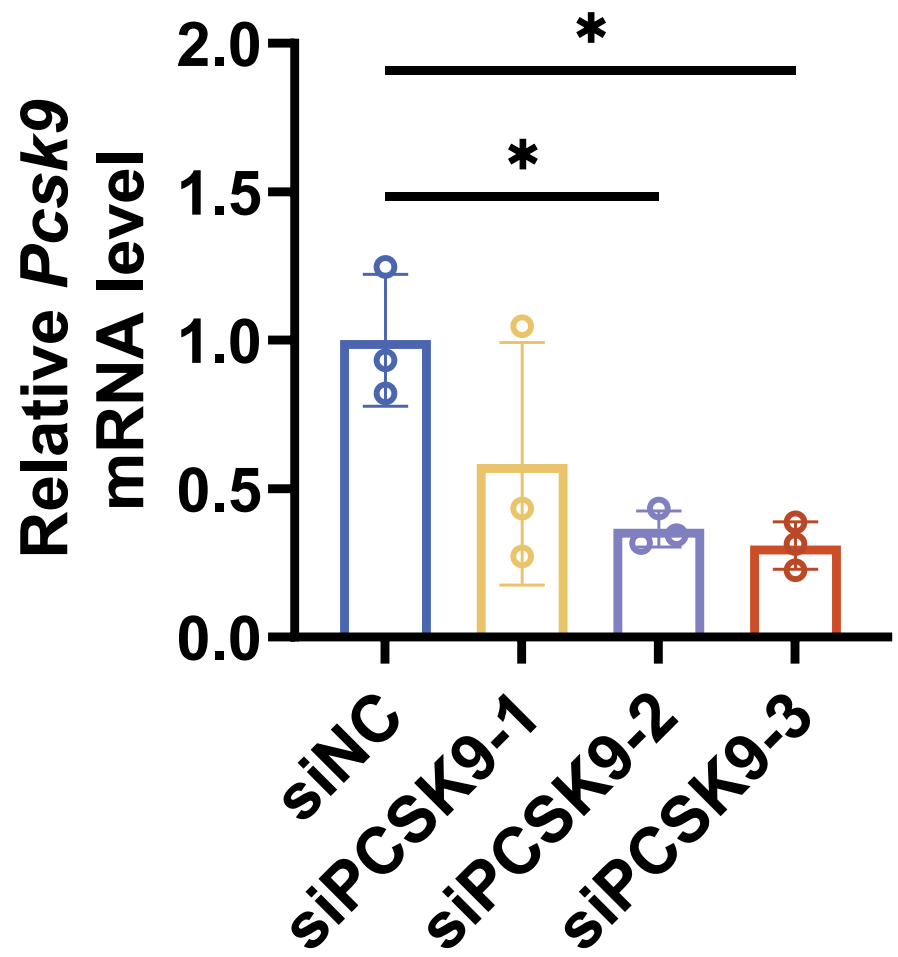

Figure S3. Screening of optimal siRNA for *Pcsk9* knockdown. Relative mRNA expression levels of *Pcsk9* in bEnd.3 cells after 24 h intervention with three different *Pcsk9*-targeting siRNAs (50 nM), demonstrating the selection of the most effective siRNA for gene silencing. Values were normalized to the mean value of the siNC group, which was set to 1. One-way ANOVA followed by Tukey's multiple-comparison test. *$p < 0.05$, **$p < 0.01$, ***$p < 0.001$.

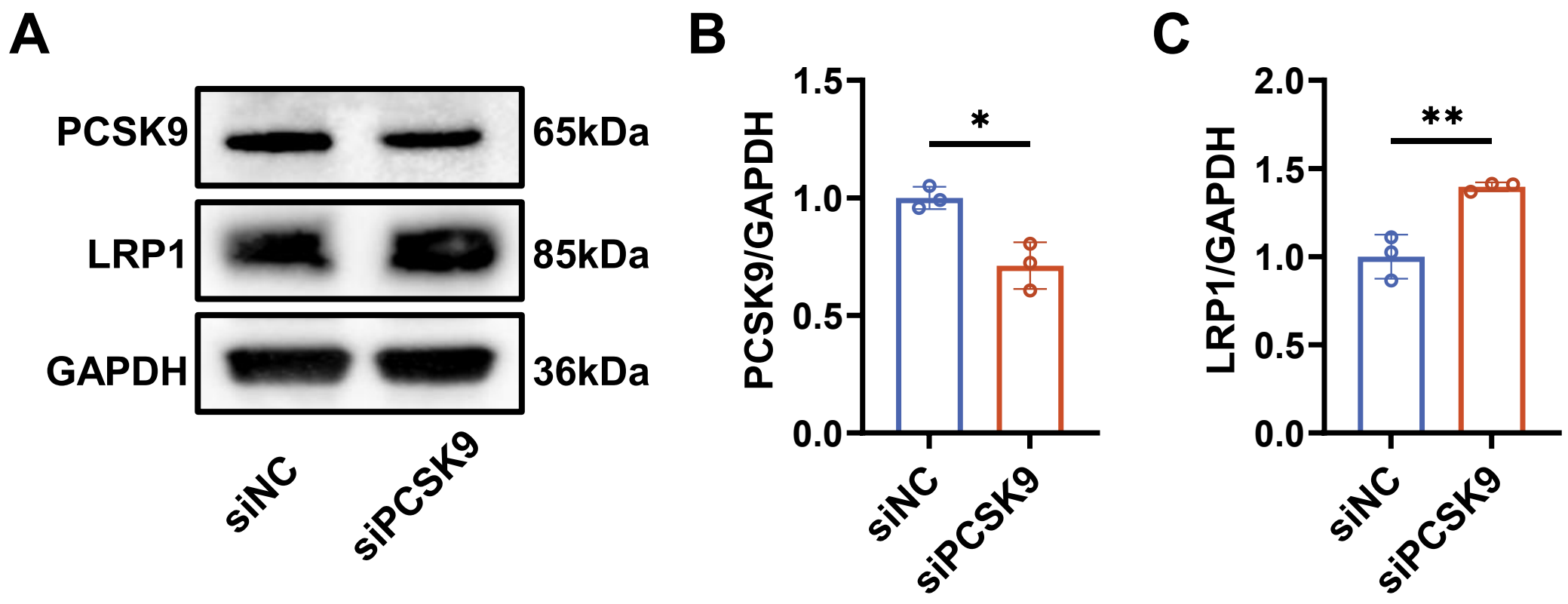

Figure S4. Effects of siPCSK9 on PCSK9 and LRP1 protein expression in bEnd.3 cells. Representative Western blot (WB) bands of PCSK9 and LRP1 in bEnd.3 cells after siPCSK9 treatment (**A**) and quantitative densitometry of PCSK9 (**B**) and LRP1 (**C**) protein levels. Values were normalized to the mean value of the siNC group, which was set to 1. Data are presented as mean ± SD (n = 3 independent experiments). Unpaired two-tailed Student's t-test for (B and C). *$p < 0.05$, **$p < 0.01$, ***$p < 0.001$.

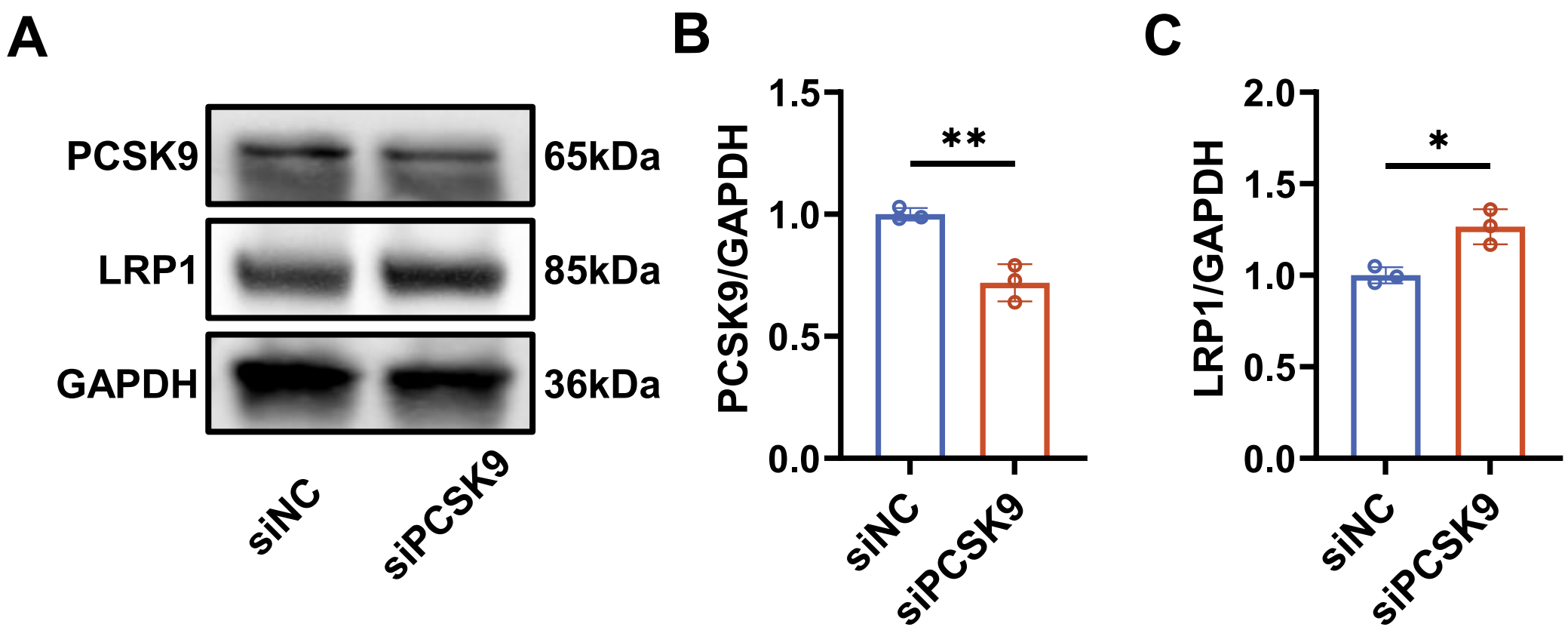

Figure S5. Effects of siPCSK9 on PCSK9 and LRP1 protein expression in BV2 cells. Representative WB bands of PCSK9 and LRP1 following siPCSK9 intervention in BV2 cells (**A**) and quantitative densitometry of PCSK9 (**B**) and LRP1 (**C**) protein levels. Values were normalized to the mean value of the siNC group, which was set to 1. Data are presented as mean ± SD (n = 3 independent experiments). Unpaired two-tailed Student's t-test for (B and C). *$p < 0.05$, **$p < 0.01$, ***$p < 0.001$.

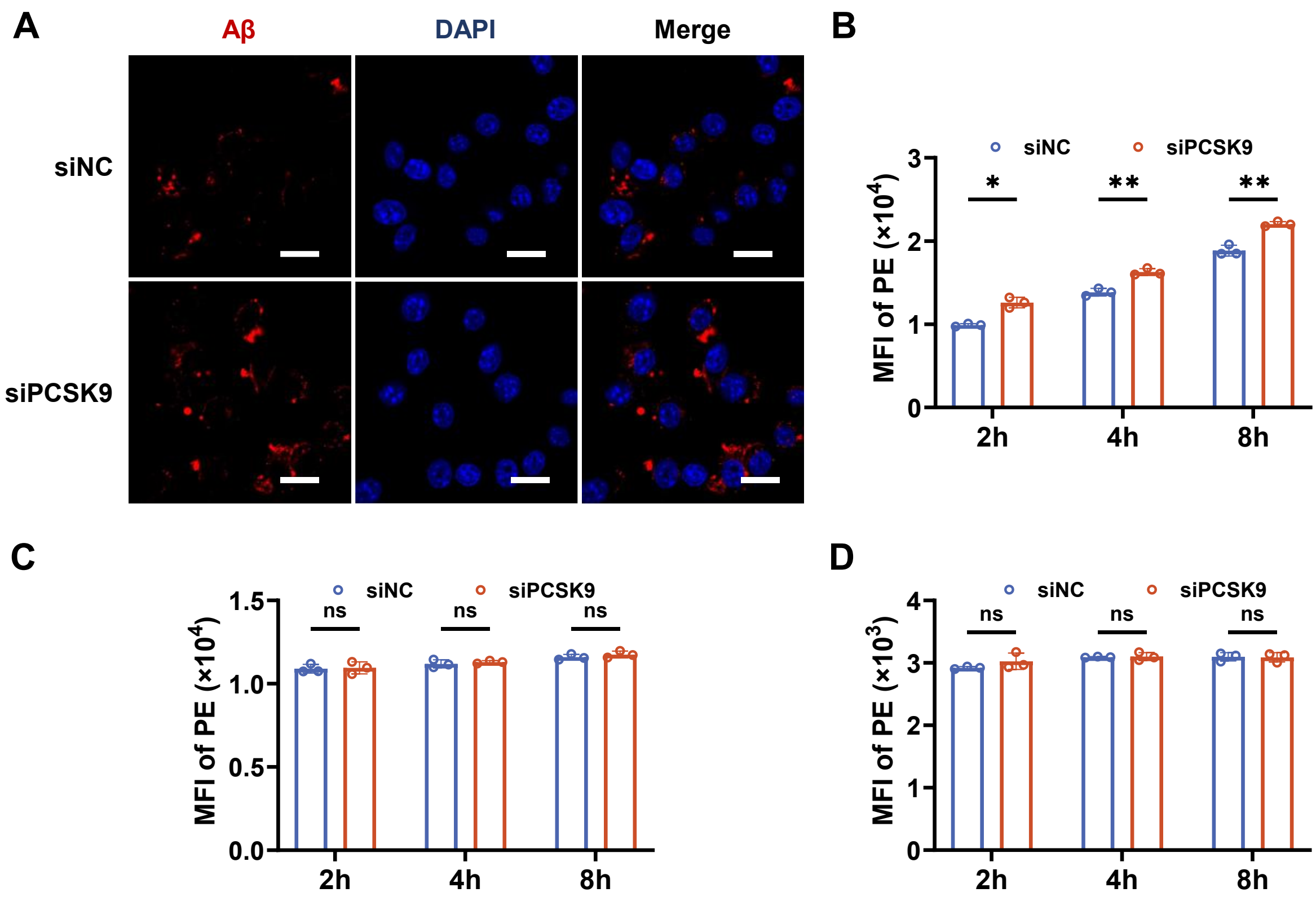

Figure S6. Effects of siPCSK9 on cellular uptake and nonspecific binding of Aβ$_{1-42}$. (A) Representative confocal microscopy images showing 5-TAMRA-Aβ$_{1-42}$ uptake (red) in siPCSK9-treated BV2 cells. Nuclei were stained with DAPI (blue). Scale bar = 20 μm. **(B)** Flow cytometry quantification of 5-TAMRA-Aβ$_{1-42}$ uptake at 37 °C in BV2 cells at 2, 4, and 8 h after siPCSK9 treatment. **(C** and **D)** Flow cytometry quantification of nonspecific binding of 5-TAMRA-Aβ$_{1-42}$ at 4 °C in siPCSK9-treated bEnd.3 cells (**C**) and BV2 cells (**D**) across various time points. Data are presented as mean ± SD (*n* = 3 independent experiments). Two-way ANOVA followed by Tukey's multiple-comparison test for (B–D). ns, not significant; *p < 0.05, **p < 0.01, ***p < 0.001.

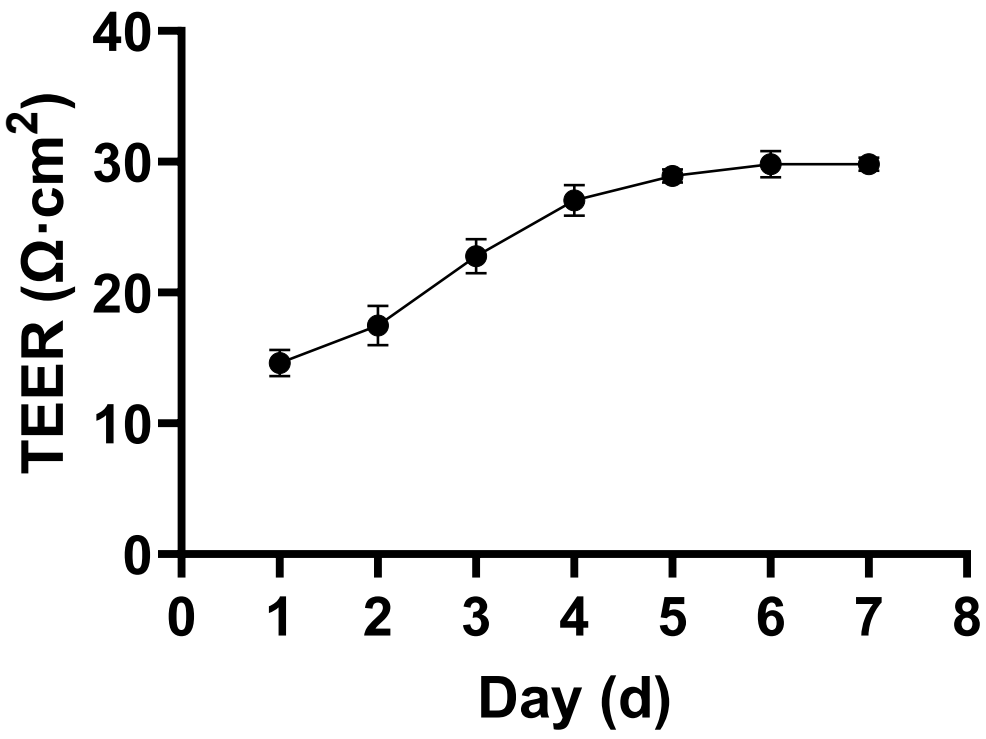

Figure S7. ***In vitro*** **blood-brain barrier (BBB) model establishment and functional characterization.** Changes in transendothelial electrical resistance (TEER) of bEnd.3 cell monolayers during the construction of the *in vitro* BBB model. TEER values stabilized at approximately 29.8 Ω·cm² by day 6 of culture. Data are presented as mean ± SD (n = 3 independent experiments).

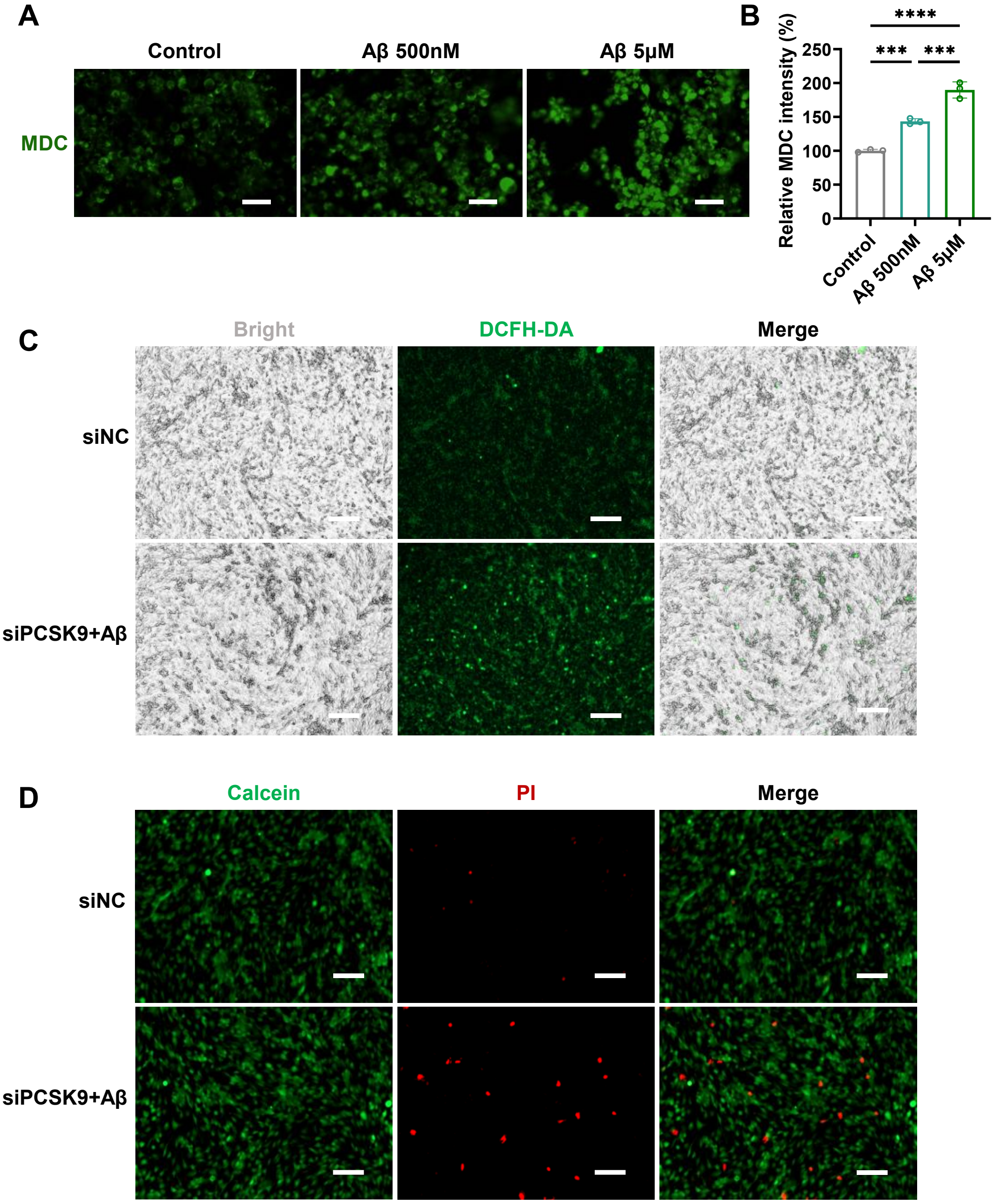

Figure S8. Analysis of autophagy, ROS levels, and cell viability in Aβ-treated bEnd.3 cells. **(A)** Representative fluorescence images showing autophagic vacuoles in Aβ-treated bEnd.3 cells stained with MDC. Scale bar = 50 μm. **(B)** Quantitative analysis of mean fluorescence intensity of MDC staining. Values were normalized to the mean value of the Control group, which was set to 100%. **(C)** Detection of intracellular ROS levels using DCFH-DA fluorescence in bEnd.3 cells treated with siNC or siPCSK9 followed by Aβ intervention. Scale bar = 200 μm. **(D)** Cell viability and toxicity assessed by Calcein-AM/PI double staining (green:

live cells; red: dead cells) in Aβ-treated bEnd.3 cells. Scale bar = 100 μm. Data in (B) are presented as mean ± SD (n = 3 independent experiments). one-way ANOVA followed by Tukey's multiple-comparison test for (B). *$p < 0.05$, **$p < 0.01$, ***$p < 0.001$, ****$p < 0.0001$.

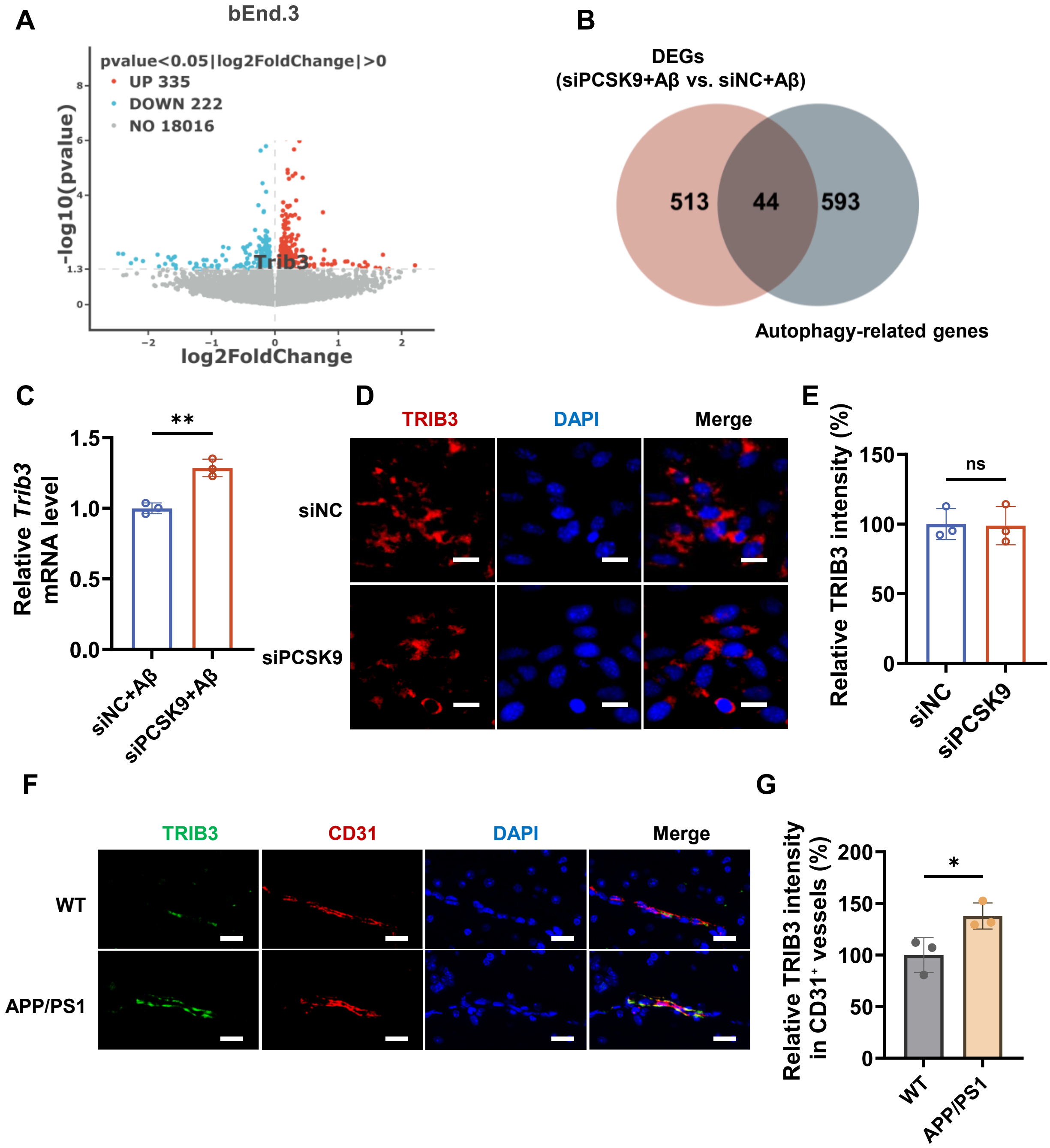

Figure S9. Transcriptomic profiling and validation of TRIB3 regulation under siPCSK9+Aβ treatment, basal PCSK9 silencing, and AD-like vascular conditions. (A) Volcano plot showing DEGs in bEnd.3 cells treated with siPCSK9 and Aβ. The differential expression thresholds are indicated in the plots. Statistical significance for transcriptomic differential expression was determined using the differential expression analysis described in

the Methods. **(B)** Venn diagram illustrating the overlap between the identified DEGs (siPCSK9+Aβ vs. siNC+Aβ) and known autophagy-related genes **(C)** Relative mRNA expression level of *Trib3* in bEnd.3 cells after intervention with siPCSK9 and Aβ. Values were normalized to the mean value of the siNC+Aβ group, which was set to 1. (**D** and **E**) Representative immunofluorescence images (**D**) and quantification (**E**) of TRIB3 fluorescence intensity in bEnd.3 cells after 24 h treatment with siNC or siPCSK9 under basal conditions without exogenous Aβ. Nuclei were stained with DAPI. Scale bar = 20 μm. Relative TRIB3 intensity in (**E**) was normalized to the mean value of the siNC group, which was set to 100%. (**F** and **G**) Representative immunofluorescence images (**F**) and quantification (**G**) of TRIB3 fluorescence in $CD31^+$ cerebrovascular structures in brain sections from 6-month-old APP/PS1 mice and WT littermate controls. Nuclei were stained with DAPI. TRIB3 mean fluorescence intensity (MFI) was quantified within $CD31^+$ cerebrovascular ROIs and normalized to the mean value of the WT group, which was set to 100%. Scale bar = 20 μm. Data are presented as mean ± SD. For cell experiments, $n = 3$ independent experiments. For tissue immunofluorescence, six $CD31^+$ vascular ROI measurements were averaged within each mouse, and mouse-level means were used as biological replicates for statistics; $n = 3$ mice per group. Unpaired two-tailed Student's t-test for two-group comparisons in (C), (E), and (G). ns, not significant; $*p < 0.05$, $**p < 0.01$, $***p < 0.001$, $****p < 0.0001$.

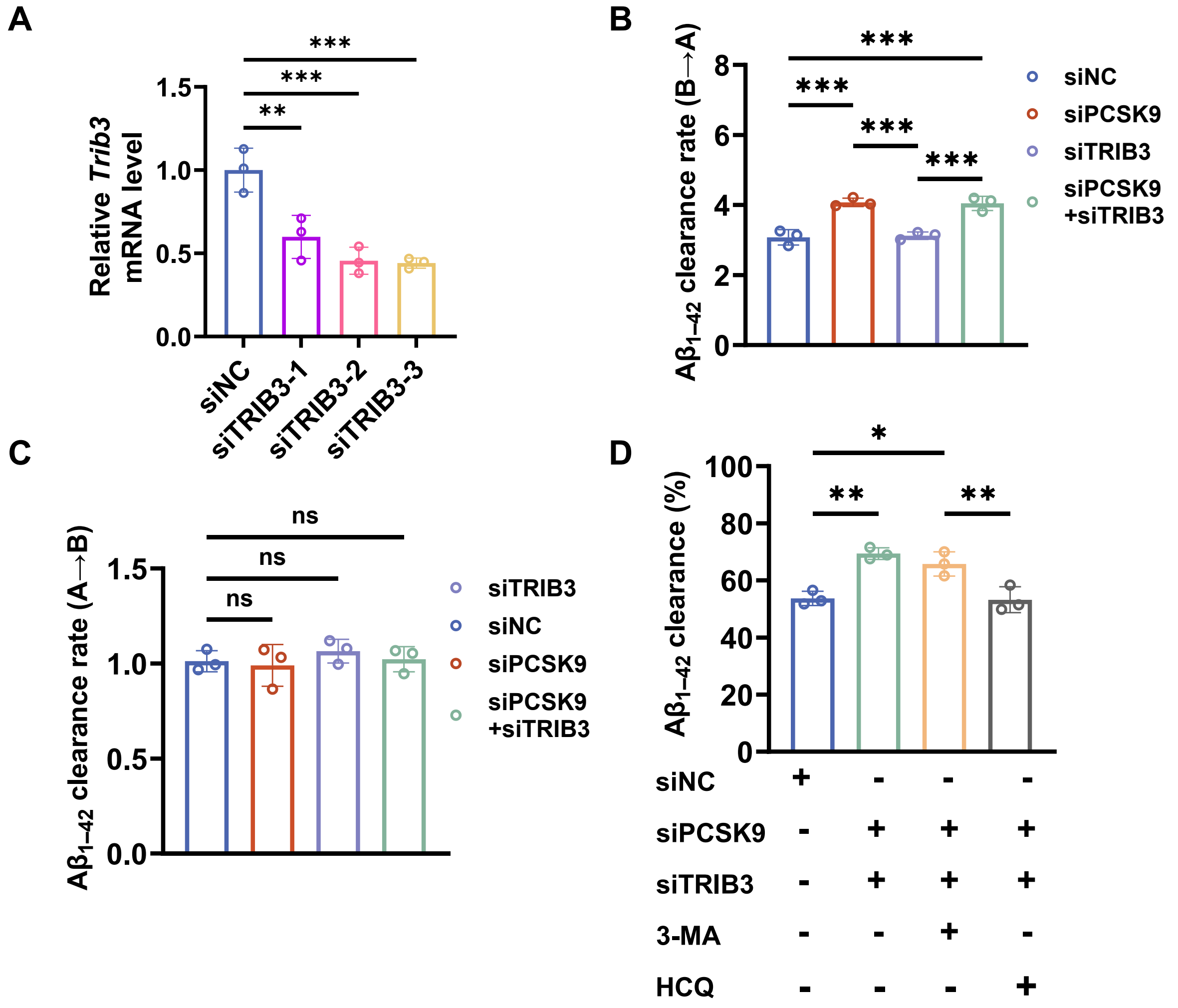

Figure S10. Validation of siTRIB3 knockdown and functional comparison of PCSK9/TRIB3 silencing in Aβ transport and intracellular degradation. (**A**) Screening of the optimal siRNA for *Trib3* knockdown. Relative mRNA expression levels of *Trib3* in bEnd.3 cells after treatment with three different *Trib3*-targeting siRNAs (50 nM) for 24 h. Values were normalized to the mean value of the siNC group, which was set to 1. (**B** and **C**) Bidirectional $A\beta_{1-42}$ transport assays across bEnd.3 Transwell monolayers after siNC, siPCSK9, siTRIB3, or siPCSK9+siTRIB3 treatment. B→A denotes basolateral/brain-to-apical/blood Aβ transport (**B**), and A→B denotes apical/blood-to-basolateral/brain Aβ transport (**C**). (**D**) Intracellular $A\beta_{1-42}$ degradation after siNC or siPCSK9+siTRIB3 treatment with or without autophagy inhibitors 3-methyladenine (3-MA) or hydroxychloroquine (HCQ). Data are presented as mean ± SD (*n*

= 3 independent experiments). One-way ANOVA followed by Tukey's multiple-comparison test for (A-D). ns, not significant; *$p < 0.05$, **$p < 0.01$, ***$p < 0.001$, ****$p < 0.0001$.

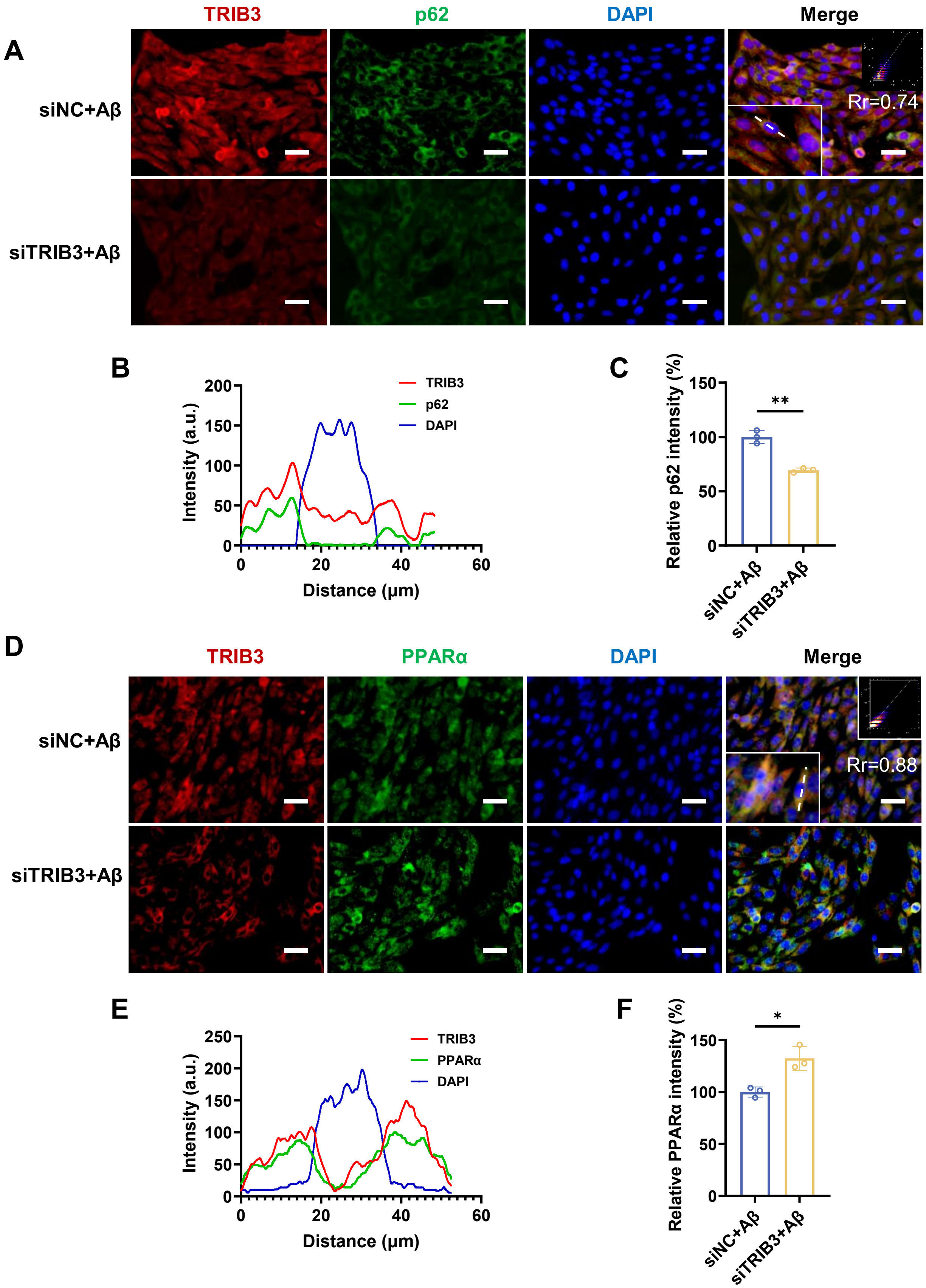

A
TRIB3
p62
DAPI
Merge
siNC+Aβ
siTRIB3+Aβ
Rr=0.74
B
TRIB3
p62
DAPI
Intensity (a.u.)
Distance (μm)
C
Relative p62 intensity (%)
**
siNC+Aβ
siTRIB3+Aβ
D
TRIB3
PPARα
DAPI
Merge
siNC+Aβ
siTRIB3+Aβ
Rr=0.88
E
TRIB3
PPARα
DAPI
Intensity (a.u.)
Distance (μm)
F
Relative PPARα intensity (%)
*
siNC+Aβ
siTRIB3+Aβ

Figure S11. Co-localization analysis of TRIB3 with p62 and PPARα in bEnd.3 cells following siTRIB3 and Aβ treatment. (**A–C**) Analysis of TRIB3 and p62 co-localization. Representative immunofluorescence images (**A**; red: TRIB3, green: p62, blue: nuclei), Pearson's correlation coefficient, enlarged view, fluorescence intensity profile along the dashed line (**B**), and quantitative analysis of p62 fluorescence intensity (**C**). Relative p62 fluorescence intensity was normalized to the mean value of the siNC+Aβ group, which was set to 100%. (**D–F**) Analysis of TRIB3 and PPARα co-localization. Representative immunofluorescence images (**D**; red: TRIB3, green: PPARα, blue: nuclei), Pearson's correlation coefficient, enlarged view, fluorescence intensity profile along the dashed line (**E**), and quantitative analysis of PPARα fluorescence intensity (**F**). Relative PPARα fluorescence intensity was normalized to the mean value of the siNC+Aβ group, which was set to 100%. Scale bar = 50 µm. Data are presented as mean ± SD (n = 3 independent experiments). Unpaired two-tailed Student's t-test for (C and F). *$p < 0.05$, **$p < 0.01$.

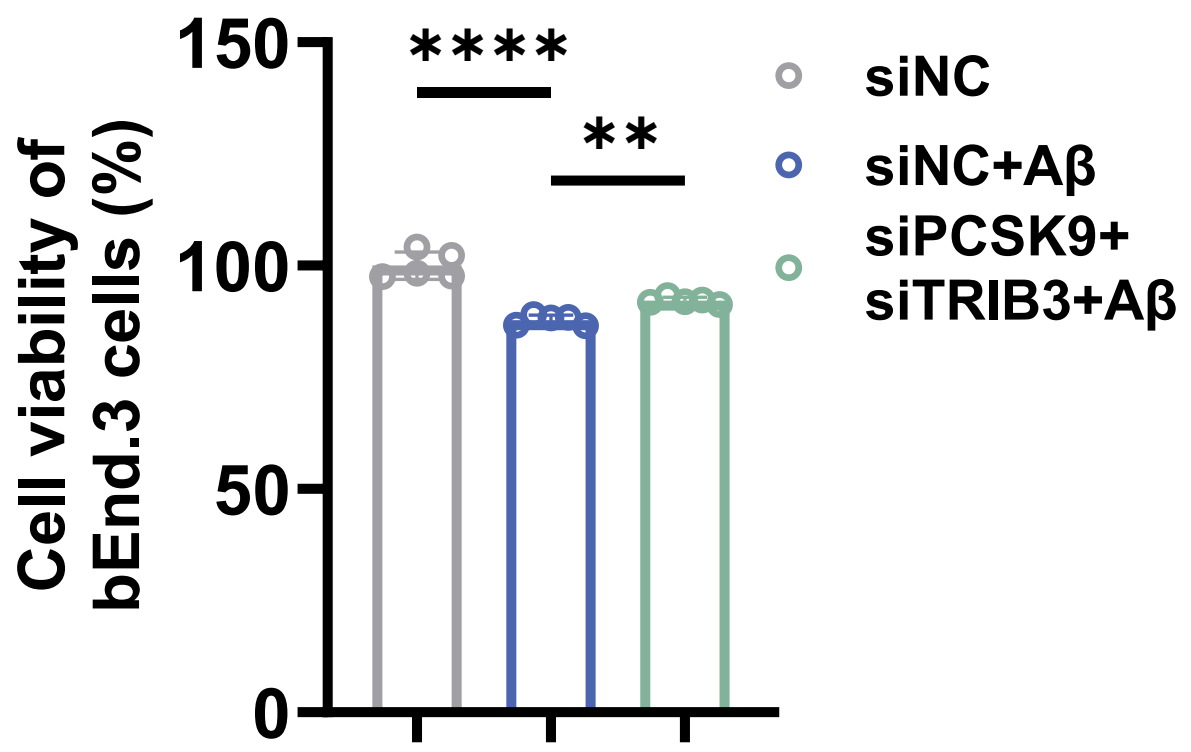

Figure S12. bEnd.3 cell viability after Aβ exposure with combined siPCSK9 and siTRIB3 intervention. Cell viability of bEnd.3 cells treated with 20 μM Aβ for 24 h after siPCSK9+siTRIB3 intervention, as measured by CCK-8 assay. Values were normalized to the mean value of the siNC group, which was set to 100%. Data are presented as mean ± SD (n = 5 independent experiments). One-way ANOVA followed by Tukey's multiple-comparison test. **$p < 0.01$, ****$p < 0.0001$.

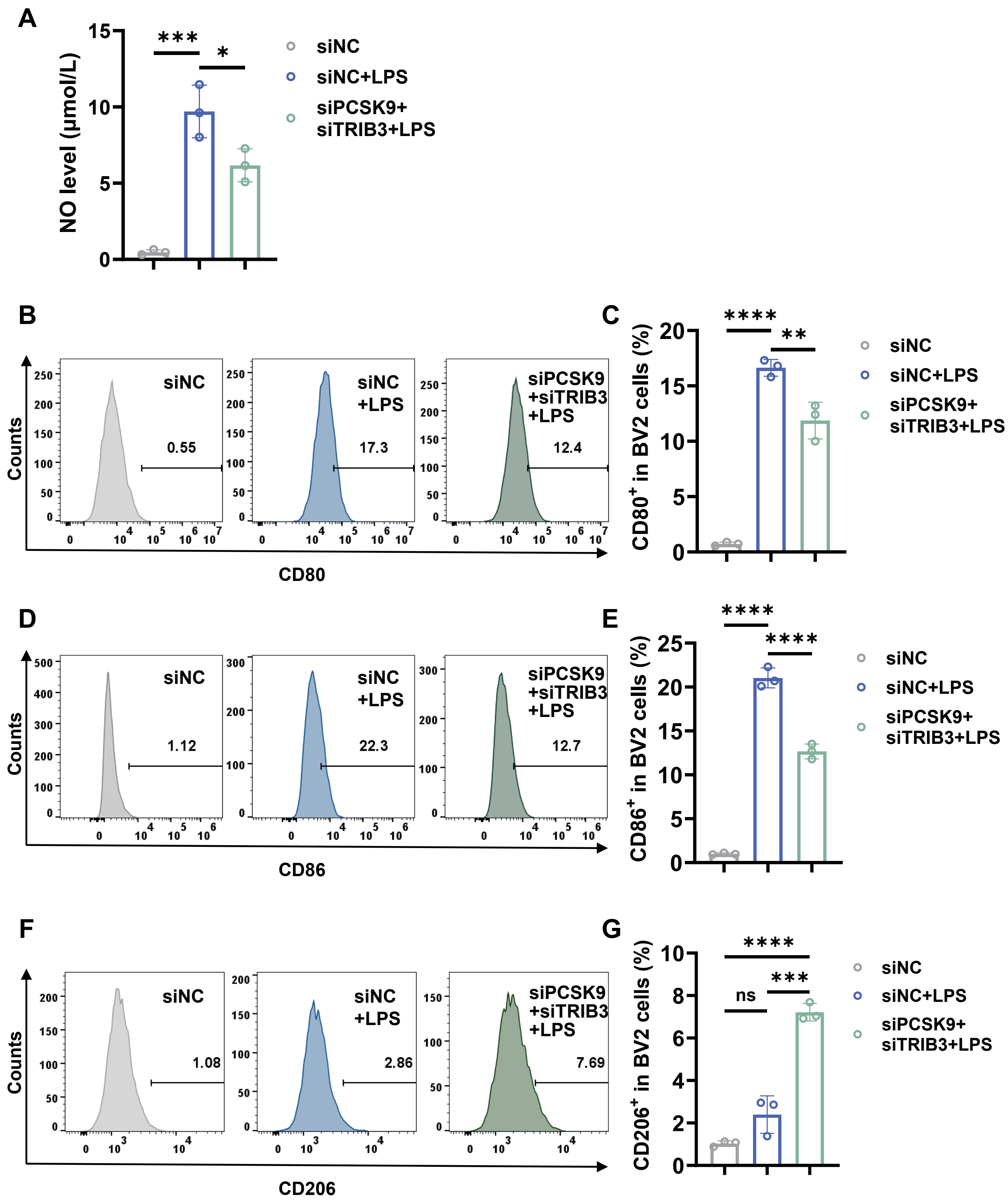

Figure S13. Nitric oxide production and polarization marker analysis in BV2 cells following LPS stimulation and combined siPCSK9/siTRIB3 intervention. (**A**) Nitric oxide (NO) levels in the supernatant of BV2 cells after LPS stimulation with or without combined siPCSK9+siTRIB3 intervention, measured using the Griess reagent assay. (**B** and **C**)

Representative flow cytometry plots (**B**) and quantitative analysis (**C**) of $CD80^+$ BV2 cells. (**D** and **E**) Representative flow cytometry plots (**D**) and quantitative analysis (**E**) of $CD86^+$ BV2 cells. (**F** and **G**) Representative flow cytometry plots (**F**) and quantitative analysis (**G**) of $CD206^+$ BV2 cells. Data are presented as mean ± SD (n = 3 independent experiments). One-way ANOVA followed by Tukey's multiple-comparison test. ns, not significant; *$p < 0.05$, **$p < 0.01$, ***$p < 0.001$, ****$p < 0.0001$.

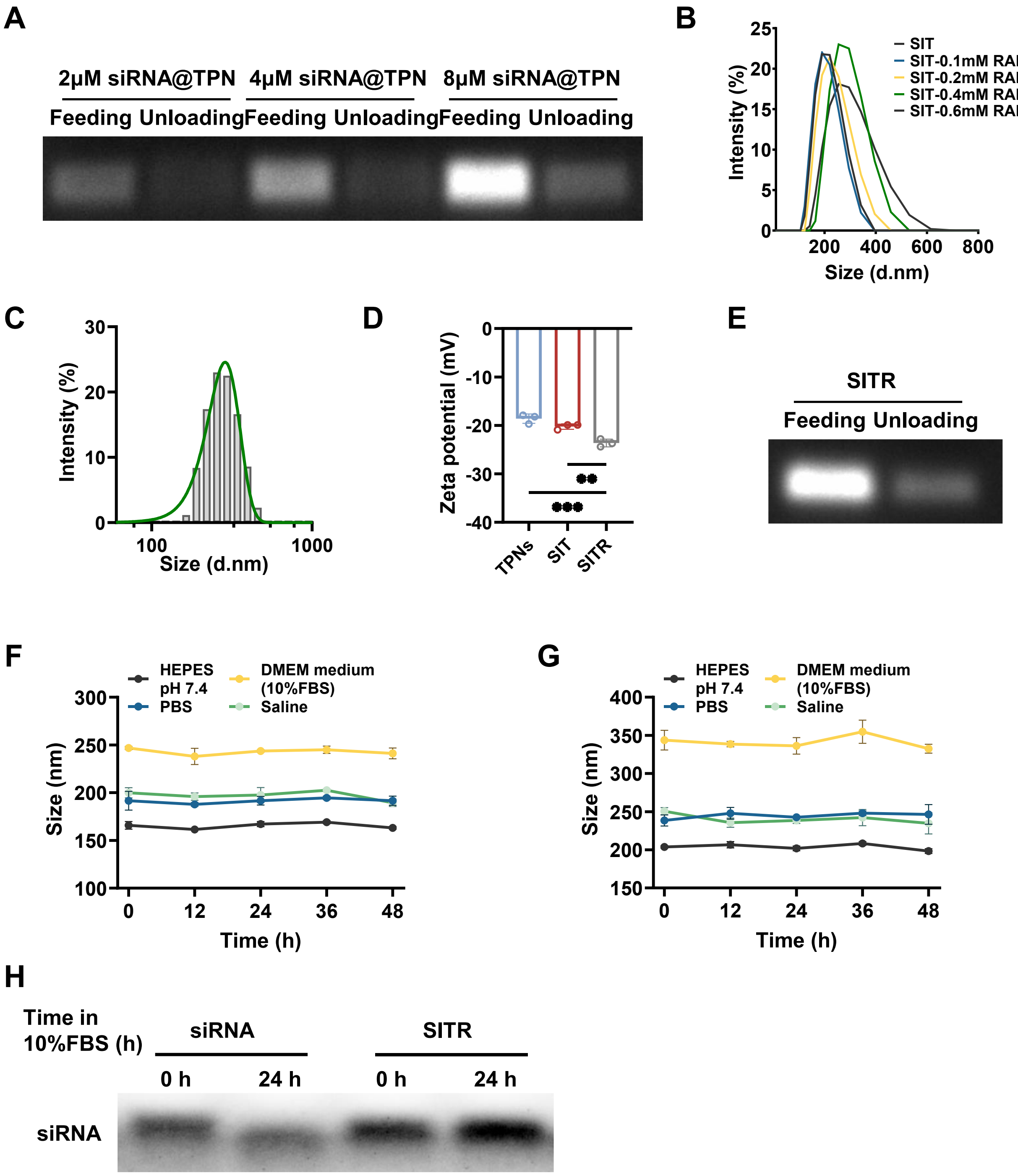

Figure S14. Characterization and stability evaluation of SIT and SITR. (A) Agarose gel electrophoresis of SIT complexes prepared with various FAM-siRNA feeding concentrations. Lanes: Feeding (free siRNA input), Unloading (supernatant containing unencapsulated siRNA). **(B)** Hydrodynamic size distribution of SITR modified with different RAP feeding concentrations. **(C)** Optimized hydrodynamic size profile of SITR (peak diameter: 256.6 ± 6.7

nm). **(D)** Zeta potential comparison of TPNs, SIT, and SITR. **(E)** Electrophoresis analysis confirming high siRNA encapsulation efficiency in SITR. **(F** and **G)** Colloidal stability of SIT (**F**) and SITR (**G**) in various physiological buffers (HEPES pH 7.4, DMEM with 10% FBS, PBS, saline) over 48 h. **(H)** SITR protects siRNA from serum degradation: Electrophoresis image after 24 h incubation in 10% FBS. Data are presented as mean ± SD (n = 3 independently prepared nanoparticle batches). One-way ANOVA followed by Tukey's multiple-comparison test for (D). *$p < 0.05$, **$p < 0.01$, ***$p < 0.001$.

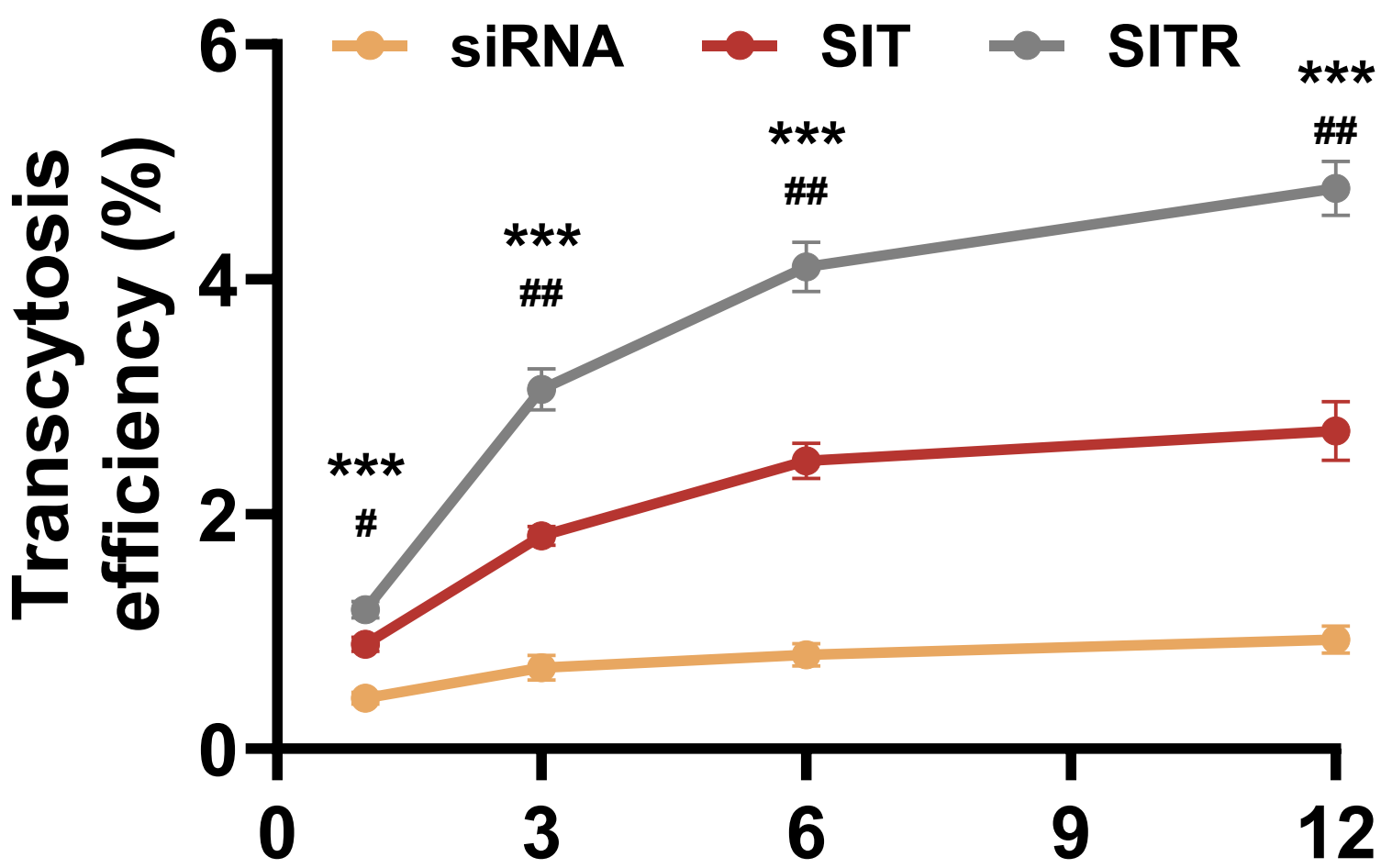

Figure S15. Comparison of Transcytosis Efficiency across different formulations. Quantitative comparison of the transcytosis efficiency of free siRNA, SIT, and SITR across bEnd.3 Transwell monolayers over time. Data are presented as mean ± SD (n = 3 independent experiments).Two-way ANOVA followed by Tukey's multiple-comparisons test. ***$p < 0.001$ indicate SITR versus free siRNA at the same time point; and #$p < 0.05$, ##$p < 0.01$ indicate SITR versus SIT at the same time point.

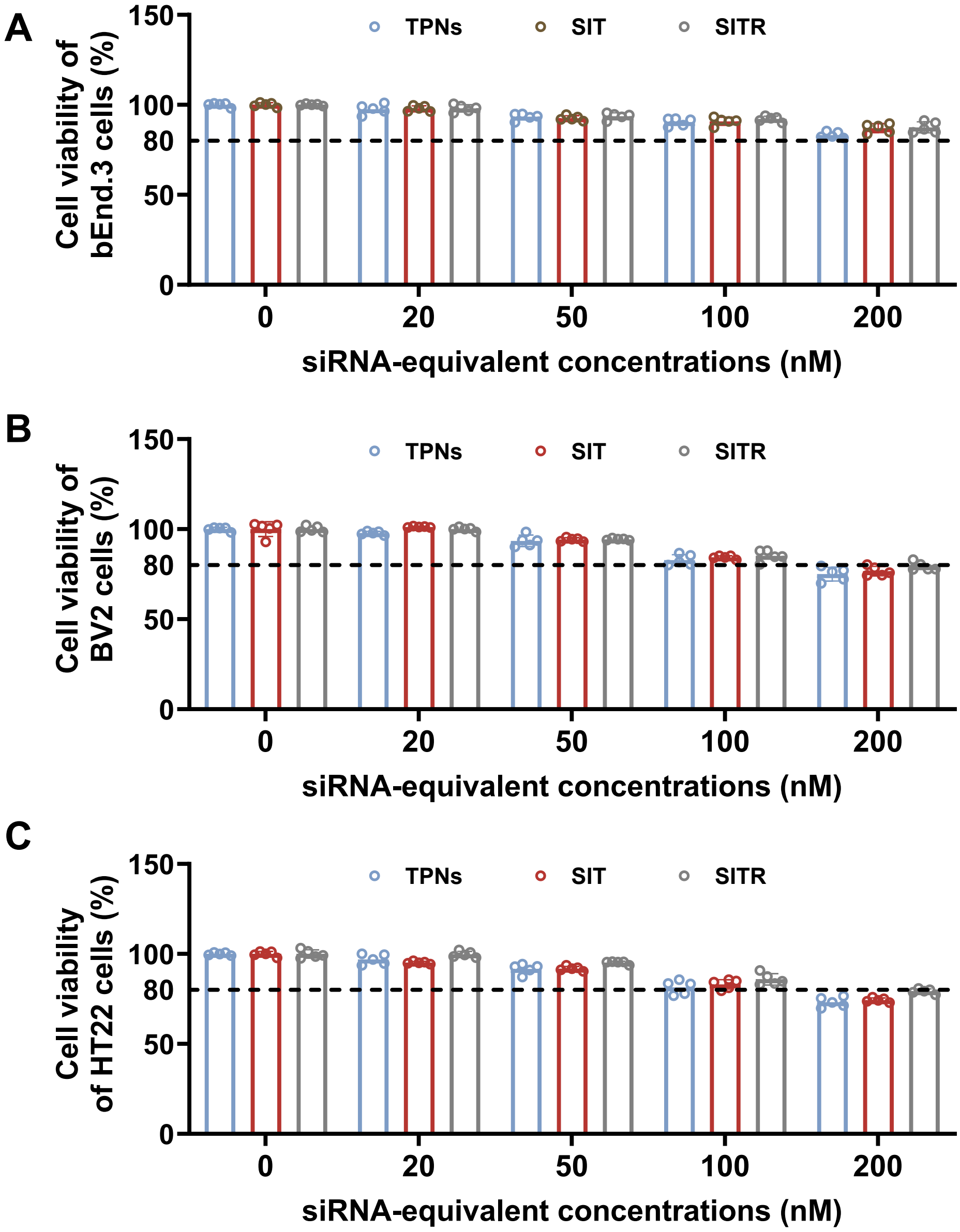

Figure S16. Cytotoxicity evaluation of nanoparticles in different neural cell lines. Viability of bEnd.3 cells (**A**), BV2 cells (**B**), and HT22 cells (**C**) after 24 h treatment with different siRNA-equivalent concentrations of TPN, SIT, and SITR, as measured by CCK-8 assay. The dashed line indicates 80% cell viability. Data are presented as mean ± SD (n = 5 independent experiments).

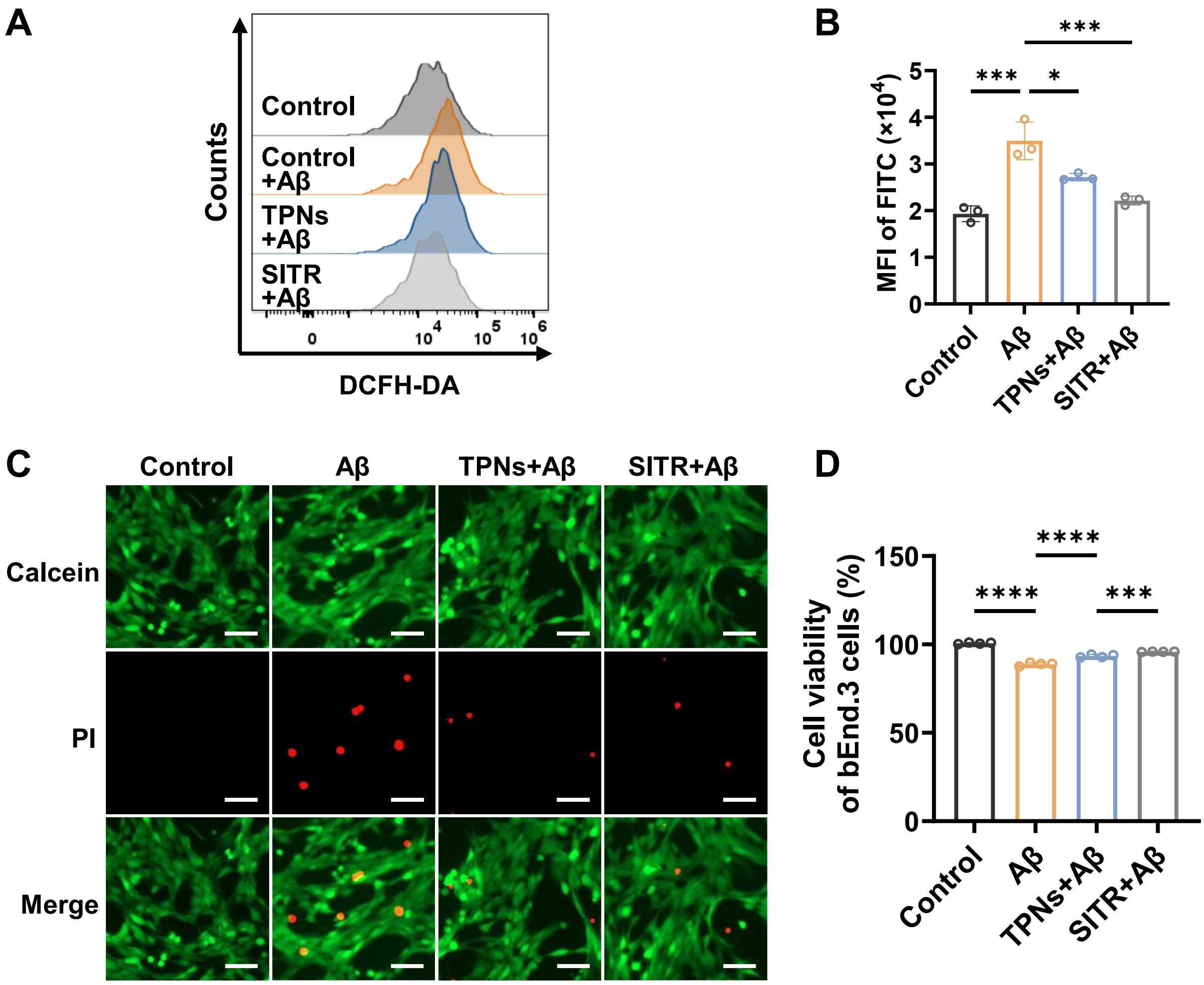

Figure S17. Effects of different formulations on Aβ-treated bEnd.3 cells. (A and **B)** ROS levels analyzed by flow cytometry using DCFH-DA: representative histograms (**A**) and quantitative analysis of MFI (**B**). **(C)** Live/Dead cell staining images after 24 h treatment (green: Calcein-AM, live cells; red: PI, dead cells). Scale bar = 50 μm. **(D)** Cell viability measured by CCK-8 assay after pretreatment with Aβ for 24 h followed by treatment with different formulations for 24 h. Values were normalized to the mean value of the Control group, which was set to 100%. Data are presented as mean ± SD. For (B), n = 3 independent experiments. For (D), n = 5 independent experiments. One-way ANOVA followed by Tukey's multiple-comparison test for (B and D). *$p < 0.05$, **$p < 0.01$, ***$p < 0.001$, ****$p < 0.0001$.

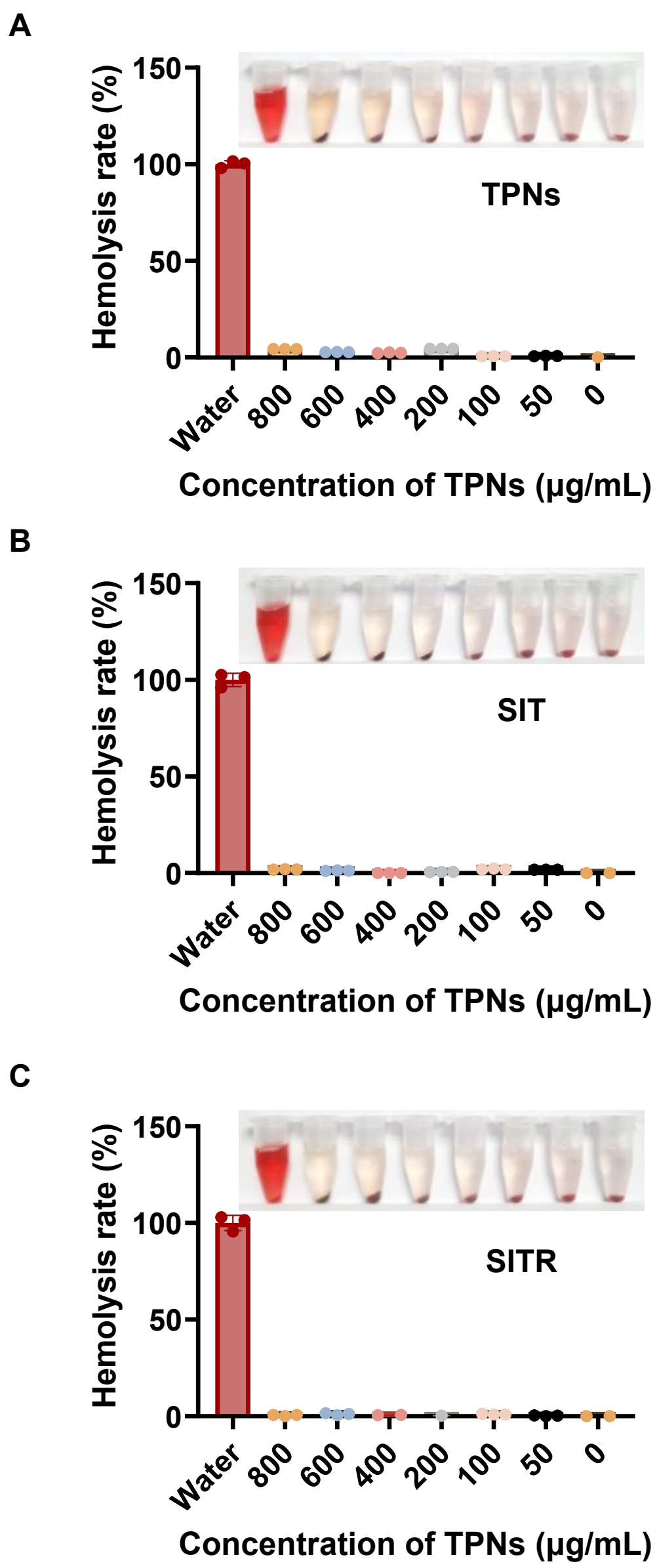

Figure S18. Hemocompatibility assessment of nanoparticles. Hemolysis assay of TPNs (**A**), SIT (**B**), and SITR (**C**) at various concentrations. Data are presented as mean ± SD (n = 3 independent experiments).

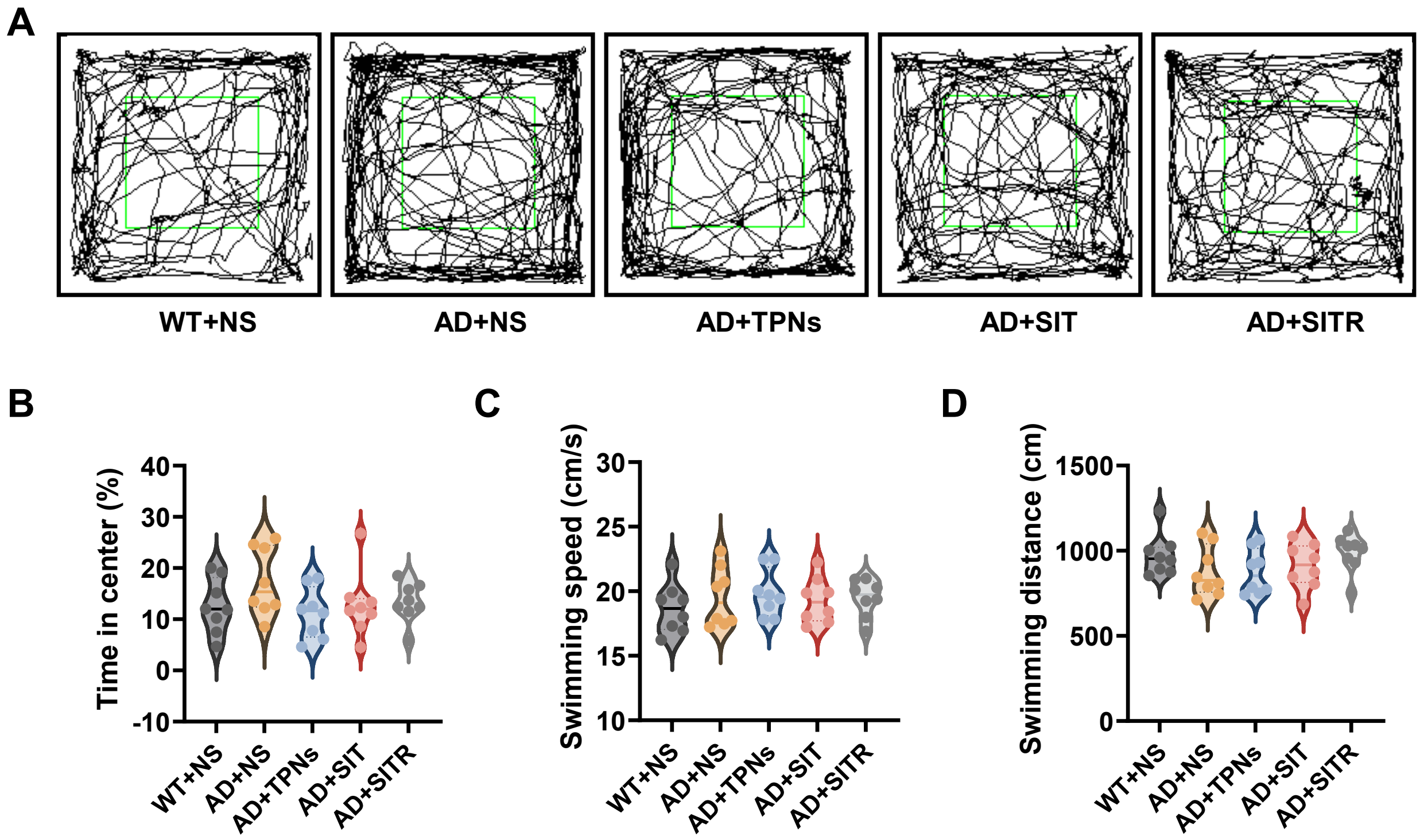

Figure S19. Behavioral assessments of mice in the open field and Morris water maze tests. **(A)** Representative movement trajectories of mice from different groups in the open field test. **(B)** Quantitative analysis of the percentage of time spent in the center zone of the open field (n = 8 mice per group). **(C and D)** Swimming speed (C) and total swimming distance (D) during the probe trial of the Morris water maze test on day 6 (n = 8 mice per group). Data are presented as mean ± SD. One-way ANOVA followed by Tukey's multiple-comparison test for (B–D). ns, not significant; *$p < 0.05$, **$p < 0.01$, ***$p < 0.001$.

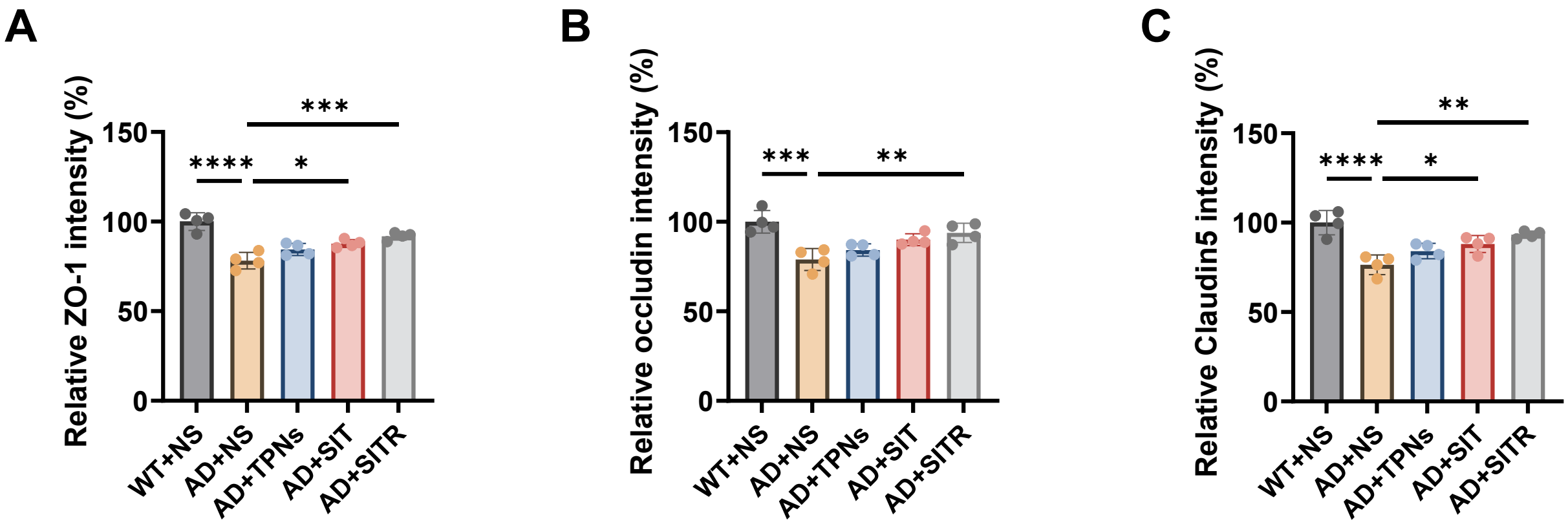

Figure S20. Statistical analysis of MFI for tight junction molecules in mouse brain tissue. Quantitative analysis of the MFI of ZO-1 (**A**), Occludin (**B**), and Claudin 5 (**C**) in cortical vasculature. Relative fluorescence intensity was normalized to the mean value of the WT+NS group, which was set to 100%. Data are presented as mean ± SD (n = 4 mice per group). One-way ANOVA followed by Tukey's multiple-comparison test. *$p < 0.05$, **$p < 0.01$, ***$p < 0.001$, ****$p < 0.0001$.

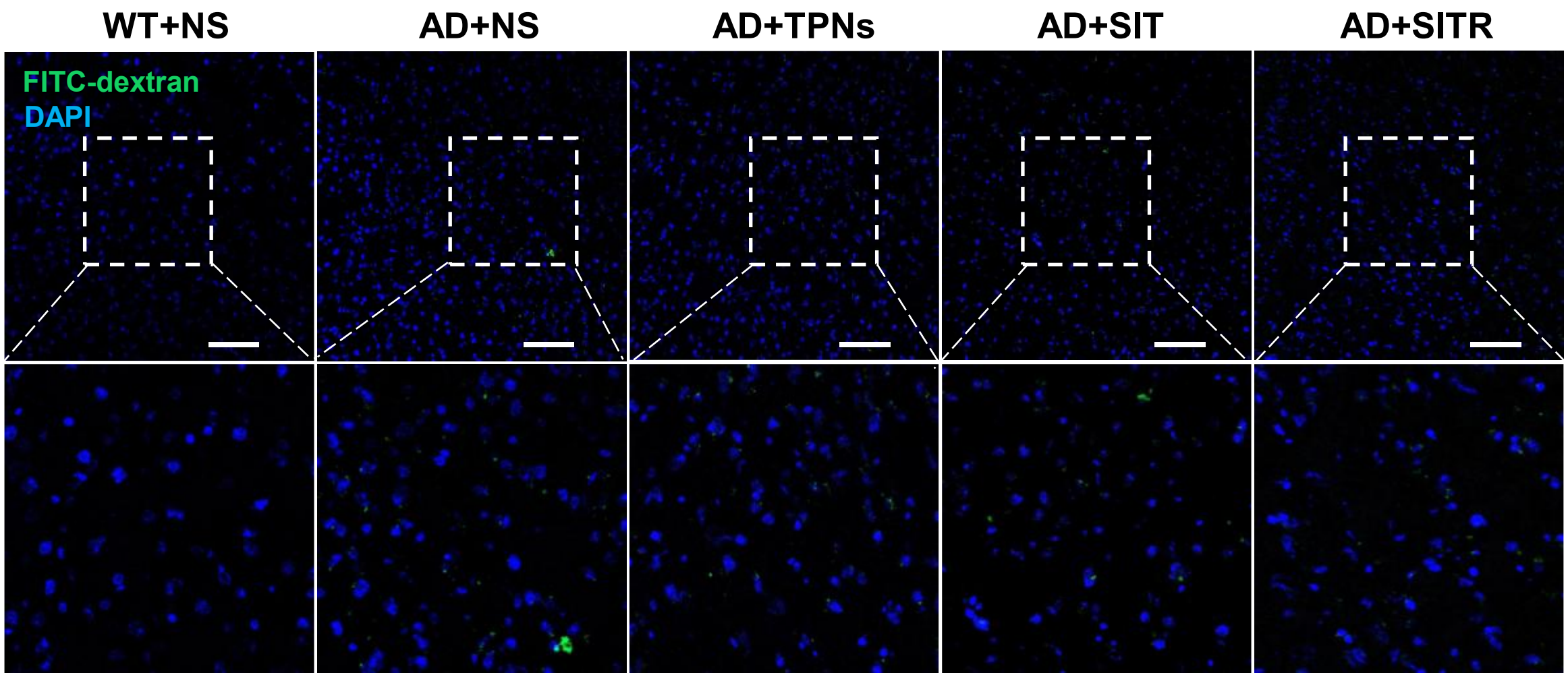

Figure S21. Evaluation of BBB permeability. Representative fluorescence images showing the extravasation of FITC-dextran (40 kDa) in the brain tissues of mice from different treatment groups. DAPI, blue; FITC-dextran, green. Scale bar = 100 μm.

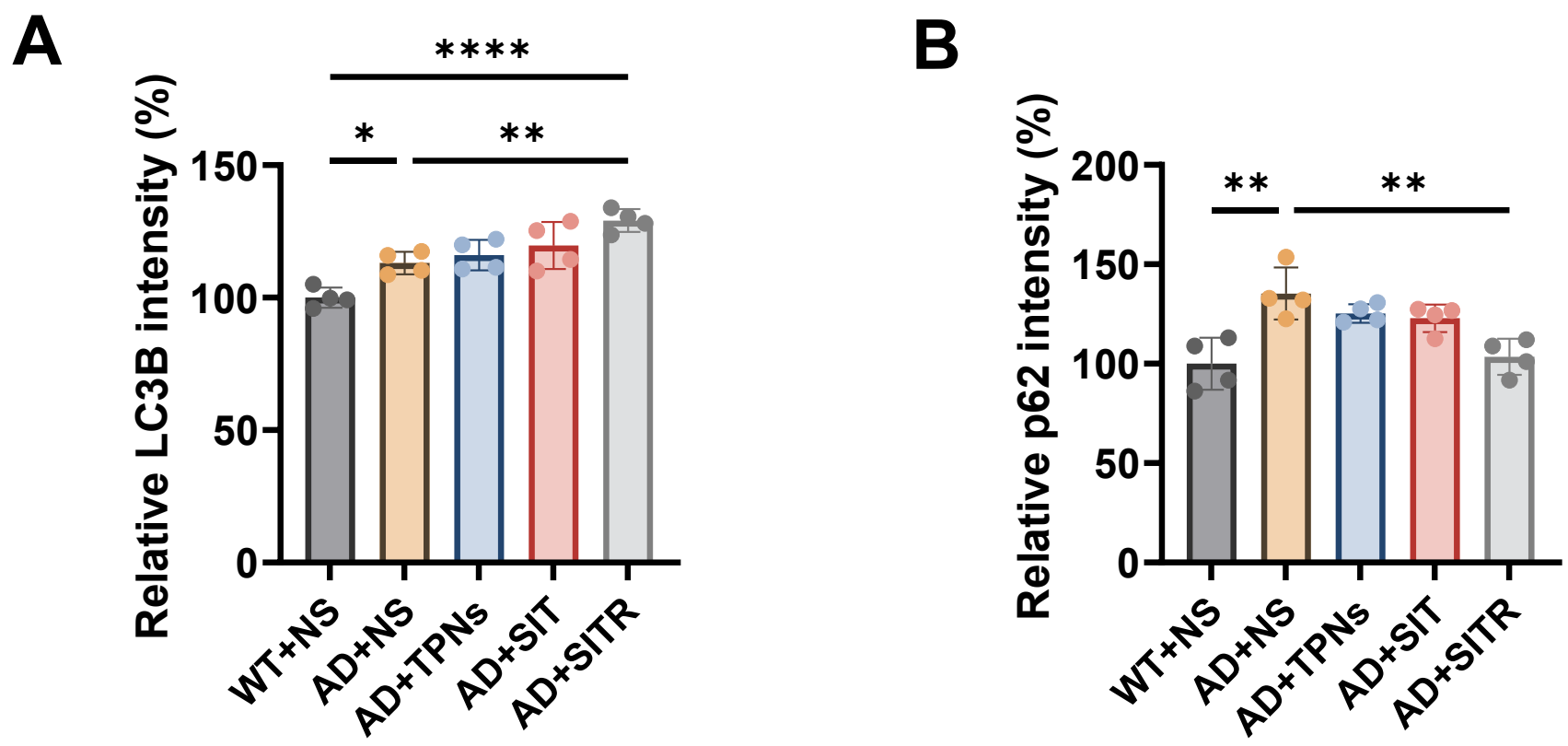

Figure S22. Quantitative analysis of autophagy markers in mouse brain tissue. Statistical analysis of the MFI of LC3B (**A**) and p62 (**B**) immunofluorescence in cortical tissue. Relative fluorescence intensity was normalized to the mean value of the WT+NS group, which was set to 100%. Data are presented as mean ± SD (n = 4 mice per group). One-way ANOVA followed by Tukey's multiple-comparison test. *$p < 0.05$, **$p < 0.01$, ***$p < 0.001$, ****$p < 0.0001$.

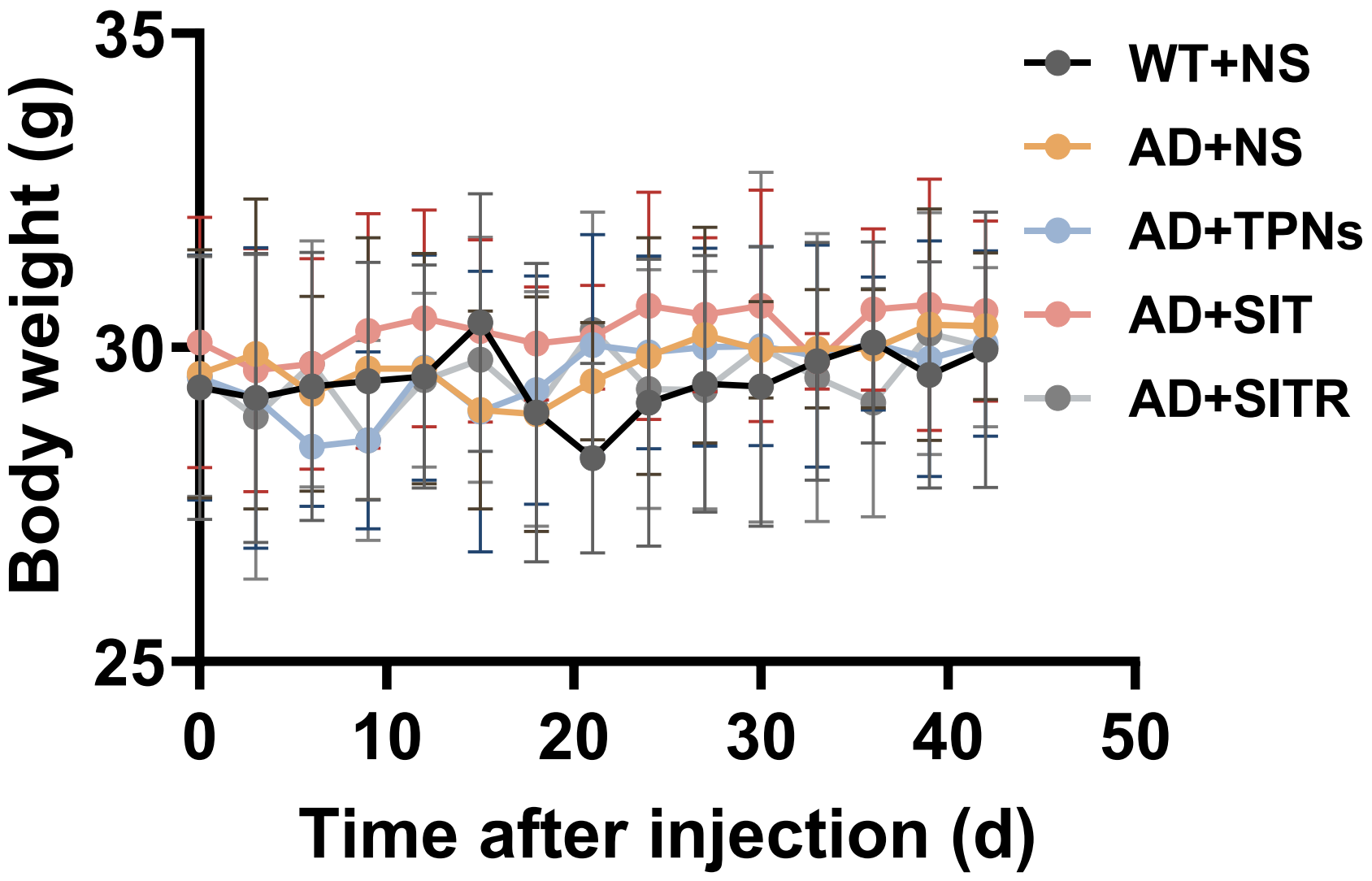

Figure S23. **Body weight changes during treatment.** Body weight changes during treatment in different groups during the 6-week treatment period. Data are presented as mean ± SD (*n* = 5 mice per group). Repeated-measures two-way ANOVA followed by Tukey's multiple-comparison test. No statistically significant differences were observed among groups ($p \geq 0.05$).

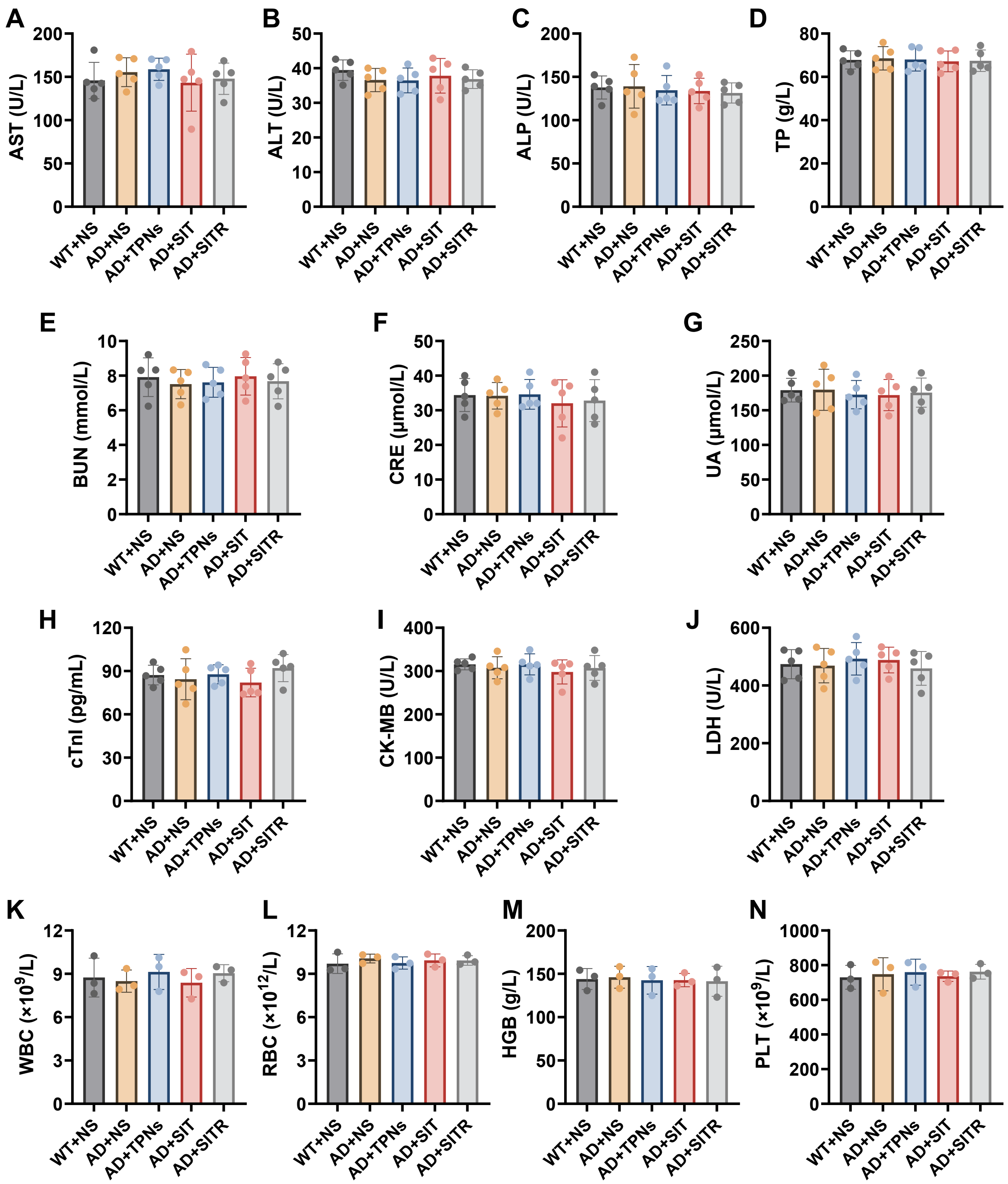

Figure S24. Evaluation of serum biochemical, cardiac injury-related, and hematological parameters after treatment. (**A–D**) Liver-related serum biochemical parameters, including aspartate aminotransferase (AST, **A**), alanine aminotransferase (ALT, **B**), alkaline phosphatase (ALP, **C**), and total protein (TP, **D**). (**E–G**) Renal function-related parameters, including blood urea nitrogen (BUN, **E**), creatinine (CRE, **F**), and uric acid (UA, **G**). (**H–J**) Serum cardiac

injury-related markers, including cardiac troponin I (cTnI, **H**), creatine kinase-MB (CK-MB, **I**), and lactate dehydrogenase (LDH, **J**). (**K–N**) Hematological parameters, including white blood cells (WBC, **K**), red blood cells (RBC, **L**), hemoglobin (HGB, **M**), and platelets (PLT, **N**). Data are presented as mean ± SD. For serum biochemical and cardiac injury-related markers, $n = 5$ mice per group. For hematological parameters, $n = 3$ mice per group. One-way ANOVA followed by Tukey's multiple comparison test. No statistically significant differences were observed among groups ($p \geq 0.05$).

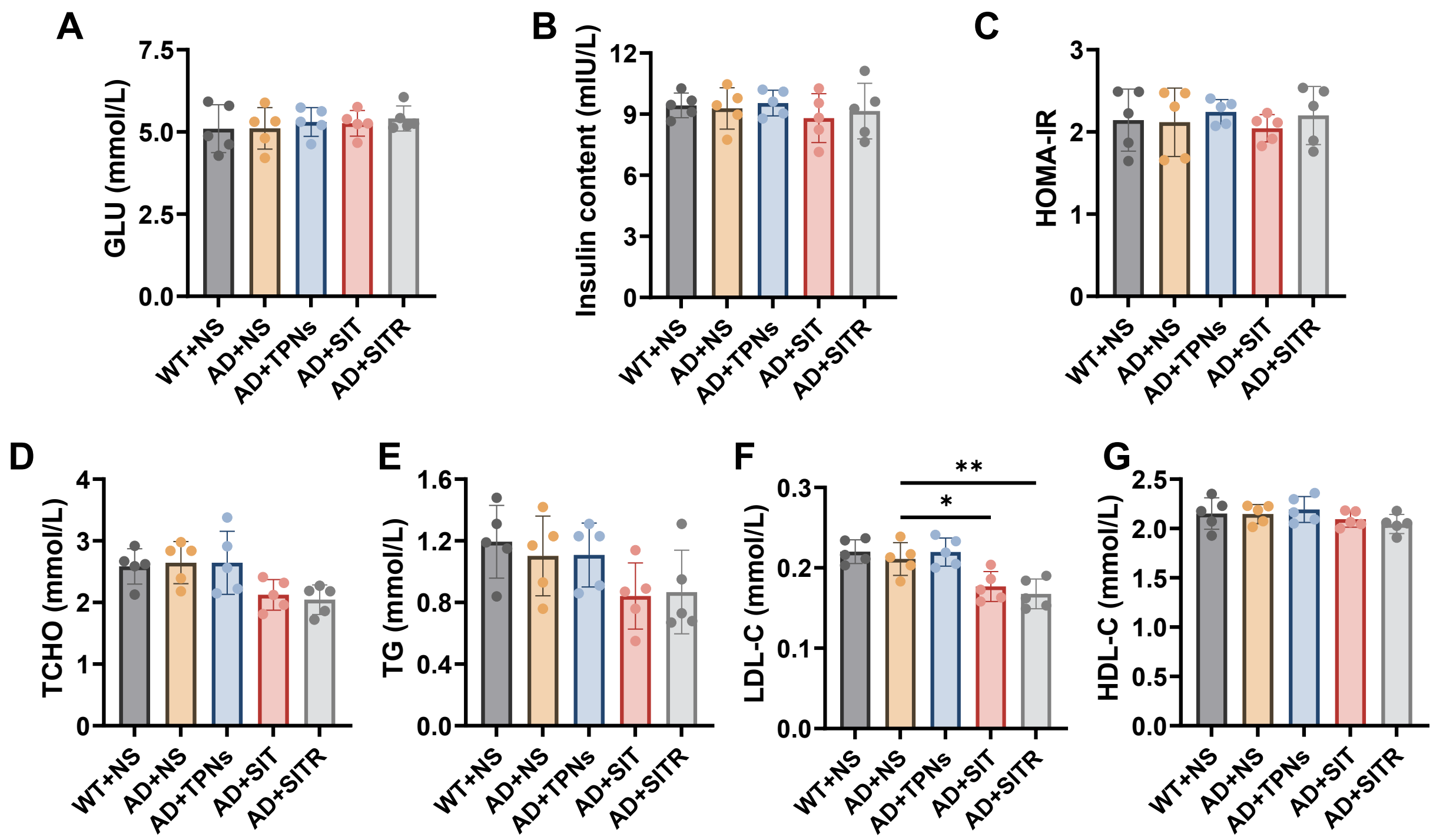

Figure S25. Evaluation of glucose and lipid metabolic parameters after treatment. (**A–C**) Glucose metabolism-related parameters, including fasting glucose (GLU, **A**), serum insulin (**B**), and homeostatic model assessment of insulin resistance (HOMA-IR, **C**). (**D–G**) Serum lipid profiles, including total cholesterol (TCHO, **D**), triglycerides (TG, **E**), low-density lipoprotein cholesterol (LDL-C, **F**), and high-density lipoprotein cholesterol (HDL-C, **G**). Data are presented as mean ± SD (n = 5 mice per group). One-way ANOVA followed by Tukey's multiple comparison test. No statistically significant differences were detected among groups in (A–E and G) ($p \geq 0.05$). For (F), *$p < 0.05$ indicates AD+SIT versus the AD+NS, and **$p < 0.01$ indicates AD+SITR versus the AD+NS.

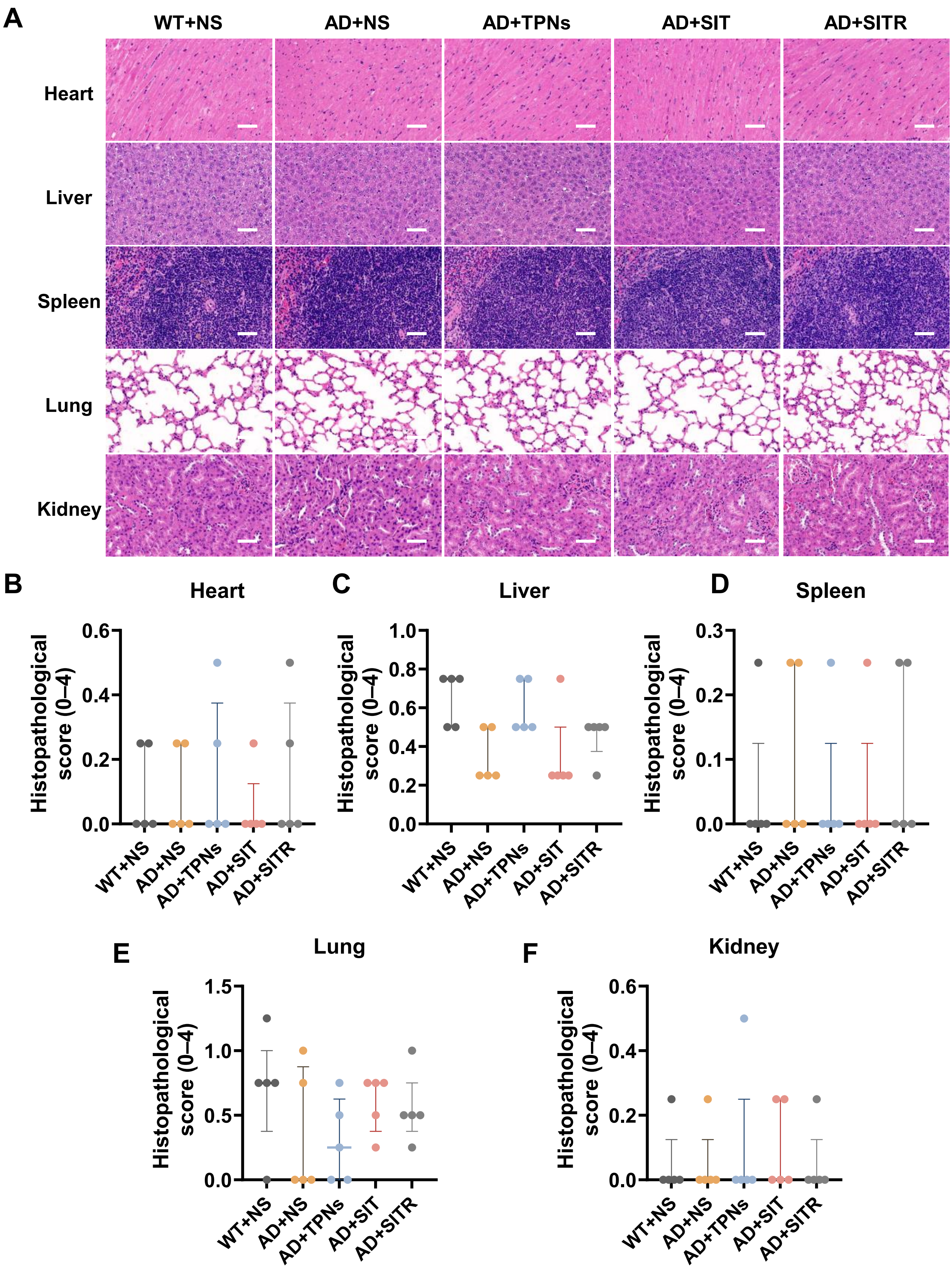

Figure S26. Histopathological assessment of major organs after treatment. (A)

Representative hematoxylin and eosin (H&E)-stained images of the heart, liver, spleen, lungs, and kidneys from mice in different treatment groups. Scale bar = 50 μm. (**B–F**) Semi-quantitative histopathological scores of the heart (**B**), liver (**C**), spleen (**D**), lungs (**E**), and kidneys (**F**), calculated based on organ-specific histological alterations. Lesions were graded on a scale from 0 (absent) to 4 (severe). Data are presented as median and interquartile range (IQR) (n = 5 mice per group). Kruskal–Wallis test followed by Dunn's multiple comparison test. No statistically significant differences were detected among groups for any organ ($p \geq 0.05$).

Table S1. Polydispersity index (PDI) of the siPCSK9/siTRIB3@TPN (SIT) complex at different siRNA loading concentrations.

Feeding siRNA in SIT	PDI
**0**	0.12 ± 0.01
**2μM**	0.10 ± 0.07
**4μM**	0.15 ± 0.04
**8μM**	0.12 ± 0.06

Table S2. Sequences of synthetic siRNAs

siRNA Name	Sense Strand (5' to 3')	Antisense Strand (5' to 3')
**siPCSK9-1**	GAGGCUACAGAUUGAACAATT	UUGUUCAAUCUGUAGCCUCTT
**siPCSK9-2**	GGUCCUUCAGAGCAGGUCATT	UGACCUGCUCUGAAGGACCTT
**siPCSK9-3**	GCACAUGCUUCAUGUCACATT	UGUGACAUGAAGCAUGUGCTT
**siTRIB3-1**	GGAAGAAACCGUUGGAGUUTT	AACUCCAACGGUUUCUUCCTT
**siTRIB3-2**	AGAGUACACCUGCAAGGUGUATT	UACACCUUGCAGGUGUACUCUTT
**siTRIB3-3**	CCAAGUGUCCAGUCCUAAATT	UUUAGGACUGGACACUUGGTT
**siNC**	UUCUCCGAACGUGUCACGUTT	ACGUGACACGUUCGGAGAATT

Table S3. Primer sequences used for reverse Transcription Quantitative Real-Time Polymerase Chain Reaction (RT-qPCR) analysis.

Gene symbol	Primer Sequence (5' → 3')
Pcsk9	F: CAGCGAGAGGACAGGCTATG; R: CTTGGTACAGTCGCCCTCTG
Lrp1	F: GCGGTGTGACAACGACAATG; R: CGTGTGTCTCGTCGCTGTAG
Trib3	F: CTGCGTCGCTTTGTCTTCAGCA; R: CTGAGTATCTCTGGTCCCACGT
Tjp1	F: TTTGTGATACAATACTGTGCCCT; R: ACAGAAATCGTGCTGATGTGC
Ocln	F: ACAGTCCAATGGCCTACTCC; R: ACTTCAGGCACCAGAGGTGT
Cldn5	F: TATGAATCTGTGCTGGCGCT; R: GTGCTACCCGTGCCTTAACT
Actb	F: GTGCTATGTTGCTCTAGACTTCG; R: ATGCCACAGGATTCCATACC
Il6	F: CAAAGCCAGAGTCCTTCAGAG; R: GTCCTTAGCCACTCCTTCTG
Il1b	F: GTGCTGTCGGACCCATATGA; R: TTGTCGTTGCTTGGTTCTCC
Tnf	F: ACTGAACTTCGGGGTGATTG; R: GCTTGGTGGTTTGCTACGAC
Ccl2	F: GGCTCAGCCAGATGCAGTTAAC; R: GCCTACTCATTGGGATCATCTTG
